# Operational research to support equitable non-communicable disease policy in low-income and middle-income countries in the sustainable development era: a scoping review

**DOI:** 10.1136/bmjgh-2019-002259

**Published:** 2020-06-30

**Authors:** Naomi Gibbs, Joseph Kwon, Julie Balen, Peter J Dodd

**Affiliations:** School of Health and Related Research, The University of Sheffield, Sheffield, UK

**Keywords:** health economics, health services research, public health, review, health policies and all other topics

## Abstract

**Introduction:**

Non-communicable diseases (NCDs) represent a growing health burden in low-income and middle-income countries (LMICs). Operational research (OR) has been used globally to support the design of effective and efficient public policies. Equity is emphasised in the Sustainable Development Goal (SDG) framework introduced in 2015 and can be analysed within OR studies.

**Methods:**

We systematically searched MEDLINE, Embase, Scopus and Web of Science for studies published between 2015 and 2018 at the intersection of five domains (OR, LMICs, NCDs, health and decision-making and/or policy-making). We categorised the type of policy intervention and described any concern for equity, which we defined as either analysis of differential impact by subgroups or, policy focus on disadvantaged groups or promoting universal health coverage (UHC).

**Results:**

A total of 149 papers met the inclusion criteria. The papers covered a number of policy types and a broad range of NCDs, although not in proportion to their relative disease burden. A concern for equity was demonstrated by 88 of the 149 papers (59%), with 8 (5%) demonstrating differential impact, 47 (32%) targeting disadvantaged groups, and 68 (46%) promoting UHC.

**Conclusion:**

Overall, OR for NCD health policy in the SDG era is being applied to a diverse set of interventions and conditions across LMICs and researchers appear to be concerned with equity. However, the current focus of published research does not fully reflect population needs and the analysis of differential impact within populations is rare.

**Key questions**

**What is already known?**

- Operational research (OR) has been widely used to aid decision makers in low-income and middle-income countries (LMICs) to prioritise health interventions.
- There is a growing and disproportionate burden of non-communicable disease (NCD) on LMICs, and on the most vulnerable populations within these countries.
- The Sustainable Development Goals (SDGs) emphasised the need for interventions which target NCDs while simultaneously promoting equity.

**What are the new findings?**

- The 149 papers included in this review demonstrated that OR methods are being used to provide valuable evidence across a wide range of NCD disease burdens and policy options in the SDG era.
- The number of papers were not proportional to the LMIC disease burden attributable to each NCD.
- OR in the SDG era demonstrates a strong concern for equity, although this was often limited to a focus on disadvantaged groups or universal coverage without robust consideration of differential impact.

**What do the new findings imply?**

- OR methods continue to provide valuable evidence across a broad range of policy options and NCD disease burdens in LMICs.
- In future, operational researchers should consider including the differential impact of the policies they are evaluating, thereby ensuring that equity remains a central concern.

## Introduction

Non-communicable diseases (NCDs) account for 73% of global deaths and 62% of global disease burden in 2017, increasing from 48% to 62% respectively in 2000.^1^ This rising burden has meant that the prevention and control of NCDs is now a major global health priority. Low-income and middle-income countries (LMICs) suffer the majority of global NCD-related mortality and morbidity: in 2016, an estimated 78% of global NCD-related deaths, and 82% of morbidity, occurred in LMICs.^2 3^ Moreover, the NCD burden is expected to further increase in LMICs as they continue through the epidemiological transition with its emergence of chronic health issues and NCDs,^4^ driven in part by urbanisation, and increasing levels of behavioural risk factors such as smoking, physical inactivity and nutritionally poor diets^5 6^ as well as an ageing population. As NCDs disproportionately affect working-age adults, increasing NCD burden has significant implications for the socioeconomic development of these countries.^7^

Despite these growing challenges, there are a wide range of interventions available to policymakers and healthcare practitioners to reduce the burden of NCDs in LMICs. An effective ‘package’ of measures may encompass diverse approaches including: fiscal policies and regulations to reduce the key modifiable risk factors such as tobacco use and unhealthy diets; population-wide screening to achieve early diagnosis and treatment of NCDs and access to effective and affordable individual-level healthcare interventions.^7 8^ However, LMIC governments face unique challenges in financing and implementing many of these solutions. Their healthcare systems often have weak infrastructure for the management of chronic conditions associated with NCDs.^9^ Public policies effective in reducing risk factors for NCDs elsewhere may require adaptations to suit local contexts or risk being patchily implemented and poorly enforced.^10–13^ Moreover, despite improvements in population health insurance coverage in certain LMICs, the service coverage and the level of financial protection provided by the insurance are often limited.^14^

The discipline of operational research (OR) can provide quantitative evidence to guide scale-up of interventions for NCDs^15^ by projecting the cost, health and resource implications of strategies that aim to compare interventions, improve healthcare delivery and which may improve health/healthcare equity within the constraints of the relevant settings.^16 17^ OR models can synthesise diverse evidence types, including context-relevant epidemiological and economic data, to compare multiple strategies affecting different system components, and extrapolate natural disease history and intervention effects to estimate long-term impact.^18^ OR methods can also account for capacity constraints in existing policy and healthcare infrastructure, making them well-suited to the evaluation of intervention delivery in low-resource contexts.^19^

A key characteristic of NCDs is their strong link with health (in)equity in LMIC contexts. NCDs are important contributors to (within-country) health inequity insofar as modifiable risk factors for NCDs are typically concentrated in population subgroups who have low socioeconomic status (SES) and/or live in poor or marginalised communities.^20^ The weak financial protection offered by health insurance schemes in LMICs means that NCDs often result in catastrophically high out-of-pocket (OOP) expenditures for already impoverished and marginalised groups.^21 22^ Therefore, OR may demonstrate an equity focus through: (i) consideration of differential impact by socially determined subgroups; (ii) the deliberate targeting of policies towards vulnerable groups and/or (iii) policies which extend access to healthcare (eg, universal provision) thus improving financial protection.

Previous reviews include Bradley *et al*,^17^ who provided an overview of 44 OR studies in LMICs (a subset of 1099 studies overall) published by 2014, covering both communicable diseases and NCDs and equity considerations. They found health equity consideration was primarily manifested in a concern for *healthcare* equity, particularly in relation to hard-to-reach or disadvantaged subgroups. For OR to have impact, they recommended researchers embed stakeholder engagement, use contextually appropriate data and vary communication channels for outputs. An earlier, 2006, review by Mulligan *et al*^23^ concentrated on economic evaluations of NCD interventions in LMICs. They classified the 32 included studies by 12 disease areas and 3 application areas (diagnosis, prevention and treatment) and assessed their methodological quality.

The 2015 Sustainable Development Goals (SDGs) include good health and well-being (SDG 3) and emphasise equity throughout. Given these goals, and the particular challenges posed by NCDs in LMICs, we investigated the use of OR in informing NCD-related health policy in LMICs, and whether a concern for equity informed the analyses. Here, we present results of a scoping review of studies published in peer-reviewed journals during the SDG era, from January 2015 to May 2018, which apply quantitative OR methods to evaluate policies for NCD prevention and treatment in LMICs. We provide a narrative summary by key themes, including geographical setting, policy/intervention type and disease area, with attention to if/how OR methods have been used to support a concern for equity.

## Methods

This scoping review follows a systematic search strategy and analysis to provide a broad overview of the size and scope of the literature. A scoping review can provide a preliminary assessment of the literature without the requirement of formal quality appraisals required by systematic literature reviews for the synthesis of evidence.^24 25^ This approach is able to accommodate both diverse study designs and broad topics such as OR, NCDs and equity. The scoping review followed Preferred Reporting Items for Systematic Reviews and Meta-Analyses (PRISMA) guidelines for scoping reviews^26^ and was registered on PROSPERO reference CRD42018096803.

### Search strategy

Web of Science, Scopus, Embase and MEDLINE were searched to cover both health and social science. Free-text searches were undertaken for Web of Science and Scopus. Free text plus Emtree and MESH subject headings were used for Embase and MEDLINE, respectively. The database free-text search strategy drew on the work of Bradley *et al*,^17^ which searched papers published between 2000 and 2014 at the intersection of four domains: operational research, LMICs, health and decision-making/policy-making. To these a fifth domain was added for NCDs. The current search included papers published between 1 January 2015 and 10 May 2018. An English language filter was applied to all results. Full search strategies are included in online supplementary appendix 1.

10.1136/bmjgh-2019-002259.supp1 Supplementary data

### Study inclusion

Two researchers (NG and JK) independently sifted all articles, first according to title and abstract and second using the full text. The inclusion criteria drew on those used by Bradley *et al*^17^ with the additional specification of NCDs.

The first criteria was for the study to use OR methods, including: optimisation models (eg, linear and non-linear programming, goal programming, location allocation models); simulation models (eg, discrete event, agent-based); dynamic state transition models (eg, Markov transition) and static cohort-level models (eg, decision tree model).

Second, the study must explore a decision-making/policy-making problem compatible with OR. This could be: several competing interventions or policy options are compared to propose the best/optimal strategy (including comparing current status quo vs a new option); outcomes are modelled for competing intervention or policy scenarios/options, including disease burden, budget impact and cost-effectiveness; issues of logistics, supply chain, distribution, scheduling are explored, including studies that highlight operational inefficiencies or poor performance; what if scenarios are tested (eg, what if cancer screening coverage was increased).

Third, the study explores an OR problem with a health or healthcare focus. This can include public or population health (eg, public policy affecting population health, mass screening for disease diagnosis and treatment, relative cost-effectiveness of two or more healthcare interventions) and/or healthcare delivery (eg, efficiency, efficacy and cost of delivering given healthcare service).

Fourth, the study focuses on a low-income or middle-income setting, either specific country or within-country or supranational region.

Finally, the study must focus on an NCD. The list of NCDs was taken from the WHO Global Health Estimates^3^ and Global Burden of Disease project.^1^ Conditions which are not categorised as NCDs but have substantial impact on the NCD burden were also included. For example, human papillomavirus infection and nutritional deficiencies are important causes of cervical cancer and increased cardiovascular risk, respectively. Further detail is included in online supplementary appendix 1.

At the end of each screening stage, the researchers met to compare results and resolve decision discrepancies. Ambiguous cases were arbitrated by the other authors (JB and PD).

### Data extraction

Standard bibliographic information was extracted alongside data for the following topics: geographic coverage, policy area, disease/risk area and equity consideration.

### Narrative synthesis

We conducted a narrative synthesis of study characteristics with a special focus on two themes, namely: (i) application area of policy/intervention and (ii) equity consideration adopted by the study (if any). Both of these were further broken down and/or categorised as follows:

#### Policy/Intervention application area

Given the variety of global health solutions (ranging from national fiscal policies to individual-level healthcare), studies were inductively categorised into the following application areas, which arose through reviewing of the papers:

- *Public health policy*: policy working via a *non-healthcare* mechanism to modify key risk factors for NCDs; examples include taxation of sugar-sweetened beverages and regulations to create smoke-free areas.
- *Population screening*: screening of general populations at risk of a specific NCD before provision of relevant healthcare to diagnosed persons; examples include national cervical cancer screening programmes and diabetes screening in primary care.
- Healthcare provision
  1. *Access*: provision of healthcare to defined patient groups who previously had no access to a comparable standard of care; examples include charitable provision of surgery to patients with cancer who otherwise have no access to oncology treatment and provision of dialysis to patients with end-stage renal disease who otherwise would have received basic supportive care only.
  2. *Comparative*: effectiveness and/or cost-effectiveness studies of two or more alternative healthcare interventions of comparable standards for a defined patient group; examples include comparison of two chemotherapy regimens for patients with breast cancer and comparison of two insulin-based treatments for patients with diabetes to achieve superior control of given disease and/or lower intervention cost.

Within each application area, the included studies were further grouped by risk area (for studies under category A) or targeted disease area (for studies under categories B and C). Application areas were mutually exclusive.

#### Equity consideration

Studies were categorised by whether and how they demonstrated a concern for equity, this categorisation was inductive:

- *Differential subgroup impact*: the study evaluates the differential impact of intervention outcomes on population subgroups delineated by social, economic, demographic and geographic or any other predisposing environmental factor beyond individual control. The outcomes may include health (eg, life expectancy, disability-adjusted life years (DALY)), healthcare access or financial risk protection from NCD-induced healthcare expenditure.
- *Disadvantaged group targeting*: the intervention targets healthcare access and/or health improvement in a population group deemed to be socially disadvantaged; examples of such groups include women, infants and children, the elderly and rural populations.
- *Universal coverage of healthcare*: the intervention aims to expand access to preventive or treatment services.
  - *Preventive services*: widening the provision of preventative healthcare. For example, a new national cancer screening programme is compared with no screening or opportunistic screening at healthcare centres, or where coverage levels are varied and evaluated within the study.
  - *Treatment services*: provision of clinical care to patients who previously had no access. We only include cases representing an improvement in coverage of public sector healthcare, that is, where the cost of relevant healthcare is at least partly borne by the government.

Studies under category (I) must explicitly highlight differential impact as an equity concern: simple stratification of results (eg, by age or sex) with no discussion of equity was deemed insufficient for inclusion. These equity categories are not mutually exclusive and a study was able to fall into multiple categories.

### Patient and public involvement

As this paper is a scoping review comprising an assessment of the academic literature, there was no direct patient and public engagement on the paper.

## Results

### Database search and study inclusion

**Figure 1:**
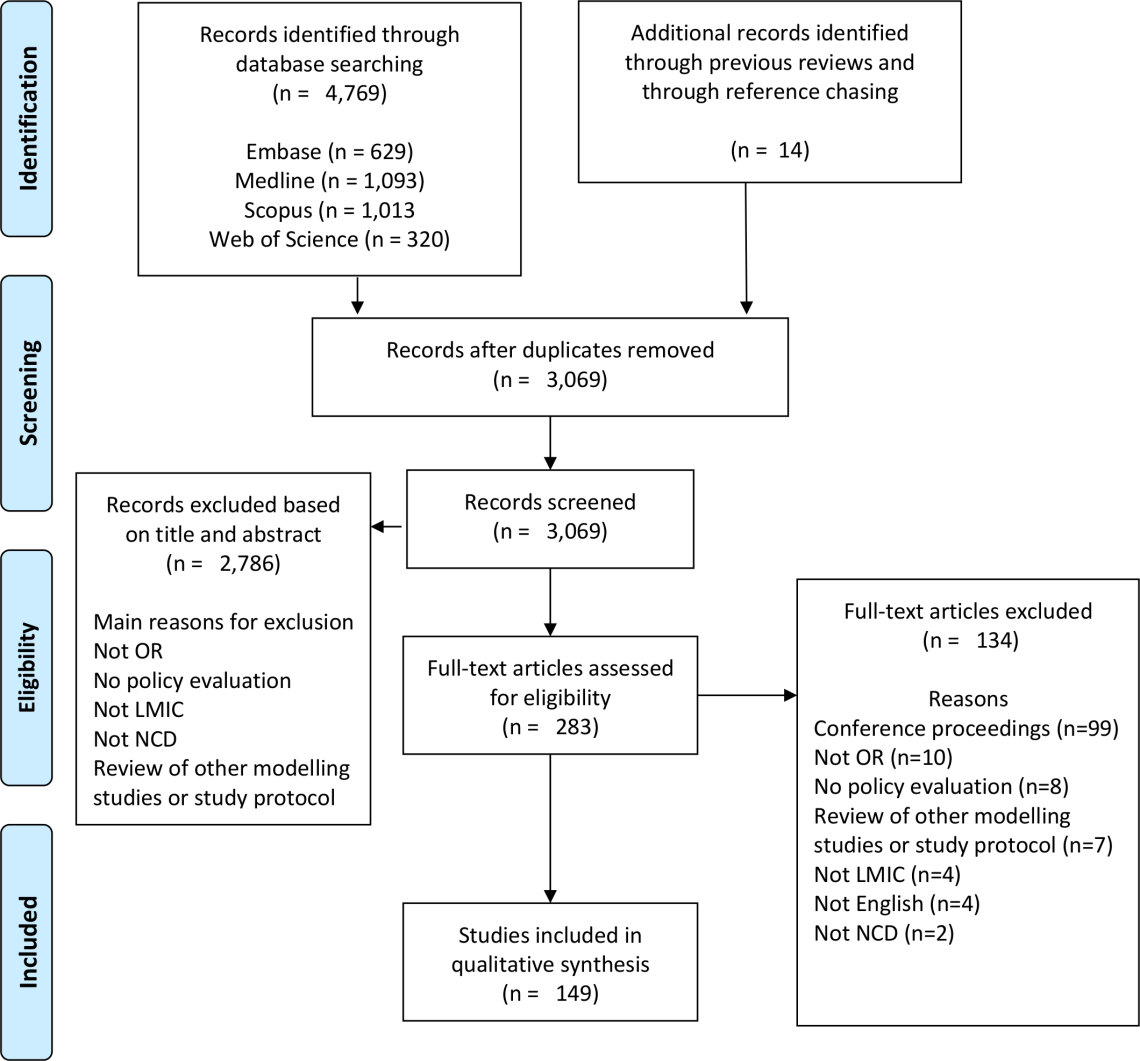
Preferred Reporting Items for Systematic Reviews and Meta-Analyses (PRISMA) flow chart. LMIC, low-income and middle-income countries; NCD, non-communicable disease; OR, operational research.

The PRISMA flow diagram illustrates the search process, including article screening and study inclusion (figure 1). The database search resulted in 3055 papers after duplicates were removed. After screening by title and abstract, 269 papers remained for the full-text sift. An additional 134 papers were excluded at this stage and 14 articles were added via searching of previous reviews and reference chasing. This resulted in a final group of 149 articles for data extraction.

### Study characteristics

**Figure 2:**
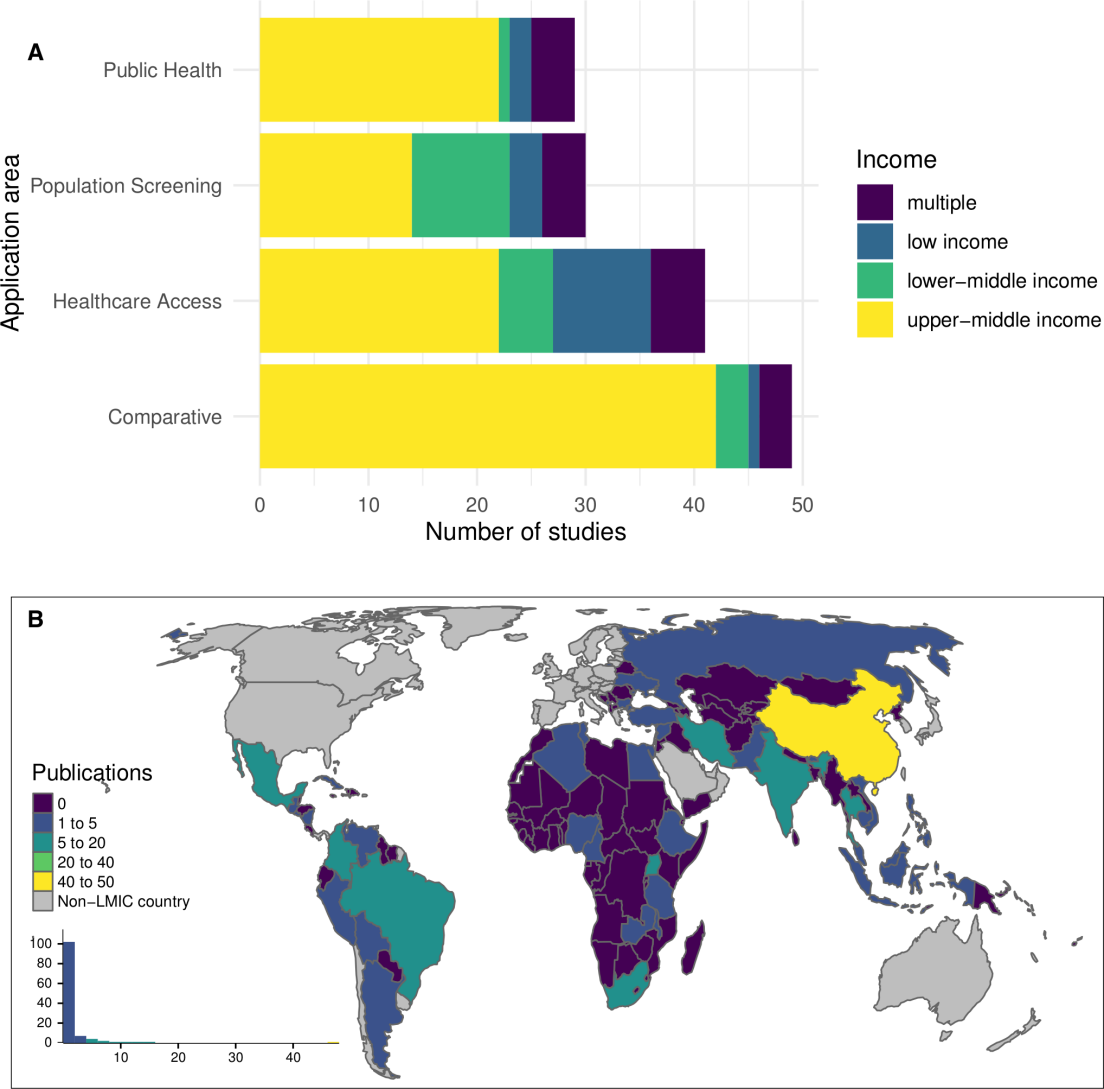
(A) Number of studies by application area, grouped according to income classification (multiple income category denotes studies including more than one country spanning more than one income classification). (B) geographical location of the target population. LMIC, low-income and middle-income countries.

There were 29 (19%) studies evaluating public health policies working via non-healthcare mechanisms to reduce key modifiable risk factors for NCDs and 30 (20%) that evaluated population screening interventions. Of the 90 (60%) studies that evaluated healthcare provision for defined patient groups, 41 were primarily concerned with providing access to new or higher-standard healthcare, while 49 included evaluation of strategies with a comparative standard of care. When classified by application area and income group of the country/countries included in the study, a very high proportion, 76% and 85%, of public health policy and comparative evaluation studies, respectively, incorporated only upper-middle-income countries (figure 2A). At the same time, few papers from across any application area included countries from multiple income categories. The papers covered a broad range of countries, the highest number of papers (47, 32%) address the Chinese context, followed by Thailand and Mexico each with 12 papers (8%) (figure 2B). The authors affiliated with institutions in high-income countries (HICs) were listed in 68% of the papers, while 15% of the papers did not include any author from the country studied. The authors affiliated with governmental departments, quasi-governmental institutions or specific hospitals in the country of interest were included in 44% of the papers.

#### Policy/Intervention application area

**Figure 3:**
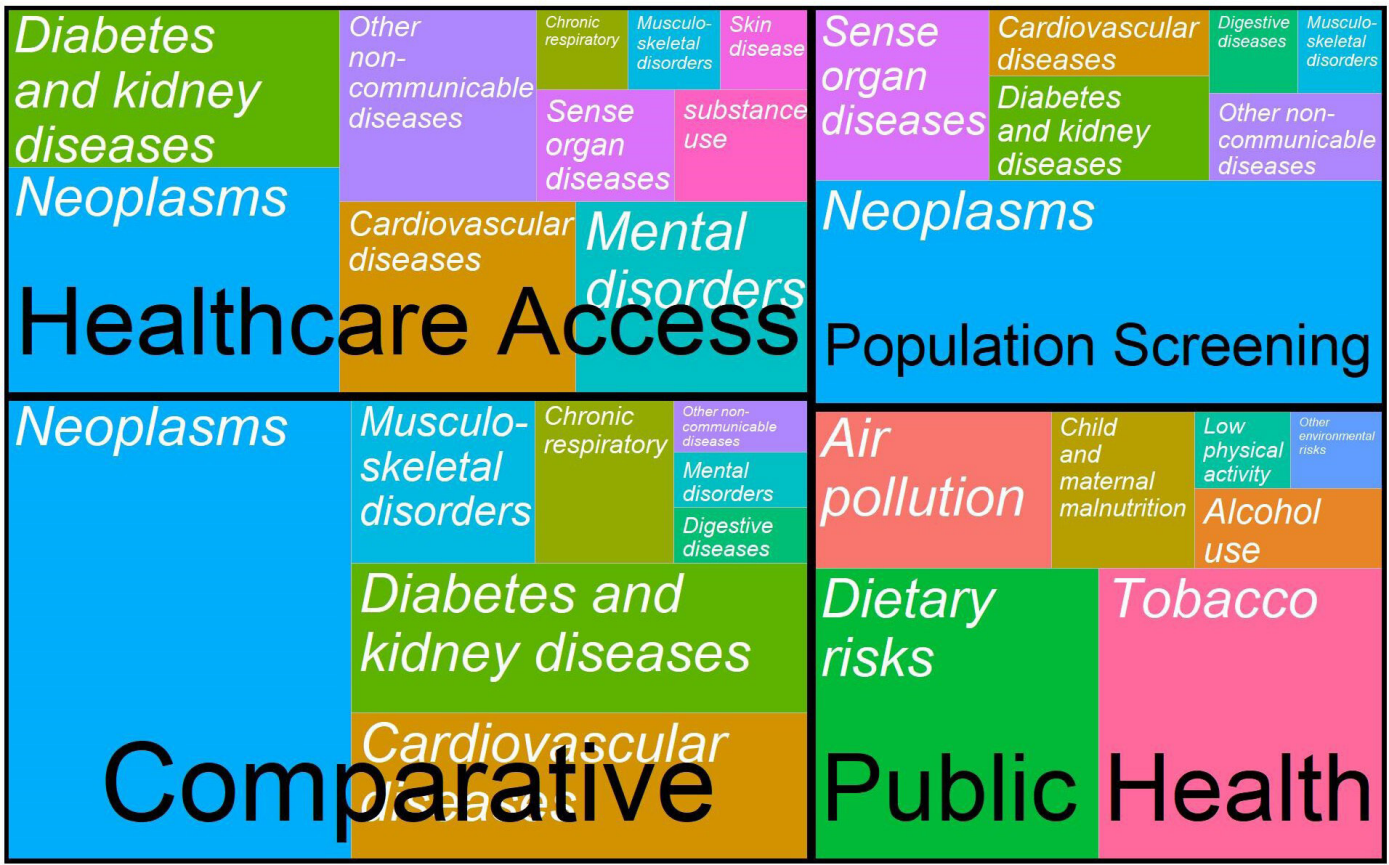
Disease/Risk type by application area. Public health papers categorised by risk factor, all others by disease area. Colours are not meaningful. Public health policies covering more than one risk area were counted in multiple categories.

The 149 included studies covered a broad range of risk factors and disease areas (figure 3). Neoplasms constitute a relatively large group across population screening, healthcare access and comparative studies. Cardiovascular disease and diabetes and kidney disease are also a strong focus. The risk factors with the greatest proportion of papers relate to dietary risks and tobacco, which link strongly to the aforementioned disease areas. A summary table referencing every included study and an expanded table detailing policy/intervention and comparator, equity consideration, and base-case evaluation results are included in online supplementary appendix 2, tables A and B.

10.1136/bmjgh-2019-002259.supp2 Supplementary data

**Figure 4:**
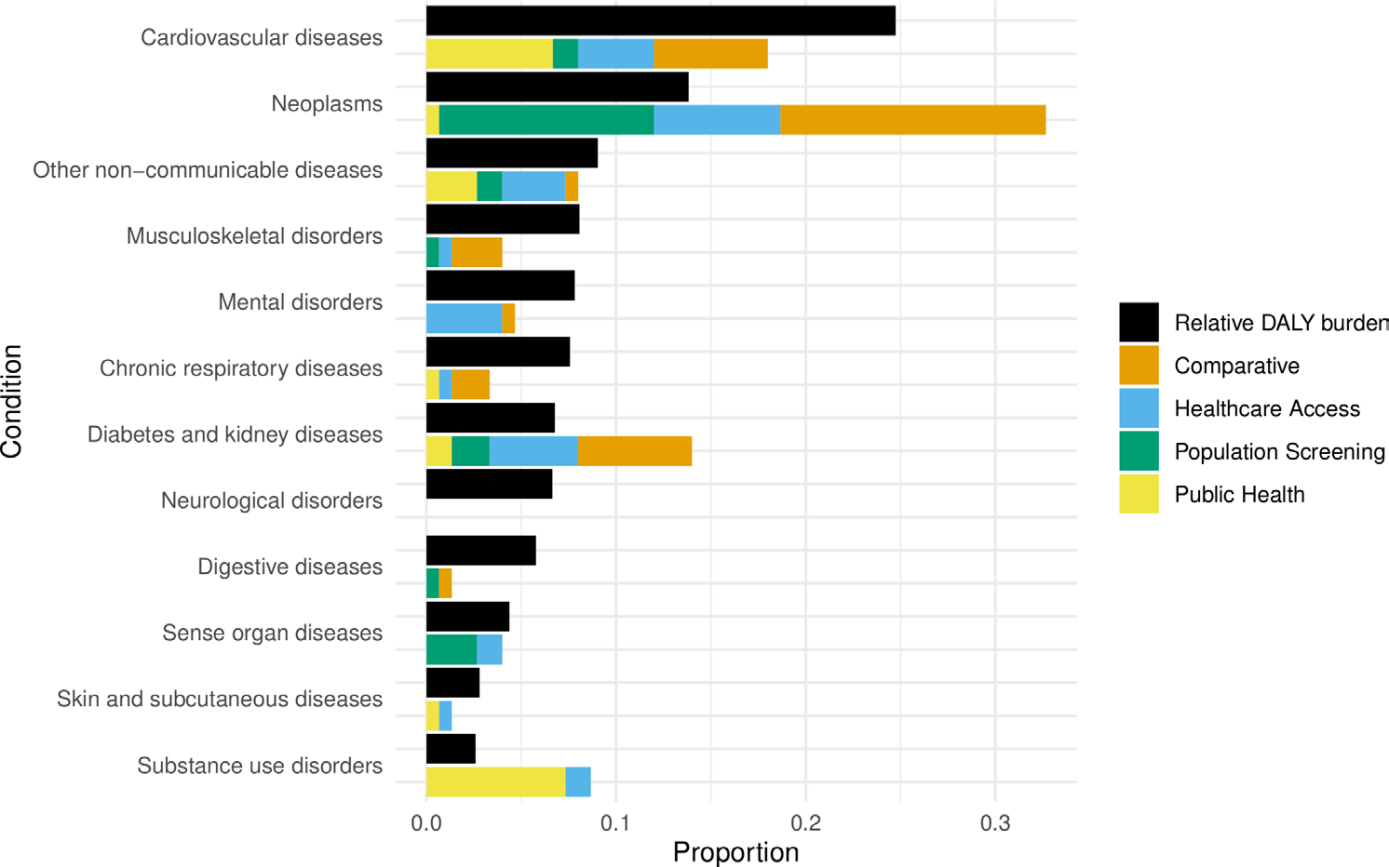
Comparison of reported disease burden as measured by disability-adjusted life years (DALYs) and number of publications. Multiple categories for each paper let the paper count in multiple categories.

When the proportion of NCD burden across LMICs, using Global Burden of Disease data,^1^ was compared with the proportion of papers in the review, the results showed that OR methods have been used across all NCD categories, with the exception of neurological disorders (figure 4). The greatest proportion of papers address neoplasms; these include a number of comparative studies which compare cancer drug A versus drug B and a number of screening programmes mainly focused on cervical cancer. Diabetes and kidney diseases and substance use disorders are also over-represented within the search results with a number of public health policies focused on tobacco and alcohol. Neurological disorders are unrepresented.

#### Equity consideration

**Figure 5:**
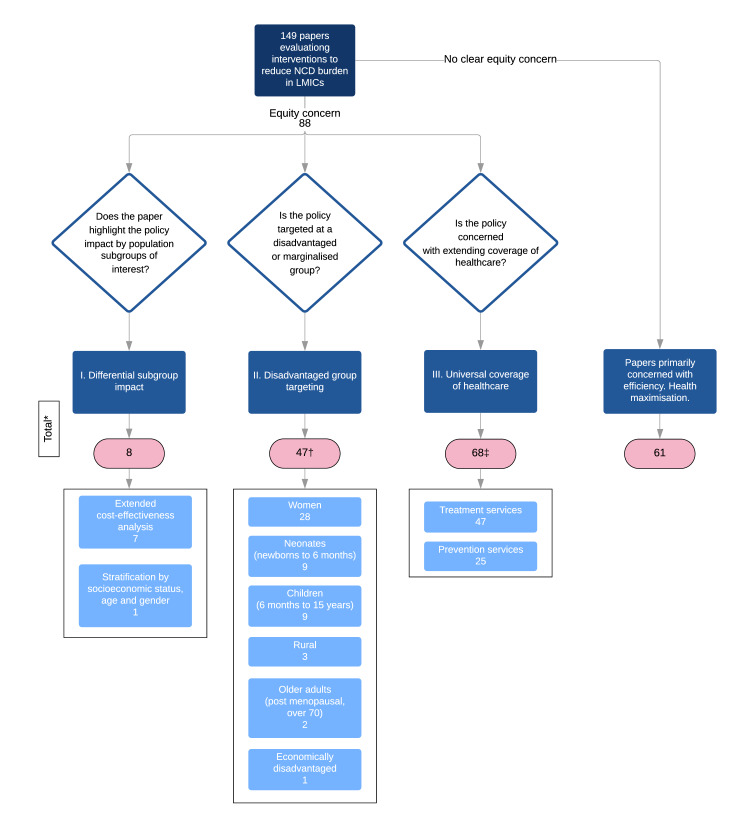
Equity categorisation. *The sum of the totals is >149 as a number of papers are included in more than one equity category. †The sum of disadvantaged groups is >47 as a number of papers cover more than one disadvantaged group. ‡The sum of treatment and preventions services is >68 as four papers cover both. LMIC, low-income and middle-income countries; NCD, non-communicable disease.

Of the 149 papers, 88 (59%) fell into at least one of the three equity categories (figure 5).

##### Differential subgroup impact

The eight subgroup impact papers mainly comprised extended cost-effectiveness analysis (ECEA) (n=7). ECEA requires the inclusion of OOP expenditure and at least one measure of financial risk protection, providing an explicit link between health and poverty (catastrophic health expenditure, cases of poverty averted, value of insurance).^27^ These measures, along with health outcome measures, are reported by income or wealth quintiles. Wealth quintiles are calculated using a composite measure of household asset ownership (eg, TV, fridge, etc) and access to amenities (eg, water and sanitation). Wealth quintiles were used in studies on Ethiopia (n=2), Uganda (n=1) and India (n=1); income quintiles were used in China (n=2) and South Africa (n=1). A range of health outcomes were reported including deaths averted (n=4), life years gained (n=1), DALYs (n=1) and health-adjusted life years gained (n=1). The only study demonstrating subgroup impact which did not follow an ECEA methodology evaluated a sugar-sweetened beverage (SSB) tax in Mexico reporting the health outcome (percentage change in body mass index) by SES (low, medium and high), age and sex.

##### Disadvantaged group targeting

A total of 47 papers evaluated policies specifically targeted at socially disadvantaged groups. The most populous group was women (n=28), followed by neonates (n=9), children (n=9), rural dwellers (n=3), older adults (n=2) and economically disadvantaged (n=1). Four of the papers concerned the intersection of two groups, including rural and neonatal (n=2), rural and women (n=1) and women and neonatal (n=1).

##### Universal health coverage

All 41 of the healthcare provision access papers fell into this category as did an additional 4 comparative papers which examined different funding strategies and 23 of the population screening papers, totalling 68. Screening papers which did not fall into this category were either concerned with the cost-effectiveness of comparable screening strategies (n=4), budget distribution between screening and treatment (n=1) or were paid for exclusively by private insurance (n=1).

#### Papers demonstrating an equity focus including different approaches to equity, according to policy/intervention application area

The intersection between application area and equity approach taken is included in online supplementary appendix 2, table C. In summary, of the public health papers, seven (24%) incorporated equity, three via the demonstration of equity impact (two were ECEAs and one stratified outcomes by SES, sex and age) and four via a focus on disadvantaged groups, namely children (n=2), neonates (n=1) and women and neonates combined (n=1).

Population screening demonstrated concern for equity in 29 papers (97%) mainly via a focus on disadvantaged groups, of which the majority were women (n=15), and extending preventative healthcare services via universal provision of screening policies. The screening interventions were dominated by cervical cancer (n=8), congenital disease (n=6) and breast cancer (n=4).

All 41 healthcare provision access studies by definition fell into the universal health coverage, however, many papers also fell into the other equity categories. Four papers used ECEA to demonstrate the impact of the healthcare treatment by income/wealth quintiles including charitable surgery in Uganda, epilepsy treatment in India, mental health treatment in Ethiopia and a package of diverse healthcare treatments in Ethiopia. There was also range of disadvantaged groups covered across 12 of the papers including children (n=5), women (n=4), the elderly (n=1), neonates (n=1) and lower socioeconomic groups (n=1).

The healthcare provision comparative studies had the lowest percentage of papers considering equity with just 11 (23%). Eight covered disadvantaged groups, including women (n=6) and children (n=2). The four papers in this application area which evaluated alternative cancer treatments demonstrated concern for universal coverage by incorporating scenarios involving different levels of public funding for the treatment.

## Discussion

The range of studies using quantitative OR methods (broadly understood) demonstrates the breadth of public health and clinical interventions available to policymakers for addressing the burden of NCDs in LMICs. The much greater number of OR studies evaluating NCD interventions included in this review, compared with previous reviews,^17 23^ suggests that researchers have responded to previous conclusions which highlighted the gap in NCD research in LMICs. However, the focus of the published studies was not proportional to the disease burden reported by the Global Burden of Disease Study for LMICs. The disproportionate focus on cancers may reflect an international funder-driven agenda which supports cancer screening programmes for women and girls.^28 29^ The large number of papers relating to substance use, including tobacco and alcohol papers, may also reflect influence of the WHO’s Framework Convention on Tobacco Control and global reporting of alcohol harm.^30 31^ The topics of published papers also mirror the primarily Northern-based study funding and a research agenda influenced by HIC research partners (68% of papers listed authors affiliated to institutions in HICs).

Equity was considered in 59% of the 149 papers included in the review. This supports the renewed focus on health equity as it relates to differential impact between subpopulations, targeting of disadvantaged groups and extending universal healthcare. In a previous scoping review of OR studies only 4% of studies included an equity consideration and these studies focused primarily on access to healthcare.^17^ Although their review differed from ours, it seems plausible that operational researchers are responding to the increasing global focus on equity, evident in the SDGs.

Despite this strong focus on equity only eight papers explored differential impact, seven of which used extended cost-effectiveness analysis. These papers, across all three application areas, demonstrate the prominence of ECEA as a methodological approach and its flexibility of application to diverse health interventions. However, a number of papers which modelled universal upstream public health interventions did not incorporate a concern for differential impact, including SSB taxes, unhealthy goods taxes and salt reduction policies,^11 13 32–35^ acknowledged in the papers as a key limitation. Given the importance of these upstream policies in reducing risk factors for NCDs, often making use of fiscal measures which are likely to impact the poor more severely than the rich, the lack of consideration of differential impact is a clear weakness.

The finding that 44% of papers included authors affiliated with governmental departments, quasi-governmental institutions or specific hospitals in the country of interest infers a degree of stakeholder engagement in OR. However, it should be noted that 15% of papers had no author at all from the country of study so there is still significant progress to be made in embedding research in its local context.

We argue that OR shows much potential to improve the quality of decision-making and guide improvements in population health and equity. Researchers should attempt to incorporate differential impact within their research wherever possible, although we acknowledge that barriers exist, including data availability, capacity constraints and researcher familiarity with methods. Understanding which groups are of interest, or indeed which intersectionalities (eg, women and rural), is critical to the pursuit of equity and is highly context-dependent. Closer collaboration between researchers and decision makers and implementers would enable a deeper and more nuanced understanding of the equity impact of the policy and its implementation.

Greater stakeholder engagement would also strengthen the development and validation of models, and the translation of findings into policy and practice. Further research into the embeddedness of OR research in local decision-making processes would provide greater insight into the role that local stakeholders play in setting the research agenda as well as in the research process itself and in uptake of key findings.

To the best of our knowledge, this scoping review is the most comprehensive one to date covering all approaches to reduce NCD burden and promote health and healthcare equity in the SDG era. However, there are several limitations to this research, which in part reflect the nature of a scoping review. Databases were chosen to cover medicine and social science, but engineering databases may have provided additional papers. The search period was deliberately designed to provide a cross-sectional snapshot of the SDG era (2015–2018), however this prevents the consideration of OR applications before this period and of trends over time. Important studies may have been missed due to date range alone, for example, those evaluating excise tax on SSB in India^33^ and South Africa.^11^ The limitation to English language papers may have biassed the results, in particular the conclusion that most research has been completed in HICs. There was no formal quality assessment for included studies due to the very diverse nature of their aims and methods. There was also limited discussion on the technical aspects of OR models due to our focus on setting, application areas and equity categories in this manuscript.

In summary, this review found that OR is being used to evaluate a broad range of policies across a range of NCDs in LMICs, although the focus of the papers was disproportionate to the relative disease burden in these countries. A majority of papers incorporated a concern for equity, although this is primarily through targeted policies and expansion of healthcare. The included papers demonstrated that OR methods can be used to illustrate differential impact, highlighting vulnerable groups, across a range of application areas and disease types. This however, appears to currently be an underused approach in relation to OR on NCD policy in LMICs.
