# Supplementary material for: Operational research to support equitable non-communicable disease policy in low-income and middle-income countries in the sustainable development era: a scoping review
Source: BMJ Glob Health. 2020 Jun 30;5(6):e002259. doi: 10.1136/bmjgh-2019-002259 (PMC7328817; doi:10.1136/bmjgh-2019-002259)
Supplement: Supplementary data [file bmjgh-2019-002259supp001.pdf]

Contents

Appendix 1: ... 1
Inclusion criteria for non-communicable disease ... 1
Search Strategy ... 2
Web of Science ... 2
Scopus ... 3
Embase ... 3
Medline ... 5

Appendix 1

Inclusion criteria for non-communicable disease

As outlined in the manuscript the list of non-communicable diseases was taken from the WHO Global Health Estimates (WHO, 2016) and Global Burden of Disease project (IHME, 2018).

During the review process all four authors spent some time discussing which papers should be included based on the health condition of focus in the paper. One contentious area may be the inclusion of child and maternal malnutrition, under which three studies were included in the review (Botteman and Detzel, 2015; Dainelli et al., 2017; Vosti et al., 2015). The WHO Global Health Estimates and GBD places nutritional deficiencies under "Communicable, maternal, perinatal and nutritional conditions", rather than non-communicable diseases. However, given the central role played by in-utero and early-life diet on the non-communicable disease burden in later life (WHO, 2018), as well as its potentially inequitable impact across population subgroups (Di Cesare et al., 2013), the authors deemed it reasonable to include the above studies of nutritional interventions in the review. There were other conditions, such as HPV infection and acute rheumatic fever, which are not non-communicable diseases but were included due to their substantial impact on the non-communicable disease burden - through cervical cancer (Guerrero et al., 2015; Levin et al., 2015; Mo et al., 2017; Setiawan et al., 2016) and rheumatic heart disease (Watkins et al., 2015; 2016), respectively.

Botteman M, Detzel P, 2017, *see reference 7 in manuscript Appendix Reference*
Dainelli L, et al., 2017, *see reference 8 in manuscript Appendix Reference*
Vosti SA, et al., 2015, *see reference 9 in manuscript Appendix Reference*
Di Cesare M, Khang Y-H, Asaria P, et al., 2013, *Inequalities in non-communicable diseases and effective responses*, The Lancet, 381(9866): 585-97
Guerrero AM, et al., 2015, *see reference 48 in manuscript Appendix Reference*
Levin CE, et al., 2015, *see reference 49 in manuscript Appendix Reference*
Mo X, et al., 2017, *see reference 50 in manuscript Appendix Reference*
Setiawan D, et al., 2016, *see reference 51 in manuscript Appendix Reference*
Watkins DA, et al., 2015, *see reference 94 in manuscript Appendix Reference*
Watkins DA, et al., 2016, *see reference 95 in manuscript Appendix Reference*

Search Strategy

Web of Science

Each category combined with “AND”. Restricted to English and year 2015 or later. Search run on 10th May 2018
Search 1 – model type<br>TS=("operation* research")OR<br>TS=(model* NEAR/5 (mathematical or quer* or inventory or scheduling or demand or forecast* or comput* or network or stochastic or decision* or delivery or simulation or optimi?ation or non-linear or linear or Markov or cost-effectiveness or agent-based)) OR<br>TS=(optimi?ation$ NEAR/5 (mathematical or nonlinear or non-linear or linear or network or discrete or multicriteria or multi-criteria or stochastic or problem or minimi?ation or location or allocation)) OR<br>TS=(simulation NEAR/3 (comput* or discrete or agent-based or system$))
Search 2 – geographic focus<br>TS=(“Developing countr*” or “low-income countr*” or “middle-income countr*” or “developing world” or “developing nation” or “low-resource setting*” or “resource-constrained setting*” or “resource-poor setting*” or “limited-resource setting*” or “resource-limited setting*” or “under-developed countr*” or “least-developed countr*” or “less-developed countr*” or LMIC* or Africa* or (Asia* NEAR/2 south) or (Asia NEAR/2 east) or “latin America*” or “central America*” or “south america*” or caribbean or “middle east” or AFGHANISTAN or ALBANIA or ALGERIA or “AMERICAN SAMOA” or ANGOLA or ARGENTINA or ARMENIA or AZERBAIJAN or BANGLADESH or BELARUS or BELIZE or BENIN or BHUTAN or BOLIVIA or “BOSNIA AND HERZEGOVINA” or BOTSWANA or BRAZIL or BULGARIA or “BURKINA FASO” or BURUNDI or “CABO VERDE” or CAMBODIA or CAMEROON or “CENTRAL AFRICAN REPUBLIC” or CHAD or CHINA or COLOMBIA or COMOROS or “CONGO, DEM. REP.” or “CONGO, REP.” or “COSTA RICA” or “COTE D'IVOIRE” or CUBA or DJIBOUTI or DOMINICA or “DOMINICAN REPUBLIC” or ECUADOR or “EGYPT” or “EL SALVADOR” or “EQUATORIAL GUINEA” or “ERITREA” or “ETHIOPIA” or FIJI or GABON or “THE GAMBIA” or GEORGIA or GHANA or GRENADA or GUATEMALA or GUINEA or GUINEA-BISSAU or GUYANA or HAITI or HONDURAS or INDIA or INDONESIA or IRAN or IRAQ or JAMAICA or JORDAN or KAZAKHSTAN or KENYA or KIRIBATI or KOREA or KOSOVO or KYRGYZ* or LAO or LAOS or LEBANON or LESOTHO or LIBERIA or LIBYA or MACEDONIA or MADAGASCAR or MALAWI or MALAYSIA or MALDIVES or MALI or “MARSHALL ISLANDS” or MAURITANIA or MAURITIUS or MEXICO or MICRONESIA or MOLDOVA or MONGOLIA or MONTENEGRO or MOROCCO or MOZAMBIQUE or MYANMAR or NAMIBIA or NEPAL or NICARAGUA or NIGER or NIGERIA or PAKISTAN or PANAMA or “PAPUA NEW GUINEA” or PARAGUAY or PERU or PHILIPPINES or ROMANIA or RUSSIA* or RWANDA or SAMOA or “SAO TOME AND PRINCIPE” or SENEGAL or SERBIA or “SIERRA LEONE” or “SOLOMON ISLANDS” or SOMALIA or “SOUTH AFRICA” or “SOUTH SUDAN” or “SRI LANKA” or “ST. LUCIA” or “ST. VINCENT AND THE GRENADINES” or SUDAN or SURINAME or SWAZILAND or “SYRIAN ARAB REPUBLIC” or TAJIKISTAN or TANZANIA or THAILAND or “TIMOR-LESTE” or TOGO or TONGA or TUNISIA or TURKEY or TURKMENISTAN or TUVALU or UGANDA or UKRAINE or UZBEKISTAN or VANUATU or VENEZUELA or VIETNAM or “WEST BANK AND GAZA” or YEMEN or ZAMBIA or ZIMBABWE)
Search 3 - relating to health<br>TS=(health* or medical or hospital or clinic* or treatment) OR AD=(health or hlth)
Search 4 - relating to decision/policy making<br>TS=(polic* or decision-mak* or decision-support or decision-process or decision-aid* or implement* or impact) or AD=(policy)
Search 5 - relating to NCD<br>TS=(NCD* or “noncommunicable disease” or “non-communicable disease” or “chronic disease” or “chronic illness” or “cardiovascular disease” or stroke or “heart attack” or cancer* or “respiratory disease*” or “chronic obstructive pulmonary disease” or asthma or diabetes or alcohol or drink* or smok* or tobacco or “physical inactivity” or “unhealthy diet” or obesity or “mental health” or “hypertension” or “depression” or Neoplasm* or “Musculoskeletal disease” or “Digestive system disease” or “Stomatognathic disease” or “Respiratory tract disease” or “Otorhinolaryngologic disease” or “Nervous system disease” or “Eye disease” or “Male urogenital disease” or “Female urogenital disease” or “pregnancy complications” or “Hemic and lymphatic disease” or “Congenital disease” or “hereditary disease” or “neonatal disease” or “Congenital abnormalities” or “hereditary abnormalities” or “neonatal abnormalities” or “Skin disease” or “connective tissue disease” or “Nutritional disease” or “metabolic disease” or “Endocrine system disease” or “Immune system disease” or “Mental disorder” or "bone disease" or "joint disease" or "rheumatic disease" or "liver disease" or "pancreatic disease" or "mouth disease" or "lung disease" or "central nervous system disease" or "neuromuscular disease" or "vision disorder" or "heart disease" or "vascular disease" or "anemia" or "blood coagulation disorder" or "thyroid disease" or "autoimmune disease"

Scopus

**Keyword search**. TITLE-ABS-KEY search, where KEY includes author keywords and controlled indexed terms in searched databases. Scopus automatically searches plural versions of words, as well as US-UK spelling variations. Each category combined with "AND". Restricted to English and year 2015 or later. Search run on 10th May 2018
Model types<br>TITLE-ABS-KEY("operation* research") OR<br>TITLE-ABS-KEY(model* W/5 (mathematical or queu* or inventory or scheduling or demand or forecast* or comput* or network or stochastic or decision* or delivery or simulation or optimization or non-linear or nonlinear or linear or Markov or cost-effectiveness or agent-based)) OR<br>TITLE-ABS-KEY(optimization W/5 (mathematical or nonlinear or non-linear or linear or network or discrete or multicriteria or multi-criteria or stochastic or problem or minimization or maximization or location or allocation)) OR<br>TITLE-ABS-KEY(simulation W/3 (comput* or discrete or agent-based or system$))
Geographic focus<br>TITLE-ABS-KEY("low-resource setting*" or "resource-constrained setting*" or "resource-poor setting*" or "limited-resource setting*" or "resource-limited setting*" or Africa* or (Asia* W/2 south) or (Asia W/2 east) or "latin America*" or "central America*" or "south america*" or caribbean or "west indies" or "middle east")<br>TITLE-ABS-KEY((countr* or nation* or population* or world) W/0 (developing or "less* developed" or "under developed" or "least-developed" or underdeveloped or "middle income" or "middle-income" or "low-income" or "low* income" or underserved or "under served" or deprived or poor*))<br>TITLE-ABS-KEY(econom* W/0 (developing or "less* developed" or "under developed" or underdeveloped or "middle income" or middle-income or "low* income" or low-income))<br>TITLE-ABS-KEY(low* W/0 (gdp or gnp or "gross domestic" or "gross national"))<br>TITLE-ABS-KEY(lmic* or "third world" or "lami countr*" or "transitional countr*")<br>TITLE-ABS-KEY(*The names of all countries included in the World Bank LMIC classification 2018*)
Health<br>TITLE-ABS-KEY(health* or medical or hospital or clinic* or treatment) or AFFIL(health)
Decision-/policy-making<br>TITLE-ABS-KEY(polic* or decision-mak* or decision-support or decision-process or decision-aid* or implement* or impact) OR AFFIL(policy)
**NCDs**<br>TITLE-ABS-KEY(NCD* or "noncommunicable disease" or "non-communicable disease" or "chronic disease" or "chronic illness" or "cardiovascular disease" or stroke or "heart attack" or cancer* or "respiratory disease*" or "chronic obstructive pulmonary disease" or COPD or asthma or diabet* or alcohol or drink* or smok* or tobacco or "physical inactivity" or "unhealthy diet" or obes* or "mental health" or hypertension or depression or Neoplasm* or "Musculoskeletal disease" or "Digestive system disease" or "Stomatognathic disease" or "Respiratory tract disease" or "Otorhinolaryngologic disease" or "Nervous system disease" or "Eye disease" or "Male urogenital disease" or "Female urogenital disease" or "pregnancy complications" or "Hemic and lymphatic disease" or "Congenital disease" or "hereditary disease" or "neonatal disease" or "Congenital abnormalities" or "hereditary abnormalities" or "neonatal abnormalities" or "Skin disease" or "connective tissue disease" or "Nutritional disease" or "metabolic disease" or "Endocrine system disease" or "Immune system disease" or "Mental disorder" or "bone disease" or "joint disease" or "rheumatic disease" or "liver disease" or "pancreatic disease" or "mouth disease" or "lung disease" or "central nervous system disease" or "neuromuscular disease" or "vision disorder" or "heart disease" or "vascular disease" or "anemia" or "blood coagulation disorder" or "thyroid disease" or "autoimmune disease")

Embase

Ovid Embase – Search run on 10th May 2018

		MeSH subject headings/Custom search<br>Explode and Focus all subject headings	Hits
Model types	1	Decision support system	9712
	2	Economic model	498
	3	Statistical model	21239
	4	Monte Carlo method	5262
	5	Markov chain	419
	6	Algorithms	56280
	7	Decision theory	507
	8	Decision trees	1290

	9	Systems theory	898
	10	Computer analysis	4213
	11	Computer simulation	17550
	12	Mathematical computing	3233
	13	Systems analysis	5559
	14	Probability theory	2735
	15	Cost benefit analysis	10049
	16	Health services research	9525
	17	((operation* adj1 research) OR<br>(model* adj5 (mathematical or queu* or inventory or scheduling or demand or forecast* or comput* or network or stochastic or decision* or delivery or simulation or optimi#ation or linear or nonlinear or non-linear or Markov or cost-effectiveness or agent-based)) OR<br>(optimi#ation$1 adj5 (mathematical or nonlinear or non-linear or linear or network or discrete or multi-criteria or multicriteria or stochastic or problem or minimi#ation or maximi#ation or location or allocation)) OR<br>(simulation adj3 (comput* or discrete or agent-based or system$1))).ti,ab.	329370
	18	OR (1 to 17)	448673
Geographic focus	25	("low-resource setting*" or "resource-constrained setting*" or "resource-poor setting*" or "limited-resource setting*" or "resource-limited setting*" or Africa* or (Asia* adj2 south) or (Asia adj2 east) or "latin America*" or "central America*" or "south america*" or caribbean or "west indies" or "middle east").ti,ab.	324864
	26	((countr* or nation* or population* or world) adj0 (developing or "less* developed" or "under developed" or "least-developed" or underdeveloped or "middle income" or "middle-income" or "low-income" or "low* income" or underserved or "under served" or deprived or poor*)).ti,ab.	945
	27	(econom* adj0 (developing or "less* developed" or "under developed" or underdeveloped or "middle income" or middle-income or "low* income" or low-income)).ti,ab.	221
	28	(low* adj0 (gdp or gnp or "gross domestic" or "gross national").ti,ab.	311
	29	(lmic* or third world or lami countr* or transitional countr*).ti,ab.	6795
	30	(AFGHANISTAN or ALBANIA or ALGERIA or "AMERICAN SAMOA" or ANGOLA or ARGENTINA or ARMENIA or ARMENIAN or AZERBAIJAN or BANGLADESH or BELARUS or BYELARUS or BYELORUSSIAN or BELORUSSIA* or BELIZE or BENIN or BHUTAN or BOLIVIA or BOSNIA or "BOSNIA AND HERZEGOVINA" or HERZEGOVINA or HERCEGOVINA or BOTSWANA or BRASIL or BRAZIL or BULGARIA or "BURKINA FAS#O" or BURUNDI or URINDI or CAMBODIA or "KHMER REPUBLIC" or "KAMPUCHEA or CAMERO* or "CAPE VERDE" OR "CABO VERDE" or "CENTRAL AFRICAN REPUBLIC" or CHAD or CHINA or COLOMBIA or COMOR* or "COMORO ISLANDS" or MAYOTTE or CONGO or ZAIRE or "COSTA RICA" or "COTE D'IVOIRE" or "IVORY COAST" or CUBA or DJIBOUTI or "FRENCH SOMALILAND" or DOMINICA* or ECUADOR or EGYPT or "UNITED ARAB REPUBLIC" or "EL SALVADOR" or "EQUATORIAL GUINEA" or ERITREA or ETHIOPIA or FIJI or GABON* or GAMBIA or GEORGIA* or GHANA or GRENADA or GUATEMALA or GUINEA or GUAM or GUINEA-BISSAU or GUIANA or GUYANA or HAITI or HONDURAS or INDIA or MALDIVES or INDONESIA or IRAN<br><br>or IRAQ or JAMAICA or JORDAN or KAZAKH* or KENYA or KIRIBATI or KOREA or KOSOVO or KYRGYZ* or KIRGHIZ* or KIRGIZSTAN or LAO* or LEBANON or LESOTHO or BASUTOLAND or LIBERIA or LIBYA or MACEDONIA or MADAGASCAR or MALAGASY or MALAWI or MALAY* or SABAH or SARAWAK or MALDIVES or MALI or "MARSHALL ISLANDS" or MAURITANIA or MAURITIUS or "AGALEGA ISLANDS" or MEXICO or MICRONESIA or "MIDDLE EAST" or MOLDOV* or MONGOLIA or MONTENEGRO or MOROCCO or IFNI or MOZAMBIQUE or MYANMA* or BURMA or NAMIBIA or NEPAL or "NETHERLANDS ANTILLES" or NICARAGUA or NIGER*<br><br>or "NORTHERN MARIANA ISLANDS" or PAKISTAN or PANAMA or "PAPUA NEW GUINEA" or PARAGUAY or PERU or PHI#LIP#INES or ROMANIA or RUMANIA or ROUMANIA or RUSSIA* or R#ANDA or SAMOA* or "NAVIGATOR ISLAND*" or "SAO TOME" or SENEGAL or SERBIA or MONTENEGRO or "SIERRA LEONE" or "SOLOMON	1397566

		ISLANDS" or SOMALIA or "SOUTH AFRICA" or "SRI LANKA" or "S* LUCIA" or "ST. VINCENT AND THE GRENADINES" or SUDAN or SURINAM* or SWAZILAND or SYRIA* or TAJIKISTAN or TADZHIK* or TADJIKISTAN or TANZANIA or THAILAND or TIMOR-LESTE or TOGO* or TONGA or TUNISIA or TURKEY or TURKMEN* or TUVALU or UGANDA or UKRAINE or UZBEK* or VANUATU or VENEZUELA or VIETNAM or "VIET NAM" or "WEST BANK" or YEMEN or ZAMBIA or ZIMBABWE or RHODESIA)**.ti,ab.**	
	31	OR (24 to 30)	1600851
Decision/Policy making	32	(polic* or decision-mak* or "decision making" or decision-support or decision-process or decision-aid* or implement* or impact).ti,ab.	1856619
Noncommunicable diseases	35	((NCD* or "noncommunicable disease" or "non-communicable disease" or "chronic disease" or "chronic illness" or "cardiovascular disease" or stroke or "heart attack" or cancer* or "respiratory disease*" or "chronic obstructive pulmonary disease" or COPD or asthma or diabet* or alcohol or drink* or smok* or tobacco or "physical inactivity" or "unhealthy diet" or obes* or "mental health" or "hypertension" or "depression") or (neoplasm* or "musculoskeletal disease" or "digestive system disease" or "stomatognathic disease" or "respiratory tract diseases" or "Otorhinolaryngologic disease" or "Nervous system disease" or "Eye disease" or "Male urogenital disease" or "Female urogenital disease" or "pregnancy complications" or "Hemic and lymphatic disease" or "Congenital disease" or "hereditary disease" or "neonatal disease" or "Congenital abnormalities" or "hereditary abnormalities" or "neonatal abnormalities" or "Skin disease" or "Connective tissue disease" or "Nutritional disease" or "metabolic disease" or "Endocrine system disease" or "Immune system disease" or "Mental disorder") or ("bone disease" or "joint disease" or "rheumatic disease" or "liver disease" or "pancreatic disease" or "mouth disease" or "lung disease" or "central nervous system disease" or "neuromuscular disease" or "vision disorder" or "heart disease" or "vascular disease" or "anemia" or "blood coagulation disorder" or "thyroid disease" or "autoimmune disease")).ti,ab.	5575472
	36	33 OR 34 OR 35	12157808
Relating to health	37	(health* or medical or hospital or clinic* or treatment).ti,ab.	11375649
	38	23 AND 31 AND 32 AND 36 AND 37	2601
	39	Limit 38 to English and 2015-18	1085

<u>Medline</u>

**Ovid MEDLINE** – Search run on 10th May 2018			
		**MeSH subject headings/Custom search Explode and Focus all subject headings**	**Hits**
Model types	1	Decision support techniques	22987
	2	Models, economic	4456
	3	Models, econometric	1296
	4	Monte Carlo method	4021
	5	Markov chains	2061
	6	Stochastic processes	3864
	7	Algorithms	110470
	8	Decision theory	2073
	9	Decision trees	1660
	10	Operations research	10659
	11	Systems theory	1200
	12	Computing methodologies	386807
	13	Computer simulation	45396
	14	Mathematical computing	2815
	15	Numerical analysis, computer-assisted	1340
	16	Systems analysis	18350
	17	Probability theory	172
	18	Programming, linear	202
	19	Cost-benefit analysis	6877
	20	Health services research	48446
	21	Decision-making, organizational	3720
	22	((operation* adj1 research) OR (model* adj5 (mathematical or queu* or inventory or scheduling or demand or forecast* or comput* or network or stochastic or decision* or delivery or	267554

		simulation or optimi#ation or linear or nonlinear or non-linear or Markov or cost-effectiveness or agent-based)) OR<br>(optimi#ation$1 adj5 (mathematical or nonlinear or non-linear or linear or network or discrete or multi-criteria or multicriteria or stochastic or problem or minimi#ation or maximi#ation or location or allocation)) OR<br>(simulation adj3 (comput* or discrete or agent-based or system$1))).ti,ab.	
	23	OR (1 to 22)	715031
Geographic focus	24	Following MeSH terms exploded and focused:<br>Africa; (From “Caribbean Region”) Cuba, Dominica, Dominican Republic, Grenada, Haiti, Jamaica, Saint Lucia, Saint Vincent and the Grenadines; Central America; (From “North America”) Mexico;<br>South America; Asia, Central; (From “Asia, Northern”) Russia; (From “Asia, Southeastern”) Cambodia, Timor-Leste, Indonesia, Laos, Malaysia, Myanmar, Philippines, Thailand, Vietnam;<br>(From “Asia, Western”) Bangladesh, Bhutan, India, Nepal, Pakistan, Sri Lanka; (From “Middle East”) Afghanistan, Iran, Iraq, Jordan, Lebanon, Syria, Turkey, Yemen;<br>(From “Far East”) China, Mongolia;<br>(From “Europe, Eastern”) Albania, Bosnia and Herzegovina, Bulgaria, Kosovo, Macedonia, Moldova, Montenegro, Belarus, Romania, Russia, Serbia, Ukraine; Transcaucasia; (From “Islands/Pacific Islands/Melanesia”) Fiji, Papua New Guinea, Vanuatu; (From “Polynesia”) Samoa, Tonga	64
	25	(“low-resource setting*” or “resource-constrained setting*” or “resource-poor setting*” or “limited-resource setting*” or “resource-limited setting*” or Africa* or (Asia* adj2 south) or (Asia adj2 east) or “latin America*” or “central America*” or “south america*” or caribbean or “west indies” or “middle east”).ti,ab.	260978
	26	((countr* or nation* or population* or world) adj0 (developing or “less* developed” or “under developed” or “least-developed” or underdeveloped or “middle income” or “middle-income” or “low-income” or “low* income” or underserved or “under served” or deprived or poor*)).ti,ab.	712
	27	(econom* adj0 (developing or “less* developed” or “under developed” or underdeveloped or “middle income” or middle-income or “low* income” or low-income)).ti,ab.	185
	28	(low* adj0 (gdp or gnp or “gross domestic” or “gross national”).ti,ab.	214
	29	(lmic* or third world or lami countr* or transitional countr*).ti,ab.	5637
	30	(AFGHANISTAN or ALBANIA or ALGERIA or “AMERICAN SAMOA” or ANGOLA or ARGENTINA or ARMENIA or ARMENIAN or AZERBAIJAN or BANGLADESH or BELARUS or BYELARUS or BYELORUSSIAN or BELORUSSIA* or BELIZE or BENIN or BHUTAN or BOLIVIA or BOSNIA or “BOSNIA AND HERZEGOVINA” or HERZEGOVINA or HERCEGOVINA or BOTSWANA or BRASIL or BRAZIL or BULGARIA or “BURKINA FAS#O” or BURUNDI or URINDI or CAMBODIA or “KHMER REPUBLIC” or “KAMPUCHEA or CAMERO* or “CAPE VERDE” OR “CABO VERDE” or “CENTRAL AFRICAN REPUBLIC” or CHAD or CHINA or COLOMBIA or COMOR* or “COMORO ISLANDS” or MAYOTTE or CONGO or ZAIRE or “COSTA RICA” or “COTE D'IVOIRE” or “IVORY COAST” or CUBA or DJIBOUTI or “FRENCH SOMALILAND” or DOMINICA* or ECUADOR or EGYPT or ”UNITED ARAB REPUBLIC” or “EL SALVADOR” or “EQUATORIAL GUINEA” or ERITREA or ETHIOPIA or FIJI or GABON* or GAMBIA or GEORGIA* or GHANA or GRENADA or GUATEMALA or GUINEA or GUAM or GUINEA-BISSAU or GUIANA or GUYANA or HAITI or HONDURAS or INDIA or MALDIVES or INDONESIA or IRAN<br><br>or IRAQ or JAMAICA or JORDAN or KAZAKH* or KENYA or KIRIBATI or KOREA or KOSOVO or KYRGYZ* or KIRGHIZ* or KIRGIZSTAN or LAO* or LEBANON or LESOTHO or BASUTOLAND or LIBERIA or LIBYA or MACEDONIA or MADAGASCAR or MALAGASY or MALAWI or MALAY* or SABAH or SARAWAK or MALDIVES or MALI or “MARSHALL ISLANDS” or MAURITANIA or MAURITIUS or “AGALEGA ISLANDS” or MEXICO or MICRONESIA or “MIDDLE EAST” or MOLDOV* or MONGOLIA or MONTENEGRO or MOROCCO or IFNI or MOZAMBIQUE or MYANMA* or BURMA or NAMIBIA or NEPAL or “NETHERLANDS ANTILLES” or NICARAGUA or NIGER*	1063597

		or "NORTHERN MARIANA ISLANDS" or PAKISTAN or PANAMA or "PAPUA NEW GUINEA" or PARAGUAY or PERU or PHI#LIP#INES or ROMANIA or RUMANIA or ROUMANIA or RUSSIA* or R#ANDA or SAMOA* or "NAVIGATOR ISLAND*" or "SAO TOME" or SENEGAL or SERBIA or MONTENEGRO or "SIERRA LEONE" or "SOLOMON ISLANDS" or SOMALIA or "SOUTH AFRICA" or "SRI LANKA" or "S* LUCIA" or "ST. VINCENT AND THE GRENADINES" or SUDAN or SURINAM* or SWAZILAND or SYRIA* or TAJIKISTAN or TADZHIK* or TADJIKISTAN or TANZANIA or THAILAND or TIMOR-LESTE or TOGO* or TONGA or TUNISIA or TURKEY or TURKMEN* or TUVALU or UGANDA or UKRAINE or UZBEK* or VANUATU or VENEZUELA or VIETNAM or "VIET NAM" or "WEST BANK" or YEMEN or ZAMBIA or ZIMBABWE or RHODESIA)**.ti,ab.**	
	31	OR(24 to 30)	1068196
Decision/Policy making	32	(polic* or decision-mak* or "decision making" or decision-support or decision-process or decision-aid* or implement* or impact).ti,ab.	1383086
Noncommunicable diseases	33	Following MeSH terms exploded and focused:<br>Neoplasms; Musculoskeletal diseases; Digestive system diseases; Stomatognathic diseases; Respiratory tract diseases; Otorhinolaryngologic diseases; Nervous system diseases; Eye diseases; Male urogenital diseases; Female urogenital diseases and pregnancy complications; Cardiovascular diseases; Hemic and lymphatic diseases; Congenital, hereditary, and neonatal diseases and abnormalities; Skin and connective tissue diseases; Nutritional and metabolic diseases; Endocrine system diseases; Immune system diseases; Mental Disorders	10884546
	34	Mesh Term: noncommunicable diseases	167
	35	((NCD* or "noncommunicable disease" or "non-communicable disease" or "chronic disease" or "chronic illness" or "cardiovascular disease" or stroke or "heart attack" or cancer* or "respiratory disease*" or "chronic obstructive pulmonary disease" or COPD or asthma or diabet* or alcohol or drink* or smok* or tobacco or "physical inactivity" or "unhealthy diet" or obes* or "mental health" or "hypertension" or "depression") or (neoplasm* or "musculoskeletal disease" or "digestive system disease" or "stomatognathic disease" or "respiratory tract diseases" or "Otorhinolaryngologic disease" or "Nervous system disease" or "Eye disease" or "Male urogenital disease" or "Female urogenital disease" or "pregnancy complications" or "Hemic and lymphatic disease" or "Congenital disease" or "hereditary disease" or "neonatal disease" or "Congenital abnormalities" or "hereditary abnormalities" or "neonatal abnormalities" or "Skin disease" or "Connective tissue disease" or "Nutritional disease" or "metabolic disease" or "Endocrine system disease" or "Immune system disease" or "Mental disorder") or ("bone disease" or "joint disease" or "rheumatic disease" or "liver disease" or "pancreatic disease" or "mouth disease" or "lung disease" or "central nervous system disease" or "neuromuscular disease" or "vision disorder" or "heart disease" or "vascular disease" or "anemia" or "blood coagulation disorder" or "thyroid disease" or "autoimmune disease")).ti,ab.	4027990
	36	33 OR 34 OR 35	12157808
Relating to health	37	(health* or medical or hospital or clinic* or treatment).ti,ab.	8571581
	38	23 AND 31 AND 32 AND 36 AND 37	2601
	39	Limit 38 to English and 2015-18	1123

* Income level as specified by World Bank in 2018 https://datahelpdesk.worldbank.org/knowledgebase/articles/906519-world-bank-country-and-lending-groups; LI: low-income economy; LMI: lower-middle-income economy; UMI: upper-middle-income economy; HI: high-income economy
BOD: burden of disease; BMI: body mass index; CEA: cost-effectiveness analysis; CI: confidence interval; COI: cost-of-illness; CUA: cost-utility analysis; CVD: cardiovascular disease; DALY: disability-adjusted life year; ICER: incremental cost-effectiveness ratio; MI: myocardial infarction; QALY: quality-adjusted life year; SES: socioeconomic status; UI: uncertainty interval; WTP: willingness to pay
