# Supplementary material for: Operational research to support equitable non-communicable disease policy in low-income and middle-income countries in the sustainable development era: a scoping review
Source: BMJ Glob Health. 2020 Jun 30;5(6):e002259. doi: 10.1136/bmjgh-2019-002259 (PMC7328817; doi:10.1136/bmjgh-2019-002259)
Supplement: Supplementary data [file bmjgh-2019-002259supp002.pdf]

Contents

Appendix 2 1
Table A: Summary table of included studies 1
Table B: Expanded table of included studies 2
Table C: Intersection between application area and equity approach 30
References 31

Appendix 2

Table A: Summary table of included studies

Risk factor / Disease area	Public Health	Population Screening	Access	Comparative
Air pollution	4 [1-4]			
Air pollution & Low physical activity	1 [5]			
Alcohol use	1 [6]			
Child and maternal malnutrition	3 [7-9]			
Dietary risks	8 [10-17]			
Other environmental risks	1 [18]			
Tobacco	8 [19-26]			
Tobacco, Alcohol use, Dietary risks	1 [27]			
Tobacco, Dietary risks	2 [28, 29]			
Cardiovascular diseases		2 [30,31]	6 [60-65]	9 [101-109]
Chronic respiratory			1 [66]	3 [110-112]
Diabetes and kidney diseases		3 [32-34]	7 [67-73]	9 [113-121]
Digestive diseases		1 [35]		1 [122]
Mental disorders			6 [74-79]	1 [123]
Musculoskeletal disorders		1 [36]	1 [80]	4 [124-127]
Neoplasms		17 [37-53]	10 [81-90]	21 [128-148]
Other non-communicable diseases		2 [54, 55]	5 [91-95]	1 [149]
Sense organ diseases		4 [56-59]	2 [96, 97]	
Skin disease			1 [98]	
Substance use			2 [99, 100]	

Table B: Expanded table of included studies

Reference	Country/Region (Income level)	Policy/Intervention and Comparator	Equity Consideration	Evaluation Results
**Public Health Policy – Air pollution**				
Huang et al. (2017) [1]	China (UMI)	**Intervention:** Different air pollution control scenarios: (i) Beijing Olympics level (mean PM2.5 of 55) by 2030; (ii) China Class II standard (mean PM2.5 of 35) by 2030; (iii) WHO standard (mean PM2.5 of 10) by 2030<br>**Comparator:** No air pollution control (mean PM2.5 of 61)	None	• Strategy (i) of reduction to Beijing Olympics level would gain 241,000 life-years annually relative to no pollution control<br>• Strategy (ii) of reduction to China Class II standard would gain 992,000 life-years annually relative to no pollution control<br>• Strategy (iii) of reduction to WHO standard would gain 1,827,000 life-years annually relative to no pollution control<br>• In comparison, WHO goal of 25% improvement in systolic hypertension control combined with 30% reduction in smoking would jointly gain 928,000 life-years
Li et al. (2015) [2]	China (UMI)	**Intervention:** Municipal solid waste management (MSWM) strategies: (i) landfill deposition only; (ii) landfill and waste-to-energy (WTE) incineration; (iii) landfill + biological compost + material recovery facility (MRF)<br>**Comparator:** Comparison between strategies	None	• Strategy (iii) (landfill + compost + MRF) has least negative health impact than other scenarios<br>• Generally, adverse health effects are negligible under all scenarios
Madaniyazi et al. (2015) [3]	China (UMI)	**Intervention:** Maximum feasible reduction of air particulate matter: annual mean PM2.5 concentration to fall by 20.41mg/m^3<br>**Comparator:** Current legislation scenario: annual mean PM2.5 concentration to fall by 0.62mg/m^3	None	• Current legislation scenario would cause up to 124,000 additional deaths when population growth is considered; by contrast maximum feasible reduction scenario could avoid 230,000 deaths even after accounting for population growth<br>• Result strongly affected by rate of population growth
Xie et al. (2016) [4]	China (UMI)	**Intervention:** Cooperative reduction model (CRM) to reduce SO_2 emission: includes long-term incentives for provinces to cooperate in minimising pollutants and removal cost<br>**Comparator:** Non-cooperative reduction model (NCRM): each province acts alone	None	• CRM resulted in 12.1% reduction in mortality and 1.9% reduction in total cost relative to NCRM.<br>• The aged group (65 years and over) benefits most from policy.<br>• CVD mortality fell much more than respiratory disease mortality under both CRM and NCRM
**Public Health Policy – Air pollution, low physical activity**				
Sa et al. (2017) [5]	Brazil (UMI)	**Intervention:** Travel pattern scenarios: (i) citywide adoption of the pattern of those living in city's expanded centre; (ii) London in 2012; (iii) highly motorised like California; (iv) visionary "SP 2040" – high levels of walking and cycling<br>**Comparator:** Actual travel patterns in Sao Paulo in 2012	None	• The SP 2040 scenario averted 63,600 DALYs, with 4.7% of premature deaths from ischaemic heart disease avoided from increases in physical activity alone<br>• Highly motorised (California) scenario resulted additional 54,900 DALYs, including increases in road traffic deaths and pedestrian injuries<br>• Parameters related to air pollution had the largest impact on result uncertainty

**Public Health Policy – Alcohol use**				
Mushayabasa (2015) [6]	South Africa (UMI)	**Policy:** Alcohol control policies: (i) reducing the level of interaction between light and heavy drinkers (e.g., through education effort); (ii) increasing the proportion of treated individuals who permanently quit alcohol; (iii) reducing proportion of light drinkers who fail to permanently quit drinking<br>**Comparator:** No policy	None	• The policy to reduce the level of social interaction between light and heavy drinkers was the most effective out of three policies in reducing alcohol consumption level
**Public Health Policy – Child and maternal malnutrition**				
Botteman and Detzel (2015) [7]	Philippines (LMI), Malaysia (UMI) and Singapore	**Policy:** Partially hydrolysed whey-based formula (PHF-W) to reduce atopic dermatitis (AD) symptoms<br>**Comparator:** Standard cow milk formula (CMF)	Disadvantaged group: neonates	• PHF-W reduced absolute risk of AD incidence by 14% relative to CMF by 6 years of age<br>• Depending on country, PHF-W reduced number of days with AD symptoms by 16 to 38 days and increased discounted QALYs by 0.02 to 0.04<br>• PHF-W generated cost savings in all three countries compared to CMF
Dainelli et al. (2017) [8]	China (UMI)	**Policy:** Increased potassium intake (>700mg/day) through potassium-fortified milk powder for adults aged 50-79 who regularly drink milk<br>**Comparator:** No potassium fortification	None	• Potassium fortification intervention cost-effective at 3-times GDP per capita WTP threshold
Vosti et al. (2015) [9]	Cameroon (LMI)	**Policy:** Compositional and geographical changes to Vitamin A (VA) programme for children at subnational region level<br>**Comparator:** Status quo: temporally and spatially uniform composition	Disadvantaged group: children	• VA programme most cost-effective in the North macro-region, where needs are greatest and the programme cost per child effectively covered is lowest<br>• Optimised policy is cost-saving relative to status quo: 44% less expensive with no change in the total number of children effectively covered nationwide (n=12.9 million over 10 years)
**Public Health Policy – Dietary risks**				
Barrientos-Gutierrez et al. (2017) [10]	Mexico (UMI)	**Policy:** (i) actual 1 peso-per-litre excise tax on all non-alcoholic sugar sweetened beverages (SSBs); (ii) hypothetical 2 peso-per-litre tax<br>**Comparator:** No tax	Differential subgroup impact	• Actual 1 peso-per-litre tax projected to reduce BMI by $0.15kg/m^2$ on average, decrease obesity incidence by 2.54% and avoid 92,000 T2 diabetes cases by 2030 and 240,000 by 2050 relative to no tax<br>• Largest obesity and diabetes reductions would occur in low SES groups and younger cohort aged 20-35 at baseline<br>• Hypothetical 2 peso-per-litre tax would produce twice as large impact
Erkoyun et al. (2016) [11]	Turkey (UMI)	**Policy:** Hypothetical policy that: (i) reduces daily salt intake gradually by 0.47g per year from 2013 to 2025; (ii) meets WHO	None	• Gradual policy would reduce prevalence of ischaemic heart disease (IHD) and stroke by 0.3% and 0.2% respectively relative to no policy by 2025

		recommendation of daily salt intake of 5g by 2025<br>**Comparator:** No change in salt intake		• WHO policy would reduce prevalence of IHD and stroke by 0.8% and 0.5% respectively relative to no policy by 2025
Marsh et al. (2016) [12]	Mexico (UMI)	**Policy:** Consumption of lower protein infant formula from infanthood to reduce risk of childhood obesity<br>**Comparator:** Consumption of current infant formulas	Disadvantaged group: infants	• Infants who consumed lower protein formula were 10.5% less likely to be obese which translated to 0.01 QALYs gained on average; they also incurred less societal cost (984 Mexican pesos per individual) due to lower disease burden
Sanchez-Romero et al. (2016) [13]	Mexico (UMI)	**Policy:** Hypothetical tax on SSBs that produces: (i) 10% reduction in SSB consumption; (ii) 20% reduction in SSB consumption<br>**Comparator:** No tax	None	• 10% reduction in SSB consumption with 39% calorie compensation would result in 189,300 fewer incidences of T2 diabetes, 20,400 fewer incidences of stroke and MI and 18,900 fewer deaths between 2013 and 2022. This would save Int$983 million from healthcare savings<br>• Largest reduction in disease burden and healthcare cost would occur among young adults aged 35-44 at baseline
Wang et al. (2016) [14]	China (UMI)	**Policy:** Population-wide salt restriction policies: (i) gradual reduction over 10 years of daily salt consumption to 9g/day; (ii) gradual reduction to 7.5g/day; (iii) gradual reduction to 6g/day; (iv) promote salt-restriction spoons; (v) promote salt substitutes<br>**Comparator:** No change in salt intake over 10 years	None	• Policy (i) would prevent 197,000 CVD events, reduce CVD mortality by 2.5%, gain 303,000 QALYs and save $1.4 billion in annual CVD costs<br>• Policy (ii) would double the benefits of strategy (i)<br>• Strategy (iv) would prevent 183,000 CVD events and save $1.4 billion in annual CVD costs<br>• Strategy (v) would generate three times the benefits of strategy (iv)
Watkins et al. (2016b) [15]	South Africa (UMI)	**Policy:** Combined policy to reduce daily salt consumption to 5g including informational campaign and mandatory regulation of commercially produced foods in six key groups<br>**Comparator:** No policy	Differential subgroup impact	• Policy would reduce annual CVD mortality by 11% with similar health gains across income quintiles<br>• Policy would save $4.06 million in OOP expenditure and $51.25 million in public healthcare subsidies. Policy costs $0.01 per capita and is hence cost-saving<br>• Policy could avert 2,400 cases of catastrophic health expenditures and 2,000 cases of poverty per year
Webb et al. (2017) [16]	Global (LI/LMI/UMI)	**Policy:** "Soft regulation" policy combining industry agreements, government monitoring, and public education to reduce salt intake; reaching full scale in 10th year; different programme efficacy by country; optimal salt intake target of 2.0g/day<br>**Comparator:** No policy	None	• Weighted average ICER of Int$146 per DALY averted in UMI countries<br>• Weighted average ICER of Int$111 per DALY averted in LMI countries<br>• Weighted average ICER of Int$215 per DALY averted in LI countries
Wilcox et al. (2015) [17]	Syria (LI)	**Policy:** Salt restriction policies: (i) health promotion campaign about salt reduction; (ii) labelling of salt content on packaged foods; (iii) reformulation of salt content within packaged foods; (iv) combinations of policies	None	• All individual policies are cost-saving or cost-effective (ICER less than $13,000 per life-year gained)<br>• Combination of strategies (i), (ii) and (iii) is cost saving

		**Comparator:** No policy		
**Public Health Policy – Other environmental risks**				
Springmann et al. (2016) [18]	Global (LI/LMI/UMI)	**Intervention:** Climate stabilisation pathways conceptualised by IPCC and encompassing emission pathways and socioeconomic development pathways: (i) High emission pathway – increase in global mean surface air temperature of 2.0 degrees Celsius from 2015 to 2050; (ii) Medium emission pathway A – increase in temperature of 1.4 degrees Celsius; (iii) Medium emission pathway B – increase in temperature of 1.3 degrees Celsius; (iv) Low emission pathway – increase in temperature of 1.0 degrees Celsius<br>**Comparator:** (i) No climate change; (ii) Comparison between emission pathways	None	• Under 'middle-of-the-road' socioeconomic development pathway, high emission pathway will result in 529,000 additional deaths due to dietary and weight-related factors by 2050 relative to no climate change scenario<br>• Low emission pathway will result in 154,000 additional deaths relative to no climate change scenario<br>• Twice as many climate-related deaths were associated with reductions in fruit and vegetable consumption than with climate-related increases in the prevalence of underweight<br>• Most climate-related deaths were projected to occur in south and east Asia
**Public Health Policy – Tobacco**				
Borracci and Mulassi (2015) [19]	Argentina (UMI)	**Policy:** General policy to reduce contact with cigarettes during adolescence (aged 14-17) by 50% and 100%<br>**Comparator:** No policy	None	• Reducing contact with cigarettes during adolescence (aged 14-17) by 50% may reduce adult tobacco use rates by 7.2%; 100% reduction in contact by 16.2%
Fleischer et al. (2017) [20]	Mexico (UMI)	**Policy:** Six tobacco control policies (WHO FCTC provision): (i) excise taxes; (ii) smoke-free laws; (iii) anti-smoking public education; (iv) marketing restrictions; (v) access to tobacco cessation treatments; (vi) enforcement of ban on tobacco sales to youth<br>**Comparator:** Status quo tobacco control	None	• Excise tax was the most effective control strategy. If excise taxes were increased to 70% of the retail price from 55%, smoking prevalence would decline by 12% by 2023 and 16% by 2053 relative to 2013 status quo level. This would prevent 118,000 premature tobacco-attributable deaths by 2053<br>• Under combined tobacco control policies smoking prevalence would decline by 41% by 2023 and 50% by 2053 relative to 2013 status quo level. This would prevent 471,000 premature tobacco-attributable deaths by 2053
Levy et al. (2016) [21]	Egypt (LMI), Lebanon (UMI), Pakistan (LMI) and Tunisia (LMI)	**Policy:** Six tobacco control policies (WHO FCTC provision): (i) excise taxes; (ii) smoke-free area laws; (iii) mass media campaigns; (iv) marketing restrictions; (v) access to tobacco cessation treatments; (vi) warnings on cigarette packages<br>**Comparator:** Current status quo control policies	None	• The most effective tobacco control policy that averted most tobacco-attributable deaths over 40 years was: smoke-free area laws in Egypt (378,000 deaths); cessation treatment in Lebanon (24,200); excise taxes in Pakistan (2,533,300); and smoke-free area laws in Tunisia (80,150)<br>• Combined policies would avert 973,900 tobacco-attributable deaths in Egypt, 156,300 in Lebanon, 3,515,200 in Pakistan and 245,300 in Tunisia over 40 years
Levy et al. (2016) [22]	China (UMI)	**Policy:** Six tobacco control policies (WHO FCTC provision): (i) excise taxes; (ii) smoke-free laws; (iii) anti-smoking public education; (iv) marketing restrictions; (v) access to	None	• Excise tax was the most effective control strategy. Excise tax at 75% of retail price would reduce smoking prevalence by 12.9% between 2013 and 2050 relative to status quo and prevent 133,815 tobacco-attributable deaths

		tobacco cessation treatments; (vi) enforcement of ban on tobacco sales to youth<br>**Comparator:** Current status quo control policies		• Combined tobacco policies would reduce smoking prevalence by 40% between 2013 and 2050 and prevent 12.8 million tobacco-attributable deaths and loss of 154 million life years
Ngalesoni et al. (2017) [23]	Tanzania (LI)	**Policy:** Five tobacco control policies (WHO FCTC provision): (i) advertisement, promotion and sponsorship bans; (ii) package labelling of tobacco products; (iii) smoke-free public places; (iv) mass media campaigns; (v) increased tax on tobacco<br>**Comparator:** No policy	None	• All five policies were very cost-effective with ICERs falling below one-times GDP per capita threshold.<br>• Increased tax was most cost-effective with ICER of US$5 per DALY averted<br>• Smoke-free workplace was least cost-effective with ICER of US$267 per DALY averted
Reynales-Shigematsu et al. (2015) [24]	Mexico (UMI)	**Policy:** Seven tobacco control policies (WHO FCTC provision): (i) excise taxes; (ii) smoke-free laws; (iii) mass media campaigns; (iv) marketing restrictions; (v) access to tobacco cessation treatments; (vi) enforcement of ban on tobacco sales to youth; (vii) warning labels on packaging<br>**Comparator:** Current status quo control policies	None	• For male population, all seven policies from 2002 would reduce smoking prevalence by 30.4% relative to status quo by 2013 and by 44.2% by 2053. This would avert 9,286 tobacco-attributable deaths by 2013 and 673,340 by 2053.<br>• For female population, all seven policies from 2002 would reduce smoking prevalence by 31.1% relative to status quo by 2013 and by 46.2% by 2053. This would avert 1,508 tobacco-attributable deaths by 2013 and 152,918 by 2053.<br>• For both sexes and time horizons, excise tax on cigarettes was the most effective single strategy.
Szklo et al. (2017) [25]	Brazil (UMI)	**Policy:** Tobacco control policies implemented from 1989 to 2015: (i) 100% smoke-free laws; (ii) excise taxes<br>**Comparator:** Status quo before 1989	Disadvantaged group: women and neonates	• Policies are projected to avert 7.5 million smoking-attributable deaths among adults aged 18 and over and 0.9 million tobacco-attributable adverse maternal and child health outcomes (placenta praevia, placental abruption, pre-term birth, low birthweight, sudden infant death syndrome) by 2050
Verguet et al. (2015b) [26]	China (UMI)	**Policy:** Excise tax that increases price of tobacco by 50%, fully passed on to consumers<br>**Comparator:** No excise tax	Differential subgroup impact	• 50% increase in tobacco price would lead to 231 million life-years gained over 50 years relative to no excise tax; and a third of all life-year gains would accrue to the lowest income quintile<br>• Tax would generate US$703 billion of revenue over 50 years and reduce expenditures on tobacco-related diseases by US$24.0 billion over 50 years. 14% of the revenue would come from the lowest income quintile and 24% from the highest quintile<br>• Tax would provide financial risk protection from OOP healthcare expenditure worth US$1.8 billion over 50 years. The protection would mainly (74%) concentrated in the lowest income quintile
**Public Health Policy – Tobacco, Alcohol use, Dietary risks**				
Stacey et al. (2018) [27]	South Africa (UMI)	**Policy:** Tax policies on: (i) tobacco; (ii) beer; (iii) sugar-sweetened beverages (SSBs)<br>**Comparator:** No tax policy	None	• 60% excise tax on tobacco would gain 858,923 life-years over 30 years

				• 25% excise tax on beer would gain 568,063 life-years over 30 years<br>• 20% excise tax on SSBs would gain 688,710 life-years over 30 years
**Public Health Policy – Tobacco, Dietary risks**				
Islek et al. (2016) [28]	Turkey (UMI)	**Policy:** (i) Population-level policy scenarios (conservative, feasible and optimal) focused on dietary salt, trans fat, saturated fat, smoking and fruit and vegetable consumption; (ii) Scenarios (conservative, feasible and optimal) for improvements to primary and secondary prevention and acute care of CVD<br>**Comparator:** No policy	None	• Under optimal scenarios from 2013 to 2022: salt intake reduction could prevent 51,975 CVD deaths; trans fat 47,528; saturated fat 39,563; more fruit and vegetables 35,893; smoking 10,739; primary/secondary prevention and acute care could prevent 89,810<br>• Under conservative scenarios from 2013 to 2022: population-level policies could together prevent 58,962 CVD deaths; primary/secondary prevention and acute care 49,885
Sozmen et al. (2015) [29]	Turkey (UMI)	**Policy:** Hypothetical policies from 2010: (i) 5% reduction in obesity over 5 years; (ii) no further increase in obesity over 5 years; (iii) no further increase in obesity + 40% reduction in smoking over 15 years; (iv) 10% reduction in obesity and 20% reduction in smoking over 15 years<br>**Comparator:** No policy	None	• Strategy (i) would lead to 12.3% reduction in diabetes prevalence by 2025<br>• Similarly, strategy (ii), (iii) and (iv) would lead to 7.6%, 7.9% and 10.5% reduction, respectively, in diabetes prevalence by 2025
**Population Screening – Cardiovascular Disease**				
Dukpa et al. (2015) [30]	Bhutan (LMI)	**Intervention:** Diabetes and hypertension screening strategies for adults aged over 40: (i) opportunistic screening according to WHO guideline; (ii) universal screening through proactive public communication and invitations<br>**Comparator:** No screening	Universal healthcare coverage	• Both 70% (opportunistic) and 100% (universal) screening coverage strategies dominate (cost saving and health gain) relative to no screening<br>• Universal screening may be even more dominant (save more cost and/or gain more health) relative to no screening than 70% opportunistic screening
Gaziano et al. (2015) [31]	Guatemala (LMI), Mexico (UMI) and South Africa (UMI)	**Intervention:** CVD screening by community health workers for adults aged 35-74 without CVD history using: (i) paper-based tool; or (ii) mobile app<br>**Comparator:** Usual opportunistic screening	Universal healthcare coverage	• Strategy (ii) of mobile app can save 471 lives in South Africa, 281 lives in Mexico and 34 lives in Guatemala per 210,000 adults screened relative to usual opportunistic screening<br>• Strategy (i) of paper tool can save 336 lives in South Africa, 201 in Mexico and 24 in Guatemala per 150,000 adults screened relative to usual opportunistic screening<br>• In all countries, strategy (ii) dominates (cost saving and health gain) strategy (i)
**Population Screening – Diabetes and kidney disease**				
Basu et al. (2015b) [32]	India (LMI)	**Intervention:** Diabetes screening for adults aged 25-65 using: (i) Chaturvedi risk score; (ii)	None	• The ratio of false- to true-positives varies from 3.9 for strategy (iv) of RBG test to 8.2 for survey-based screening instruments

		Mohan risk score; (iii) Ramachandran risk score; (iv) random blood glucometer (RBG) test; (v) strategy (i) followed by (iv); (vi) strategy (ii) followed by (iv); (vii) strategy (iii) followed by (iv)<br>**Comparator:** Comparison between strategies		• Cost per diabetes case: $5.28 for strategy (iv) and $17.06 for survey instruments<br>• Total cost: $169m for strategy (iv) and $567m for survey instruments<br>• Population diabetes screening will not meet Wilson and Jungner criteria due to low sensitivity and specificity plus lack of therapeutic benefits from early detection
Teimouri et al. (2015) [33]	Iran (UMI)	**Intervention:** Alternative budget distribution between diabetes monitoring (i.e. screening) and treatment coverage rates<br>**Comparator:** 50/50 distribution between monitoring and treatment of diabetes	None	• Optimal to start with 40/60 monitoring/treatment budget distribution at start then move to 10/90 near the end of 10-year policy frame<br>• Current 50/50 distribution is sub-optimal
Toscano et al. (2015) [34]	Brazil (UMI)	**Intervention:** Universal Type 2 diabetes screening for adults aged over 40 using one-time fingerstick capillary blood glucose testing<br>**Comparator:** Opportunistic case finding only (standard practice)	Universal healthcare coverage	• Universal screening had ICER of US$31,147 per QALY relative to opportunistic case finding which was not cost-effective at threshold of 3-times GDP per capita (US$9,150 per QALY)
**Population Screening – Digestive diseases**				
Thiboonboon et al. (2015) [35]	Thailand (UMI)	**Intervention:** Expanded neonatal screening by tandem mass spectrometry (MS/MS) for PKU, IVA, MMA, PA, MSUD and MCD (inborn errors of metabolism)<br>**Comparator:** Current screening programme by Guthrie test for PKU only	(i) Disadvantaged group: neonates; (ii) Universal healthcare coverage	• Expanded screening had ICER of US$58,647 per QALY relative to current screening<br>• Early detection yielded significant health gains relative to late detection for PKU, IVA, MSUD and MCD
**Population Screening – Musculoskeletal**				
Si et al. (2015) [36]	China (UMI)	**Intervention:** Screening (dual-energy X-ray absorptiometry) and treatment (oral alendronate) for osteoporosis in postmenopausal women<br>**Comparator:** No screening or treatment	None	• Screening and treatment strategy had ICER of US$1,440 per QALY relative to no screening strategy
**Population Screening – Neoplasms (Breast cancer)**				
Haghighat et al. (2016) [37]	Iran (UMI)	**Intervention:** One, two or three rounds of mammography screening for women aged 40-70<br>**Comparator:** No screening	Disadvantaged group: women	• ICER of one round of mammography screening relative to no screening was Int$37,350 per QALY gained<br>• ICERs of second and third rounds of screening were, respectively, Int$141,641 and Int$389,148 per QALY gained relative to no screening
Nguyen and Adang (2018) [38]	Vietnam (LMI)	**Intervention:** One round of public mammography screening for women aged 45-64<br>**Comparator:** No screening	(i) Disadvantaged group: women; (ii) Universal healthcare coverage	• Relative to no screening, mammography screening had ICERs of US$8,782.70, US$3,647.06, US$4,405.44 and US$6,335.84 per life-year gained for women aged 45-59, 50–54, 55–59 and 60-64, respectively

Ulloa-Perez et al. (2016) [39]	Mexico (UMI)	**Intervention:** Mammography screening scenarios for women aged 25-75 varying by screening frequency, and coverage level<br>**Comparator:** Current screening programme	(i) Disadvantaged group: women; (ii) Universal healthcare coverage	• The 'feasible' scenario that would be cost-effective under one-time GDP per capita WTP threshold would involve reduced frequency (every three years instead of two), reduced coverage for 25-40 and 70-75 age groups, and increased coverage for all age groups between 40 and 70 relative to current screening programme. This would avert 448 less DALYs over 10 years but save $31million per year<br>• The 'objective' scenario that would be cost-effective under budget increase of $9million per year would maintain same frequency as current programme (every two years) but have same coverage levels as 'feasible' scenario. This would avert 2,403 more DALYs over 10 years
Wagh et al. (2017) [40]	Non-specific LI or LMI country (LI/LMI)	**Intervention:** Clinical breast examination for women aged 30-60 at varying screening intervals<br>**Comparator:** No screening	(i) Disadvantaged group: women; (ii) Universal healthcare coverage	• Annual screening at ages 35-39 and biennial from 41-49 would achieve a mortality reduction of 27.9% relative to no screening, while annual screening from 38-42 and triennial from 43-58 would achieve a mortality reduction of 25.5%
Zehtab et al. (2016) [41]	Iran (UMI)	**Intervention:** Mammography screening programme in rural areas for women aged 35-69<br>**Comparator:** No screening	(i) Disadvantaged group: women; (ii) Universal healthcare coverage	• ICER of US$6,264 per DALY averted for screening programme relative to no screening
**Population Screening – Neoplasms (Cervical cancer)**				
Akhavan-Tabatabaei et al. (2017) [42]	Colombia (UMI)	**Intervention:** Cervical cancer screening strategies for women aged over 25 or sexually active women aged below 25: (i) repeated Pap smear test six months after the required annual test; (ii) colposcopy without repeated smear test; (iii) do nothing after required annual test<br>**Comparator:** Comparison between strategies with variation by age and health status of participants	Disadvantaged group: women	• For most ages and health status, strategy (i) of repeated Pap smear test is recommended<br>• For risker health status and more critical age ranges (15-30 and 45-55), strategy (ii) of direct colposcopy is recommended
Campos et al. (2015) [43]	Uganda (LI)	**Intervention:** Cervical cancer screening (once in a lifetime) strategies varying by sensitivity, coverage and follow-up rate: (i) two-visit HPV DNA testing; (ii) one-visit VIA<br>**Comparator:** Comparison between strategies	(i) Disadvantaged group: women; (ii) Universal healthcare coverage	• With 10% loss to follow-up, strategy (i) of two-visit HPV DNA testing with 80–90% sensitivity was more effective and more cost-effective than strategy (ii) of one-visit VIA with 40% sensitivity.<br>• As loss to follow-up increased, strategy (i) became more costly and less effective than strategy (ii)
Campos et al. (2015b) [44]	El Salvador (LMI)	**Intervention:** Cervical cancer screening strategies for women aged 30-65: (i) HPV screening every 5 years + colposcopy; (ii) HPV screening every 5 years + cryotherapy ('screen and treat'); (iii) Pap smear test every 2 years + colposcopy	(i) Disadvantaged group: women; (ii) Universal healthcare coverage	• Strategy (ii) of 'screen and treat' had an ICER of $2,040 per year of life saved (YLS) relative to no intervention which was cost-effective at threshold of 1-time GDP per capita ($3,777 per YLS)<br>• Strategy (ii) dominated (cost saving and health gain) strategies (i) and (iii)

		**Comparator:** No intervention		
Campos et al. (2017) [45]	Uganda (LI); India (LMI); Nicaragua (LMI)	**Intervention:** Cervical cancer screening strategies for women aged 30 (3 lifetime screenings): (i) two-visit HPV DNA testing; (ii) one-visit HPV DNA testing – using new and more expensive technology<br>**Comparator:** Comparison between strategies	None	• Assuming 100% coverage and 10% loss to follow-up, strategy (ii) of one-visit HPV screening had incremental net monetary benefit of Int$13 in India, Int$36 in Nicaragua and Int$17 in Uganda relative to strategy (i) of two-visit HPV screening.<br>• Strategy (ii) was hence cost-effective relative to strategy (i) at threshold of 1-time GDP per capita (per life-year gained)
Campos et al. (2017b) [46]	Uganda (LI)	**Intervention:** Cervical cancer screening strategies for women aged over 9: (i) increase coverage of HPV DNA testing up to 90% from baseline; (ii) increase screening frequency to 2-3 times for women who already have access<br>**Comparator:** (i) No intervention; (ii) Baseline screening coverage (30% to 80% varying by region) and frequency (once in lifetime); (iii) Comparison between strategies	(i) Disadvantaged group: women; (ii) Universal healthcare coverage	• At baseline coverage of 30%, screening 3 times had an ICER of Int$540 per years of life saved (YLS) relative to no intervention which was cost-effective at threshold of 1-time GDP per capita (Int$1,690 per YLS)<br>• At baseline coverage of 30%, expanding coverage to 50% yielded comparable reduction in cancer incidence as offering three screenings to the 30% who already have access.<br>• At high baseline coverage of 70%, three screening opportunities yielded greater health benefits than increasing coverage to 90% and had ICER of Int$590 per YLS relative to no intervention
Campos et al. (2017c) [47]	Uganda (LI)	**Intervention:** HPV DNA test (once in a lifetime) self-collection (higher uptake but lower sensitivity than provider-collection) by women aged 30-49<br>**Comparator:** (i) HPV DNA test provider-collection at clinics; (ii) No screening	(i) Disadvantaged group: women; (ii) Universal healthcare coverage	• At 75% coverage, self-collection had ICER of Int$80 per year of life saved (YLS) relative to no screening and provider-collection had ICER of Int$120 per YLS which were both cost-effective at threshold of 1-time GDP per capita (Int$1,690 per YLS)<br>• When self-collection had 15-20% higher coverage, it dominated (cost savings and health gain) provider-collection
Guerrero et al. (2015) [48]	Philippines (LMI)	**Intervention:** HPV vaccination at age 11-13 (coverage rate 20% or 80%) with or without cervical screening strategies for age groups between 35 and 55: (i) visual inspection with acetic acid (VIA) test (coverage rate 8% or 80%); (ii) Pap screening (coverage rate 80%)<br>**Comparator:** (i) Current practice – Pap screening (coverage 8%; age 35-55); (ii) Comparison between combinations	(i) Disadvantaged group: women; (ii) Universal healthcare coverage	• VIA (8% coverage; age 35-55) alone, VIA (80% coverage; age 35-45) alone, VIA (80% coverage; age 35-50), and VIA (80% coverage; age 35-55) dominated (cost saving and health gain) current practice of Pap screening (8% coverage; age 35-55)<br>• The most cost-effective combined strategy was HPV vaccination (80% coverage; age 11) plus VIA (80% coverage; age 35-45) which had ICER of US$783 per QALY relative to current practice which was cost-effective at threshold of 1-time GDP per capita (US$2,835 per QALY)
Levin et al. (2015) [49]	China (UMI)	**Intervention:** Publicly financed HPV vaccination (70% coverage) before age 12 followed by cervical cancer screening with cytology and visual inspection with acetic acid (VIA) test at age 35 – lifetime screening frequency and rate of follow-up loss differed by income quintile<br>**Comparator:** Cervical cancer screening alone	(i) Differential subgroup impact; (ii) Disadvantaged group: women; (iii) Universal healthcare coverage	• Incidence of cervical cancer fell by 44% across all income quintiles<br>• Cervical cancer deaths averted and treatment-seeking cases of cancer averted were highest in the most deprived quintile<br>• Savings as a percentage of total income was 59% and highest for the most deprived quintile and 30% for the least deprived quintile

				• Vaccination policy had incremental cost of $10,920 per cervical cancer deaths averted relative to screening alone for the most deprived quintile and $13,277 for the least deprived quintile
Mo et al. (2017) [50]	China (UMI)	**Intervention:** HPV vaccination strategies (HPV2, HPV4, HPV9-valent vaccines) at age 12 followed by cervical cancer screening strategies: (i) liquid-based cytology test + HPV DNA test; (ii) Pap smear cytology test + HPV DNA test; (iii) visual inspection with acetic acid (VIA)<br>**Comparator:** (i) No screening or vaccination; (ii) Comparison between strategies	(i) Disadvantaged group: women; (ii) Universal healthcare coverage	• Screening strategy (iii) of VIA test had the lowest ICER of US$1,171.44 per QALY relative to no screening or vaccination which was cost-effective at threshold of 1-time GDP per capita (US$7,960 per QALY)<br>• Screening strategy (iii) plus HPV9-valent vaccination was the only other non-dominated strategy with ICER of US$6,065.93 per QALY relative to screening strategy (iii) alone
Setiawan et al. (2016) [51]	Indonesia (LMI)	**Intervention:** Cervical cancer prevention strategies: (i) HPV vaccination at age 12 followed by screening with visual inspection with acetic acid (VIA) test; (ii) VIA test alone<br>**Comparator:** No intervention	(i) Disadvantaged group: women; (ii) Universal healthcare coverage	• Strategy (ii) of VIA test alone had ICER of Int$3,126 per QALY relative to no intervention which was cost-effective at threshold of 1-time GDP per capita (Int$3,475 per QALY)<br>• Strategy (i) of HPV vaccination plus VIA had ICER of Int$1,863 per QALY relative to no intervention<br>• Strategy (i) had ICER of Int$1,596 per QALY relative to strategy (ii)
Termrungruang-lert et al. (2017) [52]	Thailand (UMI)	**Intervention:** Cervical cancer screening strategies for women aged 30-65: (i) HPV genotyping for HPV16/18 plus liquid-based cytology for high risk HPV (HR-HPV) positive; (ii) high-risk HPV testing alone; (iii) liquid-based cytology<br>**Comparator:** Pap cytology (usual care)	Disadvantaged group: women	• Strategy (ii) of HR-HPV testing had ICER of THB41,075 per case detected relative to Pap cytology which was cost-effective at threshold of 1-time GDP per capita<br>• Strategy (ii) dominated (cost saving and health gain) strategies (i) and (iii)
**Population Screening – Neoplasms (Lung cancer)**				
Sheehan et al. (2017) [53]	China (UMI)	**Intervention:** Strategies for lung cancer screening: (i) US Centers for Medicare & Medicaid Services (CMS) screening guideline – screen current and former smokers aged 55-77 with 30 pack-years of smoking history and less than 15 years since quitting by computerised tomography (CT); (ii) Chinese screening guideline – screen current and former smokers aged 50-74 with 20 pack-years and less than 5 years since quitting by CT<br>**Comparator:** (i) No screening; (ii) Comparison between strategies	Universal healthcare coverage	• CMS guideline would require 0.99 billion CT scans between 2016 and 2050 and reduce lung cancer mortality in males by 6.58% and in females by 1.97%<br>• Chinese guideline would require 1.43 billion CT scans between 2016 and 2050 and reduce lung cancer mortality in males by 6.30% and in females by 2.79%<br>• Chinese guideline would prevent about 20,000 (2.9%) more lung cancer deaths than CMS guideline but require 0.44 billion more CT scans
**Population Screening – Other non-communicable diseases**				

Kuznik et al. (2016) [54]	47 countries in Sub-Saharan Africa (LI/LMI/UMI)	**Intervention:** Universal neonatal screening and prophylactic intervention for sickle-cell disease<br>**Comparator:** No screening or intervention	(i) Disadvantaged group: neonates; (ii) Universal healthcare coverage	• Of the 47 countries in Sub-Saharan Africa, intervention is highly cost-effective in 24 countries (average ICER of US$184 per DALY averted) relative to no intervention, cost-effective in base case in 10 countries (average ICER of US$285 per DALY averted) but subject to uncertainty, and not cost-effective in the remaining 13
Tobe et al. (2017) [55]	China (UMI)	**Intervention:** Neonatal screening strategies for critical congenital heart disease: (i) pulse oximetry screening (POS) alone; (ii) clinical assessment alone; (iii) POS plus clinical assessment<br>**Comparator:** No screening	(i) Disadvantaged group: neonates; (ii) Universal healthcare coverage	• Strategy (ii) of clinical assessment had ICER of Int$5,728 per DALY averted relative to no screening which was cost-effective at threshold of 3-times GDP per capita (Int$34,857 per DALY averted)<br>• Strategy (ii) dominated (cost saving and health gain) strategy (i) of POS alone<br>• Strategy (iii) of POS plus clinical assessment yielded best health outcome but had ICER of Int$56,778 per DALY averted relative to strategy (ii)
**Population Screening – Sense organ diseases**				
John and Parikh (2017) [56]	India (LMI)	**Intervention:** Community glaucoma screening and treatment programme for adults aged 40-69<br>**Comparator:** Opportunistic case finding only	Universal healthcare coverage	• Screening programme had ICER Of Lak106,686 per QALY relative to opportunistic case finding which was below the threshold of Lak131,831 per QALY (Indian GDP per capita is Lak90,884)
Rothschild et al. (2016) [57]	Mexico (UMI); United States	**Intervention:** Ideal national retinopathy of prematurity (ROP) screening programme with 100% screening penetrance for infants with birthweight less than 1,500g<br>**Comparator:** Current screening programme with 52% penetrance	Universal healthcare coverage	• Ideal screening programme dominated (cost saving and health gain) current programme<br>• Incremental net monetary benefit of US$5,556 per child per year
Vinekar et al. (2017) [58]	India (LMI)	**Intervention:** Telemedicine screening for retinopathy of prematurity (ROP): implementing the Karnataka Internet Assisted Diagnosis of ROP (KIDROP) programme in other 9 nine states<br>**Comparator:** No screening	(i) Disadvantaged group: neonates and rural; (ii) Universal healthcare coverage	• Avoided financial burden was US$9.5m for Karnataka and US$108.4m for all ten states combined
Wong et al. (2017) [59]	Nicaragua (LMI)	**Intervention:** Neonatal screening strategies for congenital hearing loss with otoacoustic emissions (OAEs): (i) universal screening at all municipalities; (ii) screening only at the main regional health centre (RHC); (iii) targeted screening of high-risk infants based on main risk factors; (iv) screening at RHC plus targeted screening at other municipalities. Screening with or without treatment for hearing impairment	(i) Disadvantaged group: neonates and rural; (ii) Universal healthcare coverage	• OAE screening strategies without lifetime treatment were cost-effective with ICER/GDP ratios between 0.06 and 2.00<br>• OAE screening strategies with lifetime treatment were also cost-effective with ICER/GDP ratios between 0.58-2.52<br>• Targeted screening strategies (iii) and (iv) were least cost-effective due to high travel costs

		**Comparator:** No screening		
**Healthcare Access – Cardiovascular Disease**				
Basu et al. (2015) [60]	India (LMI)	**Intervention:** Strategies for CVD prevention and treatment by WHO guideline: (i) primary prevention (ACEI, thiazide, CCB, statin) only; (ii) secondary prevention (ACEI, aspirin, beta-blocker, statin) only; (iii) tertiary treatment (inpatient, CABG, etc.) only; (iv) combinations of (i)-(iii)<br>**Comparator:** (i) No intervention; (ii) Comparison between strategies	Universal healthcare coverage	• All 3 types of treatment (primary, secondary, tertiary) individually cost-effective relative to no intervention<br>• Combinations that include primary prevention dominated (cost saving and health gain) those excluding primary prevention<br>• Combining all 3 types of prevention and treatment had ICER of US$1,331 per DALY averted relative to no intervention which was cost-effective at threshold of 3-times GDP per capita (US$1,524 per DALY averted). But total budgetary impact would be US$13.6 billion per year which is greater than total public healthcare budget of US$4bn.
Chen et al. (2017) [61]	China (UMI)	**Intervention:** Pharmacologic intervention with candesartan (ARB) or ramipril (ACEI) to prevent coronary heart disease (CHD), stroke and heart failure in prehypertensive adults<br>**Comparator:** No intervention (placebo)	Universal healthcare coverage	• Pharmacologic intervention had ICER of US$12,994 per QALY relative to placebo which had 30.5% probability of being cost-effective at threshold of 3-times GDP per capita
Gu et al. (2015) [62]	China (UMI)	**Intervention:** Anti-hypertensive treatment strategies to prevent CVD: (i) primary prevention – treat second stage hypertension patients (SBP >=160mmHg) only; (ii) primary prevention – treat second and first stage hypertension patients (SBP 140-159mmHg); (iii) secondary prevention – treat patients with CVD history only<br>**Comparator:** No intervention	Universal healthcare coverage	• Strategy (iii) of secondary prevention dominated (cost saving and health gain) no intervention<br>• Strategy (i) of primary prevention for second stage hypertension patients had an ICER of Int$9,059 per QALY relative to strategy (iii) of secondary prevention only which was cost-effective at threshold of 1-time GDP per capita (Int$11,900 per QALY)<br>• Strategy (ii) had an ICER of Int$10,000 per QALY relative to strategy (iii)<br>• Strategy (ii) had an ICER of Int$13,200 per QALY relative to strategy (i)
Ngalesoni et al. (2016) [63]	Tanzania (LI)	**Intervention:** Strategies for primary prevention of CVD: 32 strategies for low- and medium-risk sub-cohorts; 51 strategies for high-risk without diabetes; 51 strategies for very high-risk without diabetes; 43 for low- and medium-risk with diabetes; 49 for high-risk with diabetes; 49 for very high-risk with diabetes<br>**Comparator:** (i) No intervention; (ii) Comparison between strategies	Universal healthcare coverage	• For low-risk sub-cohort without diabetes, no strategy was cost-effective relative to no intervention unless threshold was higher than US$1327 per DALY averted<br>• For medium-risk without diabetes, ACEI+ diuretic strategy was most cost-effective with ICER of US$164 per DALY averted<br>• For high risk without diabetes, ACEI+CCB+diuretic strategy most cost-effective with ICER of US$349 per DALY averted<br>• For very high-risk without diabetes, ACEI+CCB+Diuretic+ASA most cost-effective with ICER of US$498 per DALY averted<br>• For low-risk with diabetes, ACEI+CCB+ Sulfonylureas (Sulf) most cost-effective with ICER of US$608 per DALY averted

				• For medium-risk with diabetes, ACEI+CCB+Sulf most cost-effective with ICER of US$115 per DALY averted<br>• For high-risk with diabetes, ACEI+CCB+Sulf+ Biguanide (Big) most cost-effective with ICER of US$309 per DALY averted<br>• For very high-risk with diabetes, ACEI+CCB+Sulf+Big+ASA most cost-effective with ICER of US$350 per DALY averted
Phisalprapa et al. (2017) [64]	Thailand (UMI)	**Intervention:** Early diagnosis of non-alcohol fatty liver disease (NAFLD) by ultrasonography screening plus intensive weight reduction intervention for individuals with metabolic syndrome<br>**Comparator:** No screening or weight reduction	Universal healthcare coverage	• Screening and intervention strategy had ICER of US$958 per QALY relative to no screening or intervention which was cost-effective at threshold of 3-times GDP per capita (US$4848 per QALY)<br>• Screening and intervention before 45 years old dominated (cost saving and health gain) no screening or intervention
Thongsri et al. (2016) [65]	Thailand (UMI)	**Intervention:** CVD prevention strategies for congenital heart disease patients with pulmonary arterial hypertension: (i) beraprost plus standard treatment (anticoagulation, diuretics, oxygen and digoxin); (ii) sildenafil plus standard treatment<br>**Comparator:** Standard treatment only	Universal healthcare coverage	• Beraprost plus standard treatment had ICER of THB192,752 per QALY for patients in functional class II; and ICER of THB201,308 per QALY for patients in functional class III relative to standard treatment alone<br>• Sildenafil plus standard treatment had ICER of THB249,770 per QALY for functional class II and THB226,802 per QALY for functional class III relative to standard treatment alone<br>• Sildenafil should be included in the National Drug List of Essential Medicines as first-line treatment, and its price per dose should be reduced by 43-57%
**Healthcare Access – Chronic respiratory**				
Rodriguez-Martinez et al. (2018) [66]	Colombia (UMI)	**Intervention:** Multidisciplinary structured educational intervention to improve adherence to inhaled corticosteroids for paediatric asthma patients aged 2-15<br>**Comparator:** No intervention	(i) Disadvantaged group: children; (ii) Universal healthcare coverage	• Educational intervention would dominate (cost saving and health gain) no intervention if its cost was less than US$513.20 per person<br>• Intervention would be cost-effective at threshold of 1-time GDP per capita (US$33,300 per QALY) relative to no intervention if its cost was less than US$967.40 per person
**Healthcare Access – Diabetes and kidney diseases**				
Afiatin et al (2017) [67]	Indonesia (LMI)	**Intervention:** Dialysis options as first policy for end-stage renal disease patients: (i) Haemodialysis (HD-policy); (ii) Peritoneal dialysis (PD-policy)<br>**Comparator:** Best supportive care (BSC)	Universal healthcare coverage	• Both PD- and HD-policies were not cost-effective relative to BSC at threshold of 1-time GDP per capita. The threshold would need to increase to 4-times GDP per capita for PD-policy to be cost-effective<br>• PD-policy would require 43 trillion IDR (Indonesian Rupiah) for 53% coverage and 75 trillion IDR for 100% coverage over five years; and 88 trillion IDR and 166 trillion IDR for HD-policy<br>• PD-policy dominated (cost saving and health gain) HD-policy

Cardenas et al. (2015) [68]	Peru (UMI)	**Intervention:** Diabetic foot ulcer (DFU) prevention strategies for type 2 diabetes patients with severe neuropathy with foot deformity or history of ulceration: (i) strategy based on International Diabetes Federation (IDF) recommendations; (ii) IDF recommendation plus temperature monitoring<br>**Comparator:** Usual (sub-optimal) care	Universal healthcare coverage	• IDF strategy dominated (cost saving and more deaths averted) usual care<br>• IDF recommendation plus temperature monitoring had ICER of US$16,124 per death averted relative to usual care
Home et al. (2015) [69]	Algeria (UMI); India (LMI); Indonesia (LMI); Mexico (UMI); South Korea	**Intervention:** Insulin detemir to prevent diabetes complications (vision loss, end-stage renal disease, myocardial infarction, ulcer) in type 2 diabetes patients with inadequate glycaemic control on oral glucose-lowering drug (OGLD) alone<br>**Comparator:** OGLD alone	Universal healthcare coverage	• Insulin detemir was cost-effective relative to OGLD alone in all countries at threshold of 3-times GDP per capita under 30-year and 1-year horizons
Mash et al. (2015) [70]	South Africa (UMI)	**Intervention:** Diabetes group education programme delivered by mid-level trained health promoters with a guiding style for type 2 diabetes patients<br>**Comparator:** Usual care	(i) Disadvantaged group: uninsured and underserved community; (ii) Universal healthcare coverage	• Group education programme had ICER of $1,862 per QALY relative to usual care which was cost-effective at threshold of 1-time GDP per capita ($6,003 per QALY)
Vetrini et al. (2018) [71]	Malawi (LI)	**Intervention:** Targeted annual screening and laser photocoagulation treatment for types 1 and 2 diabetes patients aged over 50 to prevent diabetic eye diseases – retinopathy and macular edema<br>**Comparator:** No screening or treatment	Universal healthcare coverage	• Screening and treatment strategy had ICER of $400 per QALY gained relative to no screening or treatment which was cost-effective at threshold of 1-time GDP per capita ($679 per QALY)<br>• The ICER was $766 per DALY averted relative to no screening or treatment which was *not* cost-effective at threshold of 1-time GDP per capita ($679 per DALY averted)
Wu et al. (2018) [72]	China (UMI)	**Intervention:** Diabetic foot ulcer (DFU) prevention strategies for newly diagnosed type 2 diabetes patients: (i) education on foot care; (ii) annual provision of footwear for high-risk patients; (iii) pharmacological therapy for peripheral neuropathy patients and topical hydrogel for DFU patients<br>**Comparator:** Usual care	Universal healthcare coverage	• All DFU prevention strategies dominated (cost saving and health gain) usual care
Wu et al. (2018c) [73]	China (UMI)	**Intervention:** Diabetic kidney disease (DKD) prevention strategies for newly diagnosed type 2 diabetes patients: (i) treatment with angiotensin-converting-enzyme (ACE) inhibitors and angiotensin II receptor blockers (ARBs) – universal strategy; (ii) screening for	Universal healthcare coverage	• Both universal and screening strategies dominated (cost saving and health gain) no intervention<br>• Universal strategy had ICER of US$30,087 per QALY which was not cost-effective relative to screening strategy at threshold of 1-time GDP per capita (US$7,380 per QALY)

		microalbuminuria followed by ACE inhibitor/ARB – screening strategy **Intervention:** No intervention		
**Healthcare Access – Mental disorders**				
Anh et al (2015) [74]	Vietnam (LMI)	**Intervention:** Pharmacological intervention for schizophrenia with or without psychosocial interventions **Comparator:** No intervention	Universal healthcare coverage	• Scenarios combining pharmaceutical intervention with family (psychosocial) intervention dominated (cost saving and health gain) no intervention
Johansson et al. (2016) [75]	Ethiopia (LI)	**Intervention:** Package of mental and neurological (MN) healthcare free of charge for: epilepsy (75% coverage, phenobarbital); depression (30% coverage, flu-oxetine, cognitive therapy and proactive case management); bipolar affective disorder (50% coverage, valproate and psychosocial therapy); schizophrenia (75% coverage, haloperidol plus psychosocial treatment) **Comparator:** No intervention	(i) Differential subgroup impact; (ii) Universal healthcare coverage	• MN package would cost US$177 million and gain 155,000 healthy-life-years (HALYs) annually: epilepsy US$37m and 64,500 HALYs; depression US$65m and 61,300 HALYs; bipolar disorder US$44m and 20,300 HALYs; and schizophrenia US$31m and 8,900 HALYs<br>• The health benefits would be concentrated among the poorest groups for all interventions<br>•
Kitwitee et al. (2017) [76]	Thailand (UMI)	**Intervention:** Video-electro-encephalography (VEEG) followed by surgery for drug-resistant focal epilepsy patients aged 35 with 15 years of disease history and 12 months of drug resistance **Comparator:** No VEEG or surgery but continued medical treatment	Universal healthcare coverage	• VEEG plus surgery had ICER of THB43,251 (US$1,236) per QALY relative to continued medical treatment which was cost-effective at threshold of 3-times GDP per capita (THB160,000 per QALY)
Linde et al. (2015) [77]	China (UMI); India (LMI); Russia (UMI); Zambia (LMI)	**Intervention:** Migraine treatment strategies: (i) simple analgesic (e.g. aspirin) as 1st-line treatment; (ii) sumatriptan and almotriptan as 2nd-line treatment for non-responders to 1st-line; (iii) prophylactic drugs (propranolol, topiramate and amitriptyline daily); (iv) addition of consumer education and provider training **Comparator:** No intervention	Universal healthcare coverage	• Strategy (i) of 1st-line treatment alone had ICER of less than US$100 per healthy-life year (HLY) gained relative to no intervention<br>• Adding education and training to other strategies did not increase the ICER above US$100 per HLY gained relative to no intervention<br>• Strategy (ii) of adding 2nd-line treatment to 1st-line would increase the ICER above US$10,000 per HLY gained
Megiddo et al. (2016) [78]	India (LMI)	**Intervention:** Epilepsy treatment strategies: (i) 1st-line anti-epilepsy drugs (AEDs); (ii) 1st and 2nd-line AEDs; (iii) 1st and 2nd-line AEDs and surgery **Comparator:** No intervention (current scenario)	(i) Differential subgroup impact; (ii) Universal healthcare coverage	• All three scenarios averted between 0.8-1 million DALYs per year relative to current scenario and were cost-effective at threshold of 1-time GDP per capita<br>• In poor regions and populations, strategy (i) did not decrease out-of-pocket (OOP) expenditure or provide financial risk protection if care-seeking costs are included

				• Strategies (ii) and (iii) had higher OOP averted than strategy (i): in the first ten years of both strategies, US$80 million OOP was averted per year
Strand et al. (2016) [79]	Ethiopia (LI)	**Intervention:** 19 different mental and neurological (MN) interventions for: (i) epilepsy; (ii) depression; (iii) bipolar disorder; (iv) schizophrenia<br>**Comparator:** No intervention	Universal healthcare coverage	• Of the 4 disease areas, epilepsy treatment with first-generation anti-epileptic drug had the lowest ICER (US$321 per DALY averted) relative to no intervention which was cost-effective at threshold of 3-times GDP per capita (US$500 per DALY averted)<br>• Depression treatments had mid-range ICERs from US$457 to US$1,026 per DALY averted relative to no intervention<br>• Treatments for schizophrenia and bipolar disorders have the highest ICERs from US$1,168 to US$3,739 per DALY averted
**Healthcare Access – Musculoskeletal disorders**				
Eeson et al. (2015) [80]	Uganda (LI)	**Intervention:** Paediatric inguinal hernia repair (PIHR) for children aged less than 18 with inguinal hernia<br>**Comparator:** No intervention	(i) Disadvantaged group: children; (ii) Universal healthcare coverage	• PIHR had ICER of $12.41 per DALY averted relative to no intervention and had 95% probability of being cost-effective relative to no intervention at threshold of $35 per DALY averted
**Healthcare Access – Neoplasms (Breast Cancer)**				
Ansaripour et al.(2018) [81]	Iran (UMI)	**Intervention:** Trastuzumab therapy of different durations: (i) 6 months; (ii) 9 months; (iii) 1 year<br>**Comparator**: No trastuzumab	(i) Disadvantaged group: women; (ii) Universal healthcare coverage	• Incremental costs (versus no trastuzumab) were €8826 (6 months), €13,808 (9 months) and €18,588 (12 months), while incremental quality-adjusted life-years (QALYs) were 0.65 (6 months), 0.87 (9 months) and 1.14 (12 months). At a threshold of 3 gross domestic product (GDP)/capita (€21,000/QALY) and for patients younger than 59 years, the 6-month protocol was most likely to be cost effective (probability of 42%).
Pichon-Riviere et al. (2015) [82]	Argentina (UMI); Bolivia (LMI); Brazil (UMI); Colombia (UMI); Chile; Peru (UMI); Uruguay	**Intervention:** Adjuvant trastuzumab therapy of 12 months for early HER2-positive breast cancer patients aged 55<br>**Comparator:** No adjuvant trastuzumab therapy	(i) Disadvantaged group: women; (ii) Universal healthcare coverage	• Access to trastuzumab had ICERs (incremental cost per QALY) ranging from 35.5 times GDP per capita in Bolivia (LMI) to 3.6 times in Uruguay (a high-income country).<br>• The price of trastuzumab would have to fall by 94.9% in Bolivia and by 69.6% in Uruguay for trastuzumab to become cost-effective at threshold of 1-time GDP per capita
**Healthcare Access – Neoplasms (Lung Cancer)**				
Lu et al. (2018) [83]	China (UMI)	**Intervention:** Pemetrexed plus best supportive care (BSC) for non-small-cell lung cancer patients<br>**Comparator:** BSC only	Universal healthcare coverage	• Pemetrexed plus BSC yielded additional 0.12 QALYs per patient relative to BSC only<br>• Average additional cost per patient was $48,034.46 under Patient Assistance Program (PAP) and $96,191.57 without PAP. Hence PAP reduced the percentage of patients experiencing catastrophic healthcare expenditure from 98.39%

				to 19.91% and post-intervention poverty headcount ratio from 66.98% to 4.89%.
Shi et al. (2017) [84]	China (UMI)	**Intervention:** Pemetrexed plus best supportive care (BSC) for advanced non-squamous non-small-cell lung cancer patients<br>**Comparator:** BSC only	Universal healthcare coverage	• Pemetrexed plus BSC had ICER of US$24,319 per QALY under PAP relative to BSC only which was cost-effective at threshold of 3-times GDP per capita (US$39,900 per QALY) but ICER of US$222,700 per QALY without PAP
Throngprasert and Permsuwan (2017) [85]	Thailand (UMI)	**Intervention:** ALK testing plus crizotinib treatment for non-small-cell lung cancer patients<br>**Comparator:** No testing or treatment	Universal healthcare coverage	• The total budget impact of ALK testing and crizotinib treatment was US$3,480,507 in first year of coverage, US$2,526,498 in second year and US$1,184,167 in third year<br>• Per-patient monthly costs would be US$1,142 in first year, US$575 in second year and USUS244 in third year.<br>• ALK testing and crizotinib treatment is likely to be affordable only to patients in the upper middle or high socioeconomic status
**Healthcare Access – Neoplasms (Leukaemia)**				
Sheng et al. (2017) [86]	China (UMI)	**Intervention:** Patient Assistance Program (PAP) coverage of imatinib and nilotinib as first-line treatment for newly diagnosed chronic myeloid leukaemia (CML) patients<br>**Comparator:** No PAP coverage of imatinib and nilotinib	Universal healthcare coverage	• PAP coverage of imatinib had ICER of CYN50,641 per QALY relative to no coverage which was cost-effective at threshold of 1-time GDP per capita
Wu et al. (2017) [87]	China (UMI)	**Intervention:** Alternatives to low-dose 600mg imatinib for patients with chronic phase CML who are resistant to 600mg imatinib: (i) 800mg imatinib; (ii) dasatinib; (iii) nilotinib<br>**Comparator:** (i) Standard dose 600mg imatinib; (ii) Comparison between alternatives	Universal healthcare coverage	• Dasatinib had ICER of $16,417 per QALY relative to standard 600mg imatinib when Patient Assistance Program (PAP) was available<br>• Dasatinib dominated (cost saving and health gain) both 800mg imatinib and nilotinib
**Healthcare Access – Neoplasms (Other Cancers)**				
Shrime et al. (2016) [88]	Uganda (LI)	**Intervention:** Strategies to provide cancer surgery: (i) universal public financing (UPF); (ii) task shifting to non-surgeon providers (TS); (iii) UPF + training of non-surgeons (UPFTS); (iv) UPF + vouchers for non-medical costs; (v) TS + vouchers; (vi) UPFTS + vouchers; (vii) charitable 2-week surgical trip (2W); (viii) charitable mobile surgical units (MS); (ix) charitable cancer hospitals (CH)<br>**Comparator:** No surgery provision	(i) Differential subgroup impact; (ii) Universal healthcare coverage	• Of the nine strategies, strategies (iv), (vi) and (viii) provided both health benefit (deaths averted) and financial benefits (catastrophic expenditure and impoverishment protection) and were on the efficiency frontier (not dominated by other strategies)<br>• Strategies that did not provide patient vouchers and charitable mobile surgical units were dominated by other strategies
Zhang et al. (2015) [89]	China (UMI)	**Intervention:** Sorafenib as first-line treatment for advanced hepatocellular carcinoma<br>**Comparator:** Best supportive care (BSC)	Universal healthcare coverage	• Sorafenib had ICER of $101,399 per QALY relative to BSC which was not cost-effective at threshold of 3-times GDP per capita

Zhou et al. (2017) [90]	China (UMI)	**Intervention:** Second-line chemotherapy based on irinotecan, docetaxel and/or paclitaxel for advanced gastric cancer patients aged over 70<br>**Comparator:** Best supportive care (BSC)	(i) Disadvantaged group: elderly; (ii) Universal healthcare coverage	• Second-line chemotherapy had ICER of $18,224 per QALY relative to BSC which was cost-effective at threshold of 3-times GDP per capita ($23,970 per QALY)
**Healthcare Access – Other non-communicable diseases**				
Moran et al. (2017) [91]	Cambodia (LMI)	**Intervention:** Craniotomy for epidural hematoma inpatients at a tertiary hospital<br>**Comparator:** No craniotomy	Universal healthcare coverage	• Craniotomy had ICER of $574.93 per QALY relative to no craniotomy which was cost-effective at threshold of 3-times GDP per capita ($9,787.80 per QALY)
Semwanga et al. (2016) [92]	Uganda (LI)	**Intervention:** Strategies to improve maternal and neonatal health: (i) interventions to increase demand for maternal/neonatal healthcare services (e.g. free delivery kit, motorcycle coupons); (ii) interventions to improve health service delivery conditions; (iii) interventions to improve mother's health<br>**Comparator:** No intervention	(i) Disadvantaged group: neonates; (ii) Universal healthcare coverage	• Intervention with highest impact on reducing neonatal mortality was combination of free delivery kits (strategy (i)) in a setting where delivery services were free (strategy (ii)) and motorcycle coupons to take women to hospital during emergencies (strategy (i))
Verguet et al. (2015) [93]	Ethiopia (LI)	**Intervention:** 9 interventions by disease areas: (i) caesarean section surgery for maternal health; (ii) anti-hypertensive treatment; (iii)-(ix) treatments for other conditions (e.g. measles vaccination, diarrhoea treatment)<br>**Comparator:** No intervention	(i) Differential subgroup impact; (ii) Universal healthcare coverage	• Caesarean section surgery averted around 140 deaths per US$100,000 invested which was third most out of 9 interventions<br>• Anti-hypertensive treatment averted around 11 deaths per US$100,000 invested which was eighth out of 9 interventions<br>• Caesarean section surgery averted around 98 poverty cases per US$100,000 invested which was first out of 9 interventions<br>• Anti-hypertensive treatment averted around 85 poverty cases per US$100,000 invested which was third out of 9 interventions
Watkins et al. (2015) [94]	Cuba (UMI)	**Intervention:** Acute rheumatic fever (ARF) and rheumatic heart disease (RHD) control programme for children aged 5-24 combining primary prevention (e.g. provider education), secondary prevention (e.g. improving adherence to penicillin prophylaxis) and heart valve surgery for severe chronic RHD<br>**Comparator:** No intervention	Universal healthcare coverage	• Control programme dominated (cost saving and health gain) no intervention<br>• Control programme would cost $202,890 over 10 years
Watkins et al. (2016) [95]	Hypothetical African country (LI)	**Intervention:** Increased coverage rates of acute rheumatic fever (ARF) and rheumatic heart disease (RHD) control components for children aged 5-24: (i) primary prevention (improved treatment of pharyngitis) - 10% to 70% coverage; (ii) secondary prevention (e.g. improved adherence to penicillin prophylaxis) - 10% to 92%; (iii) heart valve surgery for	Universal healthcare coverage	• Increased coverage of primary prevention alone dominated (cost saving and health gain) no intervention<br>• Increased coverage of secondary prevention was cost-effective, with ICER less than 1-time GDP per capita threshold<br>• Increased coverage of valve surgery would be cost-effective under existing (foreign) surgical capacity but not so without it: ICER exceeds 3-times GDP per capita threshold

		severe chronic RHD (de novo investment or using existing surgical services in foreign countries) - 10% to 95%<br>**Comparator:** No increased coverage		
**Healthcare Access – Sense organ diseases**				
Emmett et al. (2015) [96]	Sub-Saharan Africa (LI/LMI/UMI)	**Intervention:** Hearing improvement strategies for children aged less than 36 months with congenital hearing loss: (i) Cochlear implantation; (ii) Deaf education<br>**Comparator:** No intervention	(i) Disadvantaged group: children; (ii) Universal healthcare coverage	• Cochlear implantation was cost-effective in South Africa (ICER/GDP ratio of 1.03) and Nigeria (ICER/GDP ratio of 2.05)<br>• Deaf education was cost-effective in all countries (ICER/GDP ratio ranging from 0.55 to 1.56)
Guedes et al. (2016) [97]	Brazil (UMI)	**Intervention:** Treatment strategies for early, moderate or severe primary open-angle glaucoma: (i) Medical treatment; (ii) Laser treatment; (iii) Surgical treatment<br>**Comparator:** Observation only	Universal healthcare coverage	• For low severity glaucoma, strategy (i) and strategy (ii) were cost-effective relative to observation only at threshold of 3-times GDP per capita<br>• For moderate severity glaucoma, strategy (ii) and strategy (iii) were cost-effective relative to observation only at threshold of 3-times GDP per capita<br>• For advanced severity glaucoma, strategy (i) and strategy (iii) were cost-effective relative to observation only at threshold of 3-times GDP per capita
**Healthcare Access – Skin disease**				
Wu et al. (2018b) [98]	China (UMI)	**Intervention:** Strategies for preventing recurrent endometriosis for women who had undergone conservative laparoscopic or laparotomic surgery: (i) oral contraceptives; (ii) three months of gonadotropin-releasing hormone therapy (GnRH-3); (iii) six months of GnRH therapy (GnRH-6)<br>**Comparator:** No intervention	Universal healthcare coverage	• Out of three strategies, GnRH-6 had the lowest ICERs of US$6,364, US$6,425, US$6,185 and US$6,407 per QALY relative to no intervention for ovarian endometriosis, peritoneal endometriosis, deep endometriosis and other endometriosis, respectively which were all cost-effective at threshold of 1-time GDP per capita (US$7,400 per QALY)
**Healthcare Access – Substance use**				
Kessler et al. (2015) [99]	East Africa, particularly Kenya (LI/LMI)	**Intervention:** Cognitive behavioural therapy (CBT) targeting strategies to reduce hazardous alcohol consumption among HIV patients: (i) all HIV-infected persons attending HIV outpatient clinic; (ii) only those in pre-ART care stage; (iii) only those in ART care stage; (iv) only those with detectable viral loads regardless of disease stage<br>**Comparator:** No intervention	Universal healthcare coverage	• Strategy (i) of universal targeting had ICER of $600 per QALY relative to no intervention which was cost-effective at threshold of 3-times GDP per capita ($2,800 per QALY)<br>• Narrower targeting strategies (ii) and (iv) both dominated (cost saving and health gain) no intervention
Tosanguan and Chaiyakunapruk (2016) [100]	Thailand (UMI)	**Intervention:** Clinical smoking cessation interventions for adults aged over 40: (i) Counselling only in hospital; (ii) Quitline; (iii)	Universal healthcare coverage	• All seven strategies individually dominated (cost saving and health gain) no intervention

		Counselling with nicotine gum; (iv) Counselling with nicotine patch; (v) Counselling with nortriptyline; (vi) Counselling with bupropion; (vii) Counselling with varenicline<br>**Comparator:** (i) No intervention; (ii) Comparison between strategies		• Compared to each other, strategy (v) and strategy (vii) were the only non-dominated strategies
				•
**Comparative Evaluation – Cardiovascular diseases**				
Basu et al. (2016) [101]	China (UMI); India (LMI)	**Intervention:** Strategies for treating high blood pressure: (i) European Guideline: benefit-based tailored treatment (BTT) – treat people with 10-year CVD risk greater than 10%; (ii) WHO Guideline: hybrid strategy with BTT and TTT<br>**Comparator:** (i) US Guideline – treat-to-target (TTT) strategy; (ii) No treatment	None	• BTT strategy averted 5 million additional DALYs in both China and India relative to TTT while incurring similar cost<br>• Hybrid strategy produced similar result to TTT, and hence BTT dominated (cost saving and health gain) both TTT and Hybrid<br>• BTT was cost-effective relative to no treatment with ICERs between $205 and $272 per DALY averted
Borissov et al. (2017) [102]	Bulgaria (UMI)	**Intervention:** Evolocumab plus statin for patients with familial hypercholesterolaemia to prevent CVD (MI, stroke, heart failure)<br>**Comparator:** Statin alone	None	• Evolocumab plus statin had ICER of $6,604 per effectively treated patient year (threshold not given)
Cui et al. (2016) [103]	China (UMI)	**Intervention:** 1-year treatment of clopidogrel plus aspirin for non-ST-segment elevation acute coronary syndrome within 24 hours of acute coronary syndrome to prevent vascular death, MI and stroke<br>**Comparator:** Aspirin alone	None	• Clopidogrel plus aspirin had ICER of CYN43,340 per QALY relative to aspirin alone which had 88% probability of being cost-effective at threshold of 3-times GDP per capita (CYN150,721 per QALY)
Giorgi et al. (2015) [104]	Argentina (UMI)	**Intervention:** Apixaban for patients with non-valvular atrial fibrillation (NVAF)<br>**Comparator:** Vitamin K antagonist Warfarin	None	• Apixaban had ICER of US$786.08 per QALY relative to Warfarin which was cost-effective at threshold of 1-time GDP per capita (US$11,558 per QALY)
Konfino et al. (2017) [105]	Argentina (UMI)	**Intervention:** Strategies with high-potency statins for adults aged 35-84 to prevent CVD (stroke, CHD): (i) lower threshold of treatment to >10% Framingham CVD risk; (ii) intensify statin potency (atorvastatin, rosuvastatin) under current treatment threshold; (iii) lower treatment threshold to >10% and intensify statin potency<br>**Comparator:** Current CVD prevention strategy with low- or moderate-potency statins (simvastatin) and treatment threshold of >20% Framingham risk score or diabetes	None	• ICERs relative to current strategy were: strategy (i) US$19,900 per QALY; strategy (ii) US$42,200 per QALY; strategy (iii) US$33,100 per QALY. Strategies were all cost-effective at threshold of 3-times GDP per capita (US$44,280 per QALY)<br>• Scenario (ii) was not cost-effective under higher compliance rate (75%) and higher toxicity of high-potency statin

Permsuwan et al. (2015) [106]	Thailand (UMI)	**Intervention:** Fondaparinux 2.5mg once daily for non-ST-segment elevation acute coronary syndrome patients aged over 60<br>**Comparator:** Enoxaparin 1mg/kg body weight twice daily	None	• Fondaparinux dominated (cost saving and health gain) enoxaparin from both healthcare and societal perspectives<br>•
Ribeiro et al. (2015) [107]	Brazil (UMI)	**Intervention:** Statin dosing strategies for adults aged 45-85 with or without recent CVD event: (i) low dose - LDL reduction less than 30%; (ii) intermediate dose - LDL reduction 30-40%; (iii) high dose - LDL reduction greater than 40%; (iv) no statin. Five initiation scenarios: (a) secondary prevention only; (b) primary prevention - ten-year CVD risk of 5%; (c) primary - risk of 10%; (d) primary - risk of 15%; (e) primary - risk of 20%<br>**Comparator:** Comparison between strategies	None	• Low dose strategies extendedly dominated by other strategies in the four primary prevention initiation scenarios (b) to (e)<br>• In all five initiation scenarios, ICER of intermediate dose strategy below US$10,000 per QALY relative to no statin strategy which was cost-effective at threshold of 1-time GDP per capita (Int$11,770 per QALY)<br>• In all five initiation scenarios, ICER of high dose strategy above US$27,000 per QALY relative to intermediate dose strategy<br>• In all statin dosing strategies, all primary prevention scenarios (b) to (e) dominated (health gain and cost saving) scenario (a) of secondary prevention
Xie et al. (2018) [108]	China (UMI)	**Intervention:** Intensive hypertension control for hypertensive adults aged 35-84 - reduce SBP/DBP to 133/76mmHg with three drugs at half standard dose each<br>**Comparator:** Standard hypertension control (based on 2011 Chinese guideline) - reduce SBP/DBP to 140/90mmHg with one drug at standard dose	None	• Under 10-year horizon, intensive control strategy had ICER of CYN7,876 per QALY relative to standard strategy which was cost-effective at threshold of 1-time GDP per capita (CYN46,491 per QALY)<br>• Under 20-year horizon, intensive control strategy had ICER of CNY5,811 per QALY relative to standard strategy which was cost-effective at above threshold
Xuan et al. (2018) [109]	China (UMI)	**Intervention:** Two-week safflower yellow injection (SYI) plus conventional (western) treatment for stable angina patients to prevent unstable angina and CVD mortality<br>**Comparator:** Two-week conventional treatment only: oral anticoagulants, beta blockers, statins, nitrate esters	None	• SYI plus conventional treatment had ICER of US$3,791 per QALY relative to conventional treatment which was cost-effective at threshold of 1-time GDP per capita (US$8,130 per QALY)
**Comparative Evaluation – Chronic respiratory**				
Rodriguez-Martinez et al. (2015) [110]	Colombia (UMI)	**Intervention:** Daily administration of inhaled corticosteroids (ICS) for paediatric asthma<br>**Comparator:** Intermittent or as-needed administration of ICS	Disadvantaged group: children	• Daily ICS dominates (cost saving and health gain) intermittent ICS for both preschool and school children cohorts
Rodriguez-Martinez et al. (2016) [111]	Colombia (UMI)	**Intervention:** Once-daily inhaled corticosteroids (ICS) for paediatric asthma – has higher adherence than twice-daily ICS but same therapeutic effect given same adherence<br>**Comparator:** Twice-daily ICS	Disadvantaged group: children	• Once-daily ICS dominates (cost saving and health gain) twice-daily ICS

Suzuki et al. (2017) [112]	Brazil (UMI)	**Intervention:** Omalizumab add-on to standard care for asthma patients with mean age 45<br>**Comparator:** Standard care alone (inhaled corticosteriods + long-acting beta agonist + oral corticosteriods + short-acting beta agonist)	None	• Omalizumab plus standard care had ICER of R$53,890 per QALY which was cost-effective at threshold of 3-times GDP per capita
**Comparative Evaluation – Diabetes and kidney diseases**				
Balderas-Pena et al. (2016) [113]	Mexico (UMI)	**Intervention:** Early (within 48-hours of admission) microbiological culture testing which allows tailored antibiotic treatment for infected diabetic foot ulcers by microbe type (e.g. its antibiotic susceptibility)<br>**Comparator:** No culture testing before antibiotic treatment	None	• Early microbiological culture testing before antibiotic treatment reduced treatment costs by 10-25% and by more than 30% if length of hospital stay was also considered as economic consequence
Carlos et al. (2015) [114]	Venezuela (UMI)	**Intervention:** Duloxetine as first-line therapy for painful diabetic peripheral neuropathy<br>**Comparator:** Pregabalin as first-line therapy	None	• Duloxetine dominates (cost saving and health gain) pregabalin
Deng et al. (2015) [115]	China (UMI)	**Intervention:** Exenatide twice daily as add-on therapy for diabetes patients aged over 40 not well controlled by oral anti-diabetic agents<br>**Comparator:** Insulin glargine once daily (QD) as add-on therapy	None	• Exenatide as add-on therapy dominated (cost saving plus health gain) insulin glargine as add-on therapy
Gomez et al. (2016) [116]	Colombia (UMI)	**Intervention:** Integrated pump technology insulin therapy for type 1 diabetes patients<br>**Comparator:** Multiple daily injection of insulin	None	• Integrated pump therapy had ICER of US$24,000 per QALY relative to multiple insulin injection which was cost-effective at threshold of 3-times GDP per capita (US$26,750 per QALY)
Gu et al. (2015b) [117]	China (UMI)	**Intervention:** Saxagliptin plus metformin for type 2 diabetes patients without adequate glycaemic control with metformin alone<br>**Comparator:** Glimepiride plus metformin	None	• Saxagliptin plus metformin dominated (cost saving and health gain) glimepiride plus metformin
Gu et al. (2016) [118]	China (UMI)	**Intervention:** Saxagliptin plus metformin for type 2 diabetes patients without adequate glycaemic control with metformin alone<br>**Comparator:** Acarbose plus metformin	None	• Saxagliptin plus metformin dominated (cost saving and health gain) acarbose plus metformin
Gupta et al. (2015) [119]	India (LMI); Indonesia (LMI); Saudi Arabia	**Intervention:** Biphasic insulin aspart 30 (BIAsp 30) with or without oral glucose-lowering drugs (OGLDs) for insulin-experienced type 2 diabetes patients to prevent diabetic complications (vision loss, end-stage renal disease, MI, ulcer)<br>**Comparator:** Monotherapy with biphasic human insulin 30 (BHI), insulin glargine	None	• Switching to BIAsp 30 from BHI, IGlar or NPH was associated with increase of life expectancy of >0.7 years and considered cost-effective (ICER less than 3-times GDP per capita) or highly cost-effective (ICER less than 1-time GDP per capita) in India, Indonesia and Saudi Arabia under 30-year horizon<br>• Cost-effectiveness was maintained in short-run 1-year analysis

		(IGlar) or neutral protamine Hagedorn (NPH) insulin with or without OGLDs		
Permsuwan et al. (2016) [120]	Thailand (UMI)	**Intervention:** Insulin glargine (IGlar) for type 2 diabetes patients without adequate glycaemic control under two oral anti-diabetic drugs<br>**Comparator:** Neutral protamine Hagedorn (NPH) insulin	None	• IGlar had ICER of THB244,915 per QALY relative to NPH insulin which was not cost-effective at commonly used threshold of THB160,000 per QALY (about 1.2-times GDP per capita)
Shao et al. (2017) [121]	China (UMI)	**Intervention:** Dapagliflozin as monotherapy for type 2 diabetes patients with mean age 51 to prevent micro- and macrovascular complications<br>**Comparator:** Glimepiride as monotherapy	None	• Dapagliflozin dominated (cost saving and health gain) glimepiride
**Comparative Evaluation – Digestive diseases**				
Xuan et al. (2016) [122]	China (UMI)	**Intervention:** Ilaprazole 10mg once daily for newly diagnosed duodenal ulcer patients<br>**Comparator:** Omeprazole 20mg once-daily	None	• Ilaprazole therapy had ICER of CNY132,056 per QALY which was cost-effective at threshold of 3-times GDP per capita<br>•
**Comparative Evaluation – Mental disorders**				
Lubinga et al. (2015) [123]	Uganda (LI)	**Intervention:** Antipsychotics for schizophrenia patients aged over 25: (i) chlorpromazine; (ii) haloperidol; (iii) risperidone; (iv) olanzapine; (v) quetiapine<br>**Comparator:** Comparison between strategies	None	• Risperidone dominated (cost saving and health gain) haloperidol, quetiapine and chlorpromazine.<br>• Olanzapine averted more DALYs but was more costly than risperidone with ICER of US$5,868 per DALY averted which was not cost-effective at threshold of 3-times GDP per capita (US$1,641 per DALY averted)
**Comparative Evaluation – Musculoskeletal disorders**				
Bamrungsawad et al. (2015) [124]	Thailand (UMI)	**Intervention:** Intravenous immunoglobulin (IVIG) plus corticosteroids (CS) as 2nd-line treatment for steroid-refractory dermatomyositis patients<br>**Comparator:** Immunosuppressant plus corticosteroids as 2nd-line treatment	None	• IVIG and CS strategy dominated (cost saving and health gain) immunosuppressant and CS strategy
Wu et al. (2015) [125]	China (UMI)	**Intervention:** Eight treatment sequences for moderately active rheumatoid arthritis patients: (i) Yisaipu (biosimilar of branded etanercept) 50mg/week 9 months > Yisaipu 50mg/week maintenance > rituximab > tDMARD; (ii) Yisaipu 50mg 9m + methotrexate (MTX) maintenance > Yisaipu 50mg 9m > Yisaipu 25mg maintenance > rituximab > tDMARD; (iii) Yisaipu 50mg 9m > Yisaipu 25mg maintenance > rituximab > tDMARD; (iv) Yisaipu 50mg 9w > MTX > rituximab >	None	• Relative to tDMARD only, strategy (viii) had the lowest ICER of $8,680 per QALY which was cost-effective at threshold of 3-times per GDP per capita (US$18,300 per QALY in China and US$41,400 per QALY in Shanghai)<br>• Other non-dominated strategies were: strategy (vi) ($18,224 per QALY), strategy (vii) ($19,441 per QALY) and strategy (iii) ($31,589 per QALY)<br>• Strategies (i), (ii), (iv) and (v) were dominated

		tDMARD; (v) Yisaipu 50mg 9m > Yisaipu 50mg maintenance > tDMARD; (vi) Yisaipu 50mg 9m > MTX > Yisaipu 50mg 9m > Yisaipu 25mg maintenance > tDMARD; (vii) Yisaipu 50mg 9m > Yisaipu 25mg maintenance > tDMARD; (viii) Yisaipu 50mg 9m > MTX > tDMARD<br>**Comparator:** Traditional disease-modifying anti-rheumatic drug (tDMARD) only		
Yan et al. (2016) [126]	China (UMI)	**Intervention:** Apixaban for patients after total knee replacement to prevent venous thromboembolism<br>**Comparator:** Enoxaparin	None	• Apixaban had ICER of US$108,497 per QALY relative to enoxaparin which was not cost-effective at threshold of 3-times GDP per capita (US$22,140 per QALY)
Yan et al. (2017) [127]	China (UMI)	**Intervention:** Treatment strategies for patients after total hip replacement to prevent venous thromboembolism: (i) Rivaroxaban; (ii) Apixaban; (iii) Enoxaparin<br>**Comparator:** Comparison between strategies	None	• Apixaban had ICER of US$71,244 per QALY relative to enoxaparin which was not cost-effective at threshold of 3-times GDP per capita (US$22,140 per QALY)<br>• Enoxaparin dominated (cost saving and health gain) rivaroxaban
**Comparative Evaluation – Neoplasms (Breast Cancer)**				
Bargallo-Rocha et al. (2015) [128]	Mexico (UMI)	**Intervention:** 21-gene assay to inform decision on adjuvant chemotherapy for early hormone-receptor positive and HER2-negative breast cancer<br>**Comparator:** No gene assay	Disadvantaged group: women	• Gene assay and treatment had ICER of US$1,914 per life-year gained relative to no assay before treatment which was cost-effective at threshold of 3-times GDP per capita (US$32,750 per life-year gained)
Diaby et al. (2017) [129]	Mexico (UMI)	**Intervention:** Treatment sequences for metastatic HER2-positive breast cancer: (i) 1st-line – pertuzumab, trastuzumab plus taxane (THP); 2nd-line – trastuzumab, lapatinib, pertuzumab plus ado-trastuzumab emtansine (T-DM1); 3rd-line – capecitabine plus lapatinib (CL) (THP > T-DM1 > CL); (ii) THP > trastuzumab plus lapatinib (TL) > trastuzumab plus capecitabine (THP > TL > TC); (iii) trastuzumab plus docetaxel (TD) > T-DM1 > trastuzumab plus lapatinib (TL) (TD > T-DM1 > TL); (iv) trastuzumab plus docetaxel > trastuzumab plus lapatinib > trastuzumab plus capecitabine (TD > TL > TC)<br>**Comparator:** Comparison between strategies	Disadvantaged group: women	• From public sector perspective, sequences (i), (ii) and (iii) which contain pertuzumab and/or T-DM1 were not cost-effective relative to sequence (iv) without T-DM1 or pertuzumab at threshold of $50,000 per QALY<br>• From private sector perspective, sequence (iii) which contains T-DM1 but not pertuzumab proved cost-effective relative to sequence (iv) at threshold of $50,000 per QALY
Hatam et al. (2016) [130]	Iran (UMI)	**Intervention:** Intensive follow-up strategy after breast cancer treatment<br>**Comparator:** Standard follow-up strategy	Disadvantaged group: women	• Intensive follow-up strategy had ICER of US$148,196 per case detected which is unlikely to be cost-effective at a reasonable cost-effectiveness threshold

Khan et al. (2017) [131]	Non-specific LMIC (LI/LMI/UMI)	**Intervention:** Accelerated radiotherapy (RT) schedule for breast cancer patients after primary lumpectomy or mastectomy<br>**Comparator:** Conventional RT schedule after lumpectomy or mastectomy	Disadvantaged group: women	• Accelerated schedule resulted in more women being alive and remaining disease-free relative to conventional schedule, with an absolute difference of about 4% and 7% at 15 years, respectively.<br>•
Rodriguez-Aguilar et al. (2018) [132]	Mexico (UMI)	**Intervention:** Increasing the monitoring period after breast cancer treatment from standard 5 years to 10 years<br>**Comparator:** Standard 5-year monitoring	(i) Disadvantaged group: women; (ii) Universal healthcare coverage	• The total budget impact of increased monitoring would be Mex$3,607.4 million under base scenario, Mex$4,151.79 million under pessimistic scenario and Mex$3,414.85 million under optimistic scenario<br>• This additional expenditure represents 9.1% increase in annual expenditure for breast cancer and 3% of budget of Fund for Protection against Catastrophic Expenditure (FPGC). Hence the increased monitoring does not impose a financial risk to FPGC's sustainability
**Comparative Evaluation – Neoplasms (Lung Cancer)**				
Arrieta et al. (2016) [133]	Mexico (UMI)	**Intervention:** EGFR gene mutation testing followed by chemotherapy with gefitinib or carboplatin-paclitaxel for non-small-cell lung cancer patients<br>**Comparator:** Chemotherapy without gene testing	None	• Gene testing and chemotherapy strategy had ICER of US$3,879 per progression-free survival month relative to no gene testing<br>•
Limwattananon et al. (2018) [134]	Thailand (UMI)	**Intervention:** EGFR gene mutation testing followed by one of tyrosine kinase inhibitors (TKIs) for mutation for advanced non-small-cell lung caner patients: (i) gefitinib; (ii) erlotinib; (iii) afatinib; and platinum doublets for no mutation<br>**Comparator:** (i) No EGFR testing and platinum doublets for all; (ii) Comparison between TKI strategies	None	• EGFR testing plus erlotinib had ICER of US$46,783 per QALY relative to no EGFR testing and platinum doublets for all which was not cost-effective at threshold of 1-time GDP per capita (US$4,500 per QALY)<br>• EGFR testing plus erlotinib dominated (cost saving and health gain) EGFR testing plus gefitinib<br>• EGFR testing plus afatinib had ICER of US$198,961 per QALY relative to EGFR testing plus erlotinib
Lu et al. (2016) [135]	China (UMI)	**Intervention:** ALK gene testing strategies followed by crizotinib for mutation for advanced non-small-cell lung cancer patients: (i) ventena immunohistochemistry (IHC); (ii) quantitative real-time reverse transcription-polymerase chain reaction; (iii) IHC testing plus fluorescent in situ hybridisation confirmation for anaplastic lymphoma kinase testing<br>**Comparator:** Pemetrexed plus cisplatin (PC)	None	• Strategy (i) had ICERs of US$16,820 and US$223,242 per QALY relative to PC with and without Patient Assistance Program (PAP), respectively<br>• Strategy (ii) had ICERs of US$24,424 and US$223,271 per QALY relative to PC with and without PAP, respectively<br>• Strategy (iii) had ICERs of US$16,850 and US$254,668 per QALY relative to PC with and without PAP, respectively
Lu et al. (2017) [136]	China (UMI)	**Intervention:** Treatment strategies for advanced non-small-cell lung cancer patients:	None	• Strategy (ii) had ICER of US$104,657 per QALY relative to strategy (i)

		(i) pemetrexed plus cisplatin (PC); (ii) PC followed by maintenance with pemetrexed; (iii) EGFR-specific gefitinib; (iv) EGFR-specific icotinib<br>**Comparator:** Comparison between strategies		• Strategy (iii) had ICERs of US$28,485 and US$22,577 per QALY relative to strategy (i) with and without Patient Assistance Program (PAP), respectively<br>• Strategy (iv) had ICERs of US$19,809 and US$15,451 per QALY relative to strategy (i) with and without PAP, respectively
Zhan et al. (2017) [137]	China (UMI)	**Intervention:** Bevacizumab maintenance following pemetrexed plus cisplatin (PCB) strategy for unresectable malignant pleural mesothelioma<br>**Comparator:** Pemetrexed plus cisplatin (PC) alone	None	• PCB strategy had ICERs of US$727,203 and US$221,186 per QALY relative to PC alone with and without Patient Assistance Programme, respectively, which exceeded the threshold of 3-times GDP per capita (US$23,970 per QALY)
Zheng et al. (2018) [138]	China (UMI)	**Intervention:** Bevacizumab plus paclitaxel-carboplatin (B+PC) strategy for metastatic non-small-cell lung cancer patients<br>**Comparator:** Paclitaxel plus carboplatin alone	None	• B+PC strategy had ICER of US$299,155 per QALY relative to paclitaxel plus carboplatin alone which exceeded the threshold of 3-times GDP per capita (US$23,970 per QALY)
Zhou et al. (2017b) [139]	China (UMI)	**Intervention:** Combination chemotherapy with cisplatin, etoposide and irinotecan for sensitive relapsed small-cell lung cancer<br>**Comparator:** Topotecan alone	None	• Combination chemotherapy had ICER of US$26,720 per QALY relative to topotecan alone which was not cost-effective at threshold of 3-times GDP per capita (US$24,423 per QALY)
**Comparative Evaluation – Neoplasms (Liver Cancer)**				
Lee et al. (2016) [140]	Thailand (UMI) and South Korea	**Intervention:** Enhanced magnetic resonance imaging (MRI) detection of hepatocellular carcinoma using Gd-EOB-DTPA (gadolinium-ethoxybenzyl-diethylenetriamine-pentaacetic acid)<br>**Comparator:** Standard detection: (i) extracellular contrast median-enhanced MRI (ECCM-MRI); (ii) multi-detector computed tomography (MDCT)	None	• From payer (e.g. public sector) perspective, Gd-EOB-DTPA-MRI was the least costly option with US$702 per patient to reach complete treatment decision, compared to US$931 per patient for ECCM-MRI and US$873 for MDCT<br>• From hospital perspective, the costs were US$1,106, US$1,178 and US$1,087 per patient to reach complete treatment decision for Gd-EOB-DTPA-MRI, ECCM-MRI and MDCT respectively
Zhang et al. (2016) [141]	China (UMI)	**Intervention:** FOLFOX4 strategy for advanced hepatocellular carcinoma: oxaliplatin on day 1; leucovorin on days 1 and 2; fluorouracil on days 1 and 2 and once every 2 weeks<br>**Comparator:** Sorafenib twice daily	None	• FOLFOX4 was less costly than sorafenib but also produced less health gain<br>• Sorafenib had ICER of US$934,802 per QALY relative to FOLFOX4 which was not cost-effective at threshold of 3-times GDP per capita (US$20,301 per QALY)
**Comparative Evaluation – Neoplasms (Pancreatic Cancer)**				
Zhou et al. (2015) [142]	China (UMI)	**Intervention:** (i) Gemcitabine (GEM); (ii) S-1; (iii) GEM plus S-1 (GS) for advanced/metastatic pancreatic cancer<br>**Comparator:** Comparison between strategies	None	• Strategy (ii) dominates (cost saving and health gain) both strategies (i) and (iii)

Zhou et al. (2016) [143]	China (UMI)	**Intervention:** Fluorouracil, leucovorin, irinotecan and oxaliplatin (FOLFIRINOX) for metastatic pancreatic cancer<br>**Comparator:** Gemcitabine + nab-paclitaxel (GEM-N)	None	• FOLFIRINOX had ICER of US$32,020 per QALY relative to GEM-N which was not cost-effective at threshold of 3-times GDP per capita threshold (US$20,301 per QALY)
**Comparative Evaluation – Neoplasms (Other)**				
Dabanovic et al. (2016) [144]	Montenegro (UMI)	**Intervention:** Dutasteride for male benign prostrate hyperplasia aged over 50<br>**Comparator:** Finasteride	None	• Dutasteride had ICER of US$1,245.68 per QALY relative to finasteride which is below threshold of US$1,350 per QALY (1-time GDP per capita)<br>•
Lv et al. (2017) [145]	China (UMI)	**Intervention:** Inpatient bed allocation strategies for cancer patients at an oncology centre: (i) allocation by programming to maximise bed occupancy accounting for constraints in hospital beds and chemotherapy protocols; (ii) other programming-based strategies with altered constraints (e.g., oncology centre is open on Saturday)<br>**Comparator:** (i) Standard allocation strategy (manual method); (ii) Comparison between strategies	None	• Allocation by programming increased average bed occupancy from 62.66% to 71.84% and reduced the number of unscheduled waiting patients per week from 30 to 13 relative to manual method<br>• Opening the oncology centre and allowing patients to start treatment on Saturday yielded the highest average bed occupancy (84.47%) and zero unscheduled waiting patient.
Mandrik et al. (2015) [146]	Ukraine (LMI)	**Intervention:** Rituximab plus fludarabine and cyclophosphamide (FCR) strategy for chronic lymphocytic leukaemia<br>**Comparator:** Fludarabine and cyclophosphamide (FC)	None	• FCR strategy had ICER of US$8,704 per QALY relative to FC for treatment naïve CLL patients which was cost-effective at threshold of 3-times GDP per capita (US$11,700)<br>• FCR strategy had ICER of US$11,056 per QALY relative to FC for refractory/relapsed CLL patients which was cost-effective at threshold of 3-times GDP per capita (US$11,700)
Udeh et al. (2016) [147]	Nigeria (LMI)	**Intervention:** Dutasteride plus tamsulosin for male benign prostrate hyperplasia aged over 50<br>**Comparator:** Dutasteride monotherapy	None	• Dutasteride plus tamsulosin had ICER of US$1,481.92 per QALY relative to dutasteride monotherapy which is below threshold of US$2,450 per QALY (1-time GDP per capita)
Wu et al. (2017b) [148]	China (UMI)	**Intervention:** RAS gene screening plus cetuximab and FOLFIRI (irinotecan, fluorouracil, leucovorin) for wild gene; and FOLFIRI alone for mutated gene in metastatic colorectal cancer<br>**Comparator:** FOLFIRI alone without gene screening	None	• Screening plus treatment strategy had ICER of US$14,049 per QALY relative to treatment alone under Patient Assistance Programme (PAP) which was cost-effective at threshold of 3-times GDP per capita (US$22,200 per QALY)<br>• Without PAP, ICER was US$27,145 per QALY which was not cost-effective
**Comparative Evaluation – Other non-communicable diseases**				
Pichon-Riviere et al. (2015b) [149]	29 countries in Latin America (LI/LMI/UMI)	**Intervention:** Oxytocin delivered by disposable auto-disable pre-filled injection device (BD Uniject SCF) – increases adherence – to prevent postpartum	Disadvantaged group: women	• Uniject delivery dominated (cost saving and health gain) standard delivery in 8 out of 30 countries<br>• In remaining 22 countries, the highest ICER of Uniject delivery was US$8,990 per QALY relative to standard delivery. In all

		haemorrhage for women giving birth in health facilities<br>**Comparator:** Oxytocin delivered by standard procedure (i.e., ampoules and syringes)		countries the ICER was below the threshold of 1-time GDP per capita

UMI: upper-middle-income country; LMI: lower-middle-income country; LI: low-income country
ALK: anaplastic lymphoma kinase; BMI: body mass index; CHD: coronary heart disease; CVD: cardiovascular disease; DALY: disability-adjusted life-year; EGFR: epidermal growth factor receptor; FCTC: Framework Convention on Tobacco Control; HPV: human papillomavirus; ICER: incremental cost-effectiveness ratio; IPCC: Intergovernmental Panel on Climate Change; IVA: isovaleric acidemia; MCD: multiple carboxylase deficiency; MI: myocardial infarction; MMA: methylmalonic acidemia; MSUD: maple syrup urine disease; OOP: out-of-pocket (payment); PA: propionic acidemia; PKU: phenylketonuria

Table C: Intersection between application area and equity approach

		Application area			
		Public health policy (n = 29)	Population screening (n = 30)	Healthcare provision - access (n = 41)	Healthcare provision – comparative (n = 49)
	Demonstrates equity focus	7 (24%)	29 (97%)	41 (100%)	11 (23%)
Equity approach (papers may be	Differential subgroup impact	3	1	4	-
	Disadvantaged group targeting	4	23	12	8
	Universal coverage of healthcare	-	23	41	4

References

1. Huang C, Moran AE, Coxson PG, Yang X, Liu F, Cao J, et al. Potential cardiovascular and total mortality benefits of air pollution control in urban China. Circulation. 2017;136(17):1575-84. doi: 10.1161/CIRCULATIONAHA.116.026487.
2. Li H, Nitivattananon V, Li P. Municipal solid waste management health risk assessment from air emissions for China by applying life cycle analysis. Waste management & research : the journal of the International Solid Wastes and Public Cleansing Association, ISWA. 2015;33(5):401-9. doi: https://dx.doi.org/10.1177/0734242X15580191.
3. Madaniyazi L, Nagashima T, Guo Y, Yu W, Tong S. Projecting fine particulate matter-related mortality in East China. Environmental science & technology. 2015;49(18):11141-50.
4. Xie Y, Zhao L, Xue J, Hu Q, Xu X, Wang H. A cooperative reduction model for regional air pollution control in China that considers adverse health effects and pollutant reduction costs. Science of the Total Environment. 2016;573:458-69. doi: 10.1016/j.scitotenv.2016.08.077.
5. Sa THd, Tainio M, Goodman A, Edwards P, Haines A, Gouveia N, et al. Health impact modelling of different travel patterns on physical activity, air pollution and road injuries for Sao Paulo, Brazil. Environment international. 2017;108:22-31. doi: https://dx.doi.org/10.1016/j.envint.2017.07.009.
6. Mushayabasa S. The Role of Optimal Intervention Strategies on Controlling Excessive Alcohol Drinking and Its Adverse Health Effects. Journal of Applied Mathematics. 2015;2015. doi: 10.1155/2015/238784.
7. Botteman M, Detzel P. Cost-effectiveness of partially hydrolyzed whey protein formula in the primary prevention of atopic dermatitis in high-risk urban infants in Southeast Asia. Annals of nutrition & metabolism. 2015;66 Suppl 1:26-32. doi: https://dx.doi.org/10.1159/000370222.
8. Dainelli L, Xu T, Li M, Zimmermann D, Fang H, Wu Y, et al. Cost-effectiveness of milk powder fortified with potassium to decrease blood pressure and prevent cardiovascular events among the adult population in China: a Markov model. BMJ open. 2017;7(9):e017136. doi: https://dx.doi.org/10.1136/bmjopen-2017-017136.
9. Vosti SA, Kagin J, Engle-Stone R, Brown KH. An Economic Optimization Model for Improving the Efficiency of Vitamin A Interventions: An Application to Young Children in Cameroon. Food and Nutrition Bulletin. 2015;36(3_suppl):S193-S207. doi: 10.1177/0379572115595889.
10. Barrientos-Gutierrez T, Zepeda-Tello R, Rodrigues ER, Colchero-Aragones A, Rojas-Martõnez R, Lazcano-Ponce E, et al. Expected population weight and diabetes impact of the 1-peso-per-litre tax to sugar sweetened beverages in Mexico. PLoS ONE. 2017;12(5). doi: 10.1371/journal.pone.0176336.
11. Erkoyun E, Sozmen K, Bennett K, Unal B, Boshuizen HC. Predicting the health impact of lowering salt consumption in Turkey using the DYNAMO health impact assessment tool. Public health. 2016;140:228-34. doi: https://dx.doi.org/10.1016/j.puhe.2016.04.014.
12. Marsh K, Moller J, Basarir H, Orfanos P, Detzel P. The Economic Impact of Lower Protein Infant Formula for the Children of Overweight and Obese Mothers. Nutrients. 2016;8(1). doi: https://dx.doi.org/10.3390/nu8010018.
13. Sanchez-Romero LM, Penko J, Coxson PG, Fernandez A, Mason A, Moran AE, et al. Projected Impact of Mexico's Sugar-Sweetened Beverage Tax Policy on Diabetes and Cardiovascular Disease: A Modeling Study. PLoS medicine. 2016;13(11):e1002158. doi: https://dx.doi.org/10.1371/journal.pmed.1002158.
14. Wang M, Moran AE, Liu J, Coxson PG, Penko J, Goldman L, et al. Projected impact of salt restriction on prevention of cardiovascular disease in China: a modeling study. PloS one. 2016;11(2):e0146820.
15. Watkins DA, Olson ZD, Verguet S, Nugent RA, Jamison DT. Cardiovascular disease and impoverishment averted due to a salt reduction policy in South Africa: an extended cost-effectiveness analysis. Health policy and planning. 2016;31(1):75-82. doi: https://dx.doi.org/10.1093/heapol/czv023.
16. Webb M, Fahimi S, Singh GM, Khatibzadeh S, Micha R, Powles J, et al. Cost effectiveness of a government supported policy strategy to decrease sodium intake: global analysis across 183 nations. BMJ (Clinical research ed). 2017;356:i6699. doi: https://dx.doi.org/10.1136/bmj.i6699.
17. Wilcox ML, Mason H, Fouad FM, Rastam S, Al Ali R, Page TF, et al. Cost-effectiveness analysis of salt reduction policies to reduce coronary heart disease in Syria, 2010–2020. International journal of public health. 2015;60(1):23-30.

18. Springmann M, Mason-D'Croz D, Robinson S, Garnett T, Godfray HCJ, Gollin D, et al. Global and regional health effects of future food production under climate change: A modelling study. The Lancet. 2016;387(10031):1937-46. doi: 10.1016/S0140-6736(15)01156-3.
19. Borracci RA, Mulassi AH. Tobacco use during adolescence may predict smoking during adulthood: Simulation-based research. Archivos Argentinos de Pediatria. 2015;113(2):106-13. doi: 10.5546/aap.2015.eng.106.
20. Fleischer NL, Thrasher JF, Reynales-Shigematsu LM, Cummings KM, Meza R, Zhang Y, et al. Mexico SimSmoke: how changes in tobacco control policies would impact smoking prevalence and smoking attributable deaths in Mexico. Global Public Health. 2017;12(7):830-45. doi: 10.1080/17441692.2015.1123749.
21. Levy DT, Fouad H, Levy J, Dragomir AD, Awa FE. Application of the abridged simsmoke model to four eastern Mediterranean countries. Tobacco Control. 2016;25(4):413-21. doi: 10.1136/tobaccocontrol-2015-052334.
22. Levy D, Rodríguez-Buño RL, Hu T-W, Moran AE. The potential effects of tobacco control in China: projections from the China SimSmoke simulation model. BMJ : British Medical Journal. 2014;348:g1134. doi: 10.1136/bmj.g1134.
23. Ngalesoni F, Ruhago G, Mayige M, Oliveira TC, Robberstad B, Norheim OF, et al. Cost-effectiveness analysis of population-based tobacco control strategies in the prevention of cardiovascular diseases in Tanzania. PLoS ONE. 2017;12(8). doi: 10.1371/journal.pone.0182113.
24. Reynales-Shigematsu LM, Fleischer NL, Thrasher JF, Zhang Y, Meza R, Cummings KM, et al. Effects of tobacco control policies on smoking prevalence and tobacco-attributable deaths in Mexico: the SimSmoke model. Revista panamericana de salud publica = Pan American journal of public health. 2015;38(4):316-25.
25. Szklo AS, Yuan Z, Levy D. Update and extension of the Brazil SimSmoke model to estimate the health impact of cigarette smoking by pregnant women in Brazil. Cadernos de saude publica. 2017;33(12):e00207416. doi: https://dx.doi.org/10.1590/0102-311X00207416.
26. Verguet S, Gauvreau CL, Mishra S, MacLennan M, Murphy SM, Brouwer ED, et al. The consequences of tobacco tax on household health and finances in rich and poor smokers in China: an extended cost-effectiveness analysis. The Lancet Global Health. 2015;3(4):e206-e16.
27. Stacey N, Summan A, Tugendhaft A, Laxminarayan R, Hofman K. Simulating the impact of excise taxation for disease prevention in low-income and middle-income countries: an application to South Africa. BMJ global health. 2018;3(1):e000568. doi: https://dx.doi.org/10.1136/bmjgh-2017-000568.
28. Islek D, Sozmen K, Unal B, Guzman-Castillo M, Vaartjes I, Critchley J, et al. Estimating the potential contribution of stroke treatments and preventative policies to reduce the stroke and ischemic heart disease mortality in Turkey up to 2032: a modelling study. BMC public health. 2016;16:46. doi: https://dx.doi.org/10.1186/s12889-015-2655-8.
29. Sozmen K, Unal B, Capewell S, Critchley J, O'Flaherty M. Estimating diabetes prevalence in Turkey in 2025 with and without possible interventions to reduce obesity and smoking prevalence, using a modelling approach. International Journal of Public Health. 2015;60:S13-S21. doi: 10.1007/s00038-014-0622-2. PubMed PMID: WOS:000347404600003.
30. Dukpa W, Teerawattananon Y, Rattanavipapong W, Srinonprasert V, Tongsri W, Kingkaew P, et al. Is diabetes and hypertension screening worthwhile in resource-limited settings? An economic evaluation based on a pilot of a Package of Essential Non-communicable disease interventions in Bhutan. Health Policy and Planning. 2015;30(8):1032-43. doi: 10.1093/heapol/czu106.
31. Gaziano T, Abrahams-Gessel S, Surka S, Sy S, Pandya A, Denman CA, et al. Cardiovascular Disease Screening By Community Health Workers Can Be Cost-Effective In Low-Resource Countries. Health affairs (Project Hope). 2015;34(9):1538-45. doi: https://dx.doi.org/10.1377/hlthaff.2015.0349.
32. Basu S, Millett C, Vijan S, Hayward RA, Kinra S, Ahuja R, et al. The Health System and Population Health Implications of Large-Scale Diabetes Screening in India: A Microsimulation Model of Alternative Approaches. PLoS Medicine. 2015;12(5). doi: 10.1371/journal.pmed.1001827.
33. Teimouri M, Rooein D, Ebrahimi M, editors. Optimization of budgeting type 2 diabetes through modeling disease burden2015.
34. Toscano CM, Zhuo X, Imai K, Duncan BB, Polanczyk CA, Zhang P, et al. Cost-effectiveness of a national population-based screening program for type 2 diabetes: the Brazil experience. Diabetology & metabolic syndrome. 2015;7:95. doi: https://dx.doi.org/10.1186/s13098-015-0090-8.

35.Thiboonboon K, Leelahavarong P, Wattanasirichaigoon D, Vatanavicharn N, Wasant P, Shotelersuk V, et al. An Economic Evaluation of Neonatal Screening for Inborn Errors of Metabolism Using Tandem Mass Spectrometry in Thailand. PloS one. 2015;10(8):e0134782. doi: https://dx.doi.org/10.1371/journal.pone.0134782.
36.Si L, Winzenberg T, Jiang Q, Palmer A. Screening for and treatment of osteoporosis: construction and validation of a state-transition microsimulation cost-effectiveness model. Osteoporosis International. 2015;26(5):1477-89.
37.Haghighat S, Akbari ME, Yavari P, Javanbakht M, Ghaffari S. Cost-Effectiveness of Three Rounds of Mammography Breast Cancer Screening in Iranian Women. Iranian journal of cancer prevention. 2016;9(1):e5443. doi: https://dx.doi.org/10.17795/ijcp-5443.
38.Nguyen CP, Adang EMM. Cost-effectiveness of breast cancer screening using mammography in Vietnamese women. Plos One. 2018;13(3). doi: 10.1371/journal.pone.0194996. PubMed PMID: WOS:000428351800075.
39.Ulloa-Pérez E, Mohar-Betancourt A, Reynoso-Noverón N. Estimation of the Cost-Effectiveness of Breast Cancer Screening Using Mammography in Mexico Through a Simulation. Revista de investigacion clinica; organo del Hospital de Enfermedades de la Nutricion. 2016;68(4):184-91.
40.Wagh B, Chaluvarayaswamy R, Pal D. Assessment of Adaptive Breast Cancer Screening Policies for Improved Mortality Reduction in Low to Middle Income Countries. Asian Pacific journal of cancer prevention : APJCP. 2017;18(9):2375-80.
41.Zehtab N, Jafari M, Barooni M, Nakhaee N, Goudarzi R, Larry Zadeh MH. Cost-Effectiveness Analysis of Breast Cancer Screening in Rural Iran. Asian Pacific journal of cancer prevention : APJCP. 2016;17(2):609-14.
42.Akhavan-Tabatabaei R, Sanchez DM, Yeung TG. A Markov Decision Process Model for Cervical Cancer Screening Policies in Colombia. Medical decision making : an international journal of the Society for Medical Decision Making. 2017;37(2):196-211. doi: https://dx.doi.org/10.1177/0272989X16670622.
43.Campos NG, Castle PE, Wright TC, Jr., Kim JJ. Cervical cancer screening in low-resource settings: A cost-effectiveness framework for valuing tradeoffs between test performance and program coverage. International journal of cancer. 2015;137(9):2208-19. doi: https://dx.doi.org/10.1002/ijc.29594.
44.Campos NG, Maza M, Alfaro K, Gage JC, Castle PE, Felix JC, et al. The comparative and cost-effectiveness of HPV-based cervical cancer screening algorithms in El Salvador. International journal of cancer. 2015;137(4):893-902. doi: https://dx.doi.org/10.1002/ijc.29438.
45.Campos NG, Tsu V, Jeronimo J, Mvundura M, Kim JJ. Estimating the value of point-of-care HPV testing in three low- and middle-income countries: A modeling study. BMC Cancer. 2017;17(1). doi: 10.1186/s12885-017-3786-3.
46.Campos NG, Tsu V, Jeronimo J, Mvundura M, Lee K, Kim JJ. To expand coverage, or increase frequency: Quantifying the tradeoffs between equity and efficiency facing cervical cancer screening programs in low-resource settings. International journal of cancer. 2017;140(6):1293-305. doi: https://dx.doi.org/10.1002/ijc.30551.
47.Campos NG, Tsu V, Jeronimo J, Njama-Meya D, Mvundura M, Kim JJ. Cost-effectiveness of an HPV self-collection campaign in Uganda: comparing models for delivery of cervical cancer screening in a low-income setting. Health policy and planning. 2017;32(7):956-68. doi: https://dx.doi.org/10.1093/heapol/czw182.
48.Guerrero AM, Genuino AJ, Santillan M, Praditsitthikorn N, Chantarastapornchit V, Teerawattananon Y, et al. A cost-utility analysis of cervical cancer screening and human papillomavirus vaccination in the Philippines. BMC Public Health. 2015;15(1). doi: 10.1186/s12889-015-2046-1.
49.Levin CE, Sharma M, Olson Z, Verguet S, Shi J-F, Wang S-M, et al. An extended cost-effectiveness analysis of publicly financed HPV vaccination to prevent cervical cancer in China. Vaccine. 2015;33(24):2830-41. doi: https://dx.doi.org/10.1016/j.vaccine.2015.02.052.
50.Mo X, Gai Tobe R, Wang L, Liu X, Wu B, Luo H, et al. Cost-effectiveness analysis of different types of human papillomavirus vaccination combined with a cervical cancer screening program in mainland China. BMC infectious diseases. 2017;17(1):502. doi: https://dx.doi.org/10.1186/s12879-017-2592-5.

51.Setiawan D, Dolk FC, Suwantika AA, Westra TA, Wilschut JC, Postma MJ. Cost-Utility Analysis of Human Papillomavirus Vaccination and Cervical Screening on Cervical Cancer Patient in Indonesia. Value in health regional issues. 2016;9:84-92. doi: https://dx.doi.org/10.1016/j.vhri.2015.10.010.
52.Termrungruanglert W, Khemapech N, Tantitamit T, Sangrajrang S, Havanond P, Laowahutanont P. Cost-effectiveness analysis study of HPV testing as a primary cervical cancer screening in Thailand. Gynecologic Oncology Reports. 2017;22:58-63. doi: 10.1016/j.gore.2017.09.007.
53.Sheehan DF, Criss SD, Gazelle GS, Pandharipande PV, Kong CY. Evaluating lung cancer screening in China: Implications for eligibility criteria design from a microsimulation modeling approach. PLoS ONE. 2017;12(3). doi: 10.1371/journal.pone.0173119.
54.Kuznik A, Habib AG, Munube D, Lamorde M. Newborn screening and prophylactic interventions for sickle cell disease in 47 countries in sub-Saharan Africa: A cost-effectiveness analysis. BMC Health Services Research. 2016;16(1). doi: 10.1186/s12913-016-1572-6.
55.Tobe RG, Martin GR, Li F, Moriichi A, Wu B, Mori R. Cost-effectiveness analysis of neonatal screening of critical congenital heart defects in China. Medicine. 2017;96(46):e8683. doi: https://dx.doi.org/10.1097/MD.0000000000008683.
56.John D, Parikh R. Cost-effectiveness and cost utility of community screening for glaucoma in urban India. Public health. 2017;148:37-48. doi: https://dx.doi.org/10.1016/j.puhe.2017.02.016.
57.Rothschild MI, Russ R, Brennan KA, Williams CJ, Berrones D, Patel B, et al. The Economic Model of Retinopathy of Prematurity (EcROP) Screening and Treatment: Mexico and the United States. American journal of ophthalmology. 2016;168:110-21. doi: https://dx.doi.org/10.1016/j.ajo.2016.04.014.
58.Vinekar A, Mangalesh S, Jayadev C, Gilbert C, Dogra M, Shetty B. Impact of expansion of telemedicine screening for retinopathy of prematurity in India. Indian journal of ophthalmology. 2017;65(5):390-5. doi: https://dx.doi.org/10.4103/ijo.IJO_211_17.
59.Wong L-Y, Espinoza F, Alvarez KM, Molter D, Saunders JE. Otoacoustic Emissions in Rural Nicaragua: Cost Analysis and Implications for Newborn Hearing Screening. Otolaryngology--head and neck surgery : official journal of American Academy of Otolaryngology-Head and Neck Surgery. 2017;156(5):877-85. doi: https://dx.doi.org/10.1177/0194599817696306.
60.Basu S, Bendavid E, Sood N. Health and Economic Implications of National Treatment Coverage for Cardiovascular Disease in India: Cost-Effectiveness Analysis. Circulation: Cardiovascular Quality and Outcomes. 2015;8(6):541-51. doi: 10.1161/CIRCOUTCOMES.115.001994.
61.Chen T, Yu D, Cornelius V, Qin R, Cai Y, Jiang Z, et al. Potential health impact and cost-effectiveness of drug therapy for prehypertension. International Journal of Cardiology. 2017;240:403-8. doi: http://dx.doi.org/10.1016/j.ijcard.2017.05.003.
62.Gu D, He J, Coxson PG, Rasmussen PW, Huang C, Thanataveerat A, et al. The Cost-Effectiveness of Low-Cost Essential Antihypertensive Medicines for Hypertension Control in China: A Modelling Study. PLoS medicine. 2015;12(8):e1001860. doi: https://dx.doi.org/10.1371/journal.pmed.1001860.
63.Ngalesoni FN, Ruhago GM, Mori AT, Robberstad B, Norheim OF. Cost-effectiveness of medical primary prevention strategies to reduce absolute risk of cardiovascular disease in Tanzania: a Markov modelling study. BMC health services research. 2016;16:185. doi: https://dx.doi.org/10.1186/s12913-016-1409-3.
64.Phisalprapa P, Supakankunti S, Charatcharoenwitthaya P, Apisarnthanarak P, Charoensak A, Washirasaksiri C, et al. Cost-effectiveness analysis of ultrasonography screening for nonalcoholic fatty liver disease in metabolic syndrome patients. Medicine. 2017;96(17):e6585. doi: https://dx.doi.org/10.1097/MD.0000000000006585.
65.Thongsri W, Bussabawalai T, Leelahavarong P, Wanitkun S, Durongpisitkul K, Chaikledkaew U, et al. Cost-utility and budget impact analysis of drug treatments in pulmonary arterial hypertension associated with congenital heart diseases in Thailand. Expert review of pharmacoeconomics & outcomes research. 2016;16(4):525-36. doi: https://dx.doi.org/10.1586/14737167.2016.1120672.
66.Rodriguez-Martinez CE, Sossa-Briceno MP, Castro-Rodriguez JA. A cost-effectiveness threshold analysis of a multidisciplinary structured educational intervention in pediatric asthma. The Journal of asthma : official journal of the Association for the Care of Asthma. 2018;55(5):561-70. doi: https://dx.doi.org/10.1080/02770903.2017.1348512.
67.Afiatin, Khoe LC, Kristin E, Masytoh LS, Herlinawaty E, Werayingyong P, et al. Economic evaluation of policy options for dialysis in end-stage renal disease patients under the universal health

coverage in Indonesia. PloS one. 2017;12(5):e0177436. doi: https://dx.doi.org/10.1371/journal.pone.0177436.
68.Cardenas MK, Mirelman AJ, Galvin CJ, Lazo-Porras M, Pinto M, Miranda JJ, et al. The cost of illness attributable to diabetic foot and cost-effectiveness of secondary prevention in Peru. BMC health services research. 2015;15:483. doi: https://dx.doi.org/10.1186/s12913-015-1141-4.
69.Home P, Baik SH, Gálvez GG, Malek R, Nikolajsen A. An analysis of the cost-effectiveness of starting insulin detemir in insulin-naive people with type 2 diabetes. Journal of medical economics. 2015;18(3):230-40.
70.Mash R, Kroukamp R, Gaziano T, Levitt N. Cost-effectiveness of a diabetes group education program delivered by health promoters with a guiding style in underserved communities in Cape Town, South Africa. Patient education and counseling. 2015;98(5):622-6. doi: https://dx.doi.org/10.1016/j.pec.2015.01.005.
71.Vetrini D, Kiire CA, Burgess PI, Harding SP, Kayange PC, Kalua K, et al. Incremental cost-effectiveness of screening and laser treatment for diabetic retinopathy and macular edema in Malawi. PloS one. 2018;13(1):e0190742. doi: https://dx.doi.org/10.1371/journal.pone.0190742.
72.Wu B, Wan X, Ma J. Cost-effectiveness of prevention and management of diabetic foot ulcer and amputation in a health resource-limited setting. Journal of Diabetes. 2018;10(4):320-7. doi: http://dx.doi.org/10.1111/1753-0407.12612.
73.Wu B, Zhang S, Lin H, Mou S. Prevention of renal failure in Chinese patients with newly diagnosed type 2 diabetes: A cost-effectiveness analysis. Journal of diabetes investigation. 2018;9(1):152-61. doi: https://dx.doi.org/10.1111/jdi.12653.
74.Anh NQ, Linh BN, Ha NT, Phanthunane P, Huong NT. Schizophrenia interventions in Vietnam: Primary results from a cost-effectiveness study. Global Public Health. 2015;10:S21-S39. doi: 10.1080/17441692.2014.986158.
75.Johansson KA, Strand KB, Fekadu A, Chisholm D. Health Gains and Financial Protection Provided by the Ethiopian Mental Health Strategy: an Extended Cost-Effectiveness Analysis. Health policy and planning. 2017;32(3):376-83. doi: https://dx.doi.org/10.1093/heapol/czw134.
76.Kitwitee P, Unnwongse K, Srikijvilaikul T, Yadee T, Limwattananon C. Cost-Utility of Video-Electroencephalography Monitoring Followed by Surgery in Adults with Drug-Resistant Focal Epilepsy in Thailand. World neurosurgery. 2017;98:750-60.e3. doi: https://dx.doi.org/10.1016/j.wneu.2016.11.041.
77.Linde M, Steiner TJ, Chisholm D. Cost-effectiveness analysis of interventions for migraine in four low- and middle-income countries. The journal of headache and pain. 2015;16:15. doi: https://dx.doi.org/10.1186/s10194-015-0496-6.
78.Megiddo I, Colson A, Chisholm D, Dua T, Nandi A, Laxminarayan R. Health and economic benefits of public financing of epilepsy treatment in India: An agent-based simulation model. Epilepsia. 2016;57(3):464-74. doi: https://dx.doi.org/10.1111/epi.13294.
79.Strand KB, Chisholm D, Fekadu A, Johansson KA. Scaling-up essential neuropsychiatric services in Ethiopia: a cost-effectiveness analysis. Health policy and planning. 2016;31(4):504-13. doi: https://dx.doi.org/10.1093/heapol/czv093.
80.Eeson G, Birabwa-Male D, Pennington M, Blair GK. Costs and cost-effectiveness of pediatric inguinal hernia repair in Uganda. World Journal of Surgery. 2015;39(2):343-9. doi: 10.1007/s00268-014-2818-2.
81.Ansaripour A, Uyl-de Groot CA, Redekop WK. Adjuvant trastuzumab therapy for early HER2-positive breast cancer in Iran: a cost-effectiveness and scenario analysis for an optimal treatment strategy. PharmacoEconomics. 2018;36(1):91-103.
82.Pichon-Riviere A, Garay OU, Augustovski F, Vallejos C, Huayanay L, Bueno MdPN, et al. Implications of global pricing policies on access to innovative drugs: The case of trastuzumab in seven Latin American countries. International Journal of Technology Assessment in Health Care. 2015;31(1-2):2-11. doi: https://dx.doi.org/10.1017/S0266462315000094.
83.Lu Y, Cheng J, Lin Z, Chen Y, Xuan J. Pharmacoeconomic analysis for pemetrexed as a maintenance therapy for NSCLC patients with patient assistance program in China. Journal of medical economics. 2018;21(1):60-5. doi: https://dx.doi.org/10.1080/13696998.2017.1373654.
84.Shi Q, Hu S, Furnback WE, Guzauskas GF, Shen J, Wang BC. The cost-effectiveness of a NSCLC patient assistance program for pemetrexed maintenance therapy in People's Republic of China.

ClinicoEconomics and outcomes research : CEOR. 2017;9:99-106. doi: https://dx.doi.org/10.2147/CEOR.S119818.
85.Thongprasert S, Permsuwan U. Crizotinib treatment for advanced non-small-cell lung cancer patients: a budget impact analysis based in Thailand. Current Medical Research and Opinion. 2017;33(5):955-61. doi: http://dx.doi.org/10.1080/03007995.2017.1297929.
86.Sheng G, Chen S, Dong C, Zhang R, Miao M, Wu D, et al. Societal implications of medical insurance coverage for imatinib as first-line treatment of chronic myeloid leukemia in China: a cost-effectiveness analysis. Journal of Medical Economics. 2017;20(4):371-81. doi: 10.1080/13696998.2016.1271336.
87.Wu B, Liu M, Li T, Lin H, Zhong H. An economic analysis of high-dose imatinib, dasatinib, and nilotinib for imatinib-resistant chronic phase chronic myeloid leukemia in China: A CHEERS-compliant article. Medicine. 2017;96(29):e7445. doi: https://dx.doi.org/10.1097/MD.0000000000007445.
88.Shrime MG, Sekidde S, Linden A, Cohen JL, Weinstein MC, Salomon JA. Sustainable Development in Surgery: The Health, Poverty, and Equity Impacts of Charitable Surgery in Uganda. PloS one. 2016;11(12):e0168867. doi: https://dx.doi.org/10.1371/journal.pone.0168867.
89.Zhang P, Yang Y, Wen F, He X, Tang R, Du Z, et al. Cost-effectiveness of sorafenib as a first-line treatment for advanced hepatocellular carcinoma. European journal of gastroenterology & hepatology. 2015;27(7):853-9. doi: https://dx.doi.org/10.1097/MEG.0000000000000373.
90.Zhou K, Wen F, Zhang P, Zhou J, Chen H, Zheng H, et al. Efficacy and cost-effectiveness of second-line chemotherapy in elderly patients with advanced gastric cancer. Clinical & translational oncology : official publication of the Federation of Spanish Oncology Societies and of the National Cancer Institute of Mexico. 2017;19(9):1117-24. doi: https://dx.doi.org/10.1007/s12094-017-1647-9.
91.Moran D, Shrime MG, Nang S, Vycheth I, Vuthy D, Hong R, et al. Cost-Effectiveness of Craniotomy for Epidural Hematomas at a Major Government Hospital in Cambodia. World journal of surgery. 2017;41(9):2215-23. doi: https://dx.doi.org/10.1007/s00268-017-4022-7.
92.Semwanga AR, Nakubulwa S, Adam T. Applying a system dynamics modelling approach to explore policy options for improving neonatal health in Uganda. Health Research Policy and Systems. 2016;14(1). doi: 10.1186/s12961-016-0101-8.
93.Verguet S, Olson ZD, Babigumira JB, Desalegn D, Johansson KA, Kruk ME, et al. Health gains and financial risk protection afforded by public financing of selected interventions in Ethiopia: an extended cost-effectiveness analysis. The Lancet Global health. 2015;3(5):e288-96. doi: https://dx.doi.org/10.1016/S2214-109X(14)70346-8.
94.Watkins DA, Mvundura M, Nordet P, Mayosi BM. A cost-effectiveness analysis of a program to control rheumatic fever and rheumatic heart disease in Pinar del Rio, Cuba. PloS one. 2015;10(3):e0121363. doi: https://dx.doi.org/10.1371/journal.pone.0121363.
95.Watkins D, Lubinga SJ, Mayosi B, Babigumira JB. A Cost-Effectiveness Tool to Guide the Prioritization of Interventions for Rheumatic Fever and Rheumatic Heart Disease Control in African Nations. PLoS neglected tropical diseases. 2016;10(8):e0004860. doi: https://dx.doi.org/10.1371/journal.pntd.0004860.
96.Emmett SD, Tucci DL, Smith M, Macharia IM, Ndegwa SN, Nakku D, et al. GDP matters: cost effectiveness of cochlear implantation and deaf education in Sub-Saharan Africa. Otology & Neurotology. 2015;36(8):1357-65.
97.Guedes RAP, Guedes VMP, Gomes CEdM, Chaoubah A. Maximizing cost-effectiveness by adjusting treatment strategy according to glaucoma severity. Medicine. 2016;95(52):e5745. doi: https://dx.doi.org/10.1097/MD.0000000000005745.
98.Wu B, Yang Z, Tobe RG, Wang Y. Medical therapy for preventing recurrent endometriosis after conservative surgery: a cost-effectiveness analysis. Bjog-an International Journal of Obstetrics and Gynaecology. 2018;125(4):469-77. doi: 10.1111/1471-0528.14786. PubMed PMID: WOS:000425127900011.
99.Kessler J, Ruggles K, Patel A, Nucifora K, Li L, Roberts MS, et al. Targeting an Alcohol Intervention Cost-Effectively to Persons Living with HIV/AIDS in East Africa. Alcoholism: Clinical and Experimental Research. 2015;39(11):2179-88. doi: 10.1111/acer.12890.
100. Tosanguan J, Chaiyakunapruk N. Cost-effectiveness analysis of clinical smoking cessation interventions in Thailand. Addiction. 2016;111(2):340-50. doi: 10.1111/add.13166. PubMed PMID: WOS:000368940500019.

101. Basu S, Yudkin JS, Sussman JB, Millett C, Hayward RA. Alternative Strategies to Achieve Cardiovascular Mortality Goals in China and India: A Microsimulation of Target- Versus Risk-Based Blood Pressure Treatment. Circulation. 2016;133(9):840-8. doi: https://dx.doi.org/10.1161/CIRCULATIONAHA.115.019985.
102. Borissov B, Urbich M, Georgieva B, Tsenov S, Villa G. Cost-effectiveness of evolocumab in treatment of heterozygous familial hypercholesterolaemia in Bulgaria: measuring health benefit by effectively treated patient-years. Journal of market access & health policy. 2017;5(1):1412753. doi: https://dx.doi.org/10.1080/20016689.2017.1412753.
103. Cui M, Tu CC, Chen EZ, Wang XL, Tan SC, Chen C. A Cost-Effectiveness Analysis of Clopidogrel for Patients with Non-ST-Segment Elevation Acute Coronary Syndrome in China. Advances in therapy. 2016;33(9):1600-11. doi: https://dx.doi.org/10.1007/s12325-016-0375-9.
104. Giorgi MA, Caroli C, Giglio ND, Micone P, Aiello E, Vulcano C, et al. Estimation of the cost-effectiveness of apixaban versus vitamin K antagonists in the management of atrial fibrillation in Argentina. Health economics review. 2015;5(1):52. doi: https://dx.doi.org/10.1186/s13561-015-0052-8.
105. Konfino J, Fernandez A, Penko J, Mason A, Martinez E, Coxson P, et al. Comparing Strategies for Lipid Lowering in Argentina: An Analysis from the CVD Policy Model-Argentina. Journal of general internal medicine. 2017;32(5):524-33. doi: https://dx.doi.org/10.1007/s11606-016-3907-8.
106. Permsuwan U, Chaiyakunapruk N, Nathisuwan S, Sukonthasarn A. Cost-Effectiveness Analysis of Fondaparinux vs Enoxaparin in Non-ST Elevation Acute Coronary Syndrome in Thailand. Heart, lung & circulation. 2015;24(9):860-8. doi: https://dx.doi.org/10.1016/j.hlc.2015.02.018.
107. Ribeiro RA, Duncan BB, Ziegelmann PK, Stella SF, Vieira JLdC, Restelatto LMF, et al. Cost-effectiveness of high, moderate and low-dose statins in the prevention of vascular events in the Brazilian public health system. Arquivos brasileiros de cardiologia. 2015;104(1):32-44. doi: https://dx.doi.org/10.5935/abc.20140173.
108. Xie X, He T, Kang J, Siscovick DS, Li Y, Pagan JA. Cost-effectiveness analysis of intensive hypertension control in China. Preventive medicine. 2018;111:110-4. doi: https://dx.doi.org/10.1016/j.ypmed.2018.02.033.
109. Xuan JW, Huang M, Lu YJ, Tao LB. Economic Evaluation of Safflower Yellow Injection for the Treatment of Patients with Stable Angina Pectoris in China: A Cost-Effectiveness Analysis. Journal of Alternative and Complementary Medicine. 2018. doi: 10.1089/acm.2017.0284. PubMed PMID: WOS:000424841800001.
110. Rodriguez-Martinez CE, Nino G, Castro-Rodriguez JA. Cost-utility analysis of daily versus intermittent inhaled corticosteroids in mild-persistent asthma. Pediatric pulmonology. 2015;50(8):735-46.
111. Rodriguez-Martinez CE, Sossa-Briceno MP, Castro-Rodriguez JA. Cost–utility analysis of once-daily versus twice-daily inhaled corticosteroid dosing for maintenance treatment of asthma in pediatric patients. Journal of Asthma. 2016;53(5):538-45.
112. Suzuki C, Lopes da Silva N, Kumar P, Pathak P, Ong SH. Cost-effectiveness of omalizumab add-on to standard-of-care therapy in patients with uncontrolled severe allergic asthma in a Brazilian healthcare setting. Journal of medical economics. 2017;20(8):832-9. doi: https://dx.doi.org/10.1080/13696998.2017.1333513.
113. Balderas-Penã LMA, Sat-Munõz D, Ramírez-Conchas RE, Alvarado-Iñiguez MR, Garciá-De-Alba-Garciá JE, Cruz-Corona E, et al. Descriptive, Longitudinal Study Results Applied to Statistical Models to Assess the Impact of Early Microbiological Cultures on the Economic Burden of Treatment for Infected Diabetic Foot Ulcers at a Mexican Public Health Facility. Ostomy Wound Management. 2016;62(12):14-28.
114. Carlos F, Espejel L, Novick D, López R, Flores D. Duloxetine for the treatment of painful diabetic peripheral neuropathy in Venezuela: economic evaluation. Medwave. 2015;15(8):e6265. doi: 10.5867/medwave.2015.08.6265.
115. Deng J, Gu S, Shao H, Dong H, Zou D, Shi L. Cost-effectiveness analysis of exenatide twice daily (BID) vs insulin glargine once daily (QD) as add-on therapy in Chinese patients with Type 2 diabetes mellitus inadequately controlled by oral therapies. Journal of medical economics. 2015;18(11):974-89.

116. Gomez AM, Alfonso-Cristancho R, Orozco JJ, Lynch PM, Prieto D, Saunders R, et al. Clinical and economic benefits of integrated pump/CGM technology therapy in patients with type 1 diabetes in Colombia. Endocrinologia y Nutricion. 2016;63(9):466-74. doi: 10.1016/j.endonu.2016.05.011.
117. Gu S, Deng J, Shi L, Mu Y, Dong H. Cost-effectiveness of saxagliptin vs glimepiride as a second-line therapy added to metformin in Type 2 diabetes in China. Journal of Medical Economics. 2015;18(10):808-20. doi: 10.3111/13696998.2015.1049542.
118. Gu S, Zeng Y, Yu D, Hu X, Dong H. Cost-effectiveness of saxagliptin versus acarbose as second-line therapy in type 2 diabetes in China. PloS one. 2016;11(11):e0167190.
119. Gupta V, Baabbad R, Hammerby E, Nikolajsen A, Shafie AA. An analysis of the cost-effectiveness of switching from biphasic human insulin 30, insulin glargine, or neutral protamine Hagedorn to biphasic insulin aspart 30 in people with type 2 diabetes. Journal of medical economics. 2015;18(4):263-72.
120. Permsuwan U, Chaiyakunapruk N, Dilokthornsakul P, Thavorn K, Saokaew S. Long-Term Cost-Effectiveness of Insulin Glargine Versus Neutral Protamine Hagedorn Insulin for Type 2 Diabetes in Thailand. Applied Health Economics and Health Policy. 2016;14(3):281-92. doi: 10.1007/s40258-016-0228-3. PubMed PMID: WOS:000376126500004.
121. Shao H, Zhai SD, Zou DJ, Mir MU, Zawadzki NK, Shi Q, et al. Cost-effectiveness analysis of dapagliflozin versus glimepiride as monotherapy in a Chinese population with type 2 diabetes mellitus. Current Medical Research and Opinion. 2017;33(2):359-69. doi: 10.1080/03007995.2016.1257978. PubMed PMID: WOS:000392670300022.
122. Xuan JW, Song RL, Xu GX, Lu WQ, Lu YJ, Liu Z. Modeling the cost-effectiveness of ilaprazole versus omeprazole for the treatment of newly diagnosed duodenal ulcer patients in China. Journal of medical economics. 2016;19(11):1056-60.
123. Lubinga SJ, Mutamba BB, Nganizi A, Babigumira JB. A Cost-effectiveness Analysis of Antipsychotics for Treatment of Schizophrenia in Uganda. Applied health economics and health policy. 2015;13(5):493-506. doi: https://dx.doi.org/10.1007/s40258-015-0176-3.
124. Bamrungsawad N, Chaiyakunapruk N, Upakdee N, Pratoomsoot C, Sruamsiri R, Dilokthornsakul P. Cost-utility analysis of intravenous immunoglobulin for the treatment of steroid-refractory dermatomyositis in Thailand. PharmacoEconomics. 2015;33(5):521-31. doi: https://dx.doi.org/10.1007/s40273-015-0269-8.
125. Wu B, Song Y, Leng L, Bucala R, Lu L-j. Treatment of moderate rheumatoid arthritis with different strategies in a health resource-limited setting: a cost-effectiveness analysis in the era of biosimilars. Clinical and experimental rheumatology. 2015;33(1):20-6.
126. Yan X, Gu X, Zhou L, Lin H, Wu B. Cost Effectiveness of Apixaban and Enoxaparin for the Prevention of Venous Thromboembolism After Total Knee Replacement in China. Clinical drug investigation. 2016;36(12):1001-10.
127. Yan XY, Gu XH, Xu ZX, Lin HW, Wu B. Cost-Effectiveness of Different Strategies for the Prevention of Venous Thromboembolism After Total Hip Replacement in China. Advances in Therapy. 2017;34(2):466-80. doi: 10.1007/s12325-016-0460-0. PubMed PMID: WOS:000395443300010.
128. Bargallo-Rocha JE, Lara-Medina F, Perez-Sanchez V, Vazquez-Romo R, Villarreal-Garza C, Martinez-Said H, et al. Cost-effectiveness of the 21-gene breast cancer assay in Mexico. Advances in therapy. 2015;32(3):239-53. doi: https://dx.doi.org/10.1007/s12325-015-0190-8.
129. Diaby V, Ali AA, Williams KJ, Ezendu K, Soto-Perez-de-Celis E, Chavarri-Guerra Y, et al. Economic evaluation of sequencing strategies in HER2-positive metastatic breast cancer in Mexico: a contrast between public and private payer perspectives. Breast Cancer Research and Treatment. 2017;166(3):951-63. doi: 10.1007/s10549-017-4473-4.
130. Hatam N, Ahmadloo N, Vazirzadeh M, Jafari A, Askarian M. Cost-Effectiveness of Intensive Vs. Standard Follow-Up Models for Patients with Breast Cancer in Shiraz, Iran. Asian Pacific journal of cancer prevention : APJCP. 2016;17(12):5309-14.
131. Khan AJ, Rafique R, Zafar W, Shah C, Haffty BG, Vicini F, et al. Nation-Scale Adoption of Shorter Breast Radiation Therapy Schedules Can Increase Survival in Resource Constrained Economies: Results From a Markov Chain Analysis. International journal of radiation oncology, biology, physics. 2017;97(2):287-95. doi: https://dx.doi.org/10.1016/j.ijrobp.2016.10.002.
132. Rodriguez-Aguilar R, Marmolejo-Saucedo JA, Tavera-Martinez S. Financial risk of increasing the follow-up period of breast cancer treatment currently covered by the Social Protection System in

Health in Mexico. Cost effectiveness and resource allocation : C/E. 2018;16:9. doi: https://dx.doi.org/10.1186/s12962-018-0094-y.
133. Arrieta O, Anaya P, Morales-Oyarvide V, Ramirez-Tirado LA, Polanco AC. Cost-effectiveness analysis of EGFR mutation testing in patients with non-small cell lung cancer (NSCLC) with gefitinib or carboplatin-paclitaxel. European Journal of Health Economics. 2016;17(7):855-63. doi: http://dx.doi.org/10.1007/s10198-015-0726-5.
134. Limwattananon C, Limwattananon S, Waleekhachonloet O, Rattanachotphanit T. Cost-effectiveness analysis of policy options on first-line treatments for advanced, non-small cell lung cancer in Thailand. Lung Cancer. 2018;120:91-7. doi: http://dx.doi.org/10.1016/j.lungcan.2018.04.003.
135. Lu S, Zhang J, Ye M, Wang BA, Wu B. Economic analysis of ALK testing and crizotinib therapy for advanced non-small-cell lung cancer. Pharmacogenomics. 2016;17(9):985-94. doi: 10.2217/pgs-2016-0017. PubMed PMID: WOS:000379495200003.
136. Lu S, Ye M, Ding L, Tan F, Fu J, Wu B. Cost-effectiveness of gefitinib, icotinib, and pemetrexed-based chemotherapy as first-line treatments for advanced non-small cell lung cancer in China. Oncotarget. 2017;8(6):9996-10006. doi: https://dx.doi.org/10.18632/oncotarget.14310.
137. Zhan M, Zheng H, Xu T, Yang Y, Li Q. Cost-effectiveness analysis of additional bevacizumab to pemetrexed plus cisplatin for malignant pleural mesothelioma based on the MAPS trial. Lung cancer (Amsterdam, Netherlands). 2017;110:1-6. doi: https://dx.doi.org/10.1016/j.lungcan.2017.05.012.
138. Zheng H, Xie L, Zhan M, Wen F, Xu T, Li Q. Cost-effectiveness analysis of the addition of bevacizumab to chemotherapy as induction and maintenance therapy for metastatic non-squamous non-small-cell lung cancer. Clinical & translational oncology : official publication of the Federation of Spanish Oncology Societies and of the National Cancer Institute of Mexico. 2018;20(3):286-93. doi: https://dx.doi.org/10.1007/s12094-017-1715-1.
139. Zhou K, Wen F, Zhang P, Zhou J, Zheng H, Sun L, et al. Cost-effectiveness analysis of sensitive relapsed small-cell lung cancer based on JCOG0605 trial. Clinical & translational oncology : official publication of the Federation of Spanish Oncology Societies and of the National Cancer Institute of Mexico. 2017. doi: https://dx.doi.org/10.1007/s12094-017-1787-y.
140. Lee JM, Kim MJ, Phongkitkarun S, Sobhonslidsuk A, Holtorf AP, Rinde H, et al. Health economic evaluation of Gd-EOB-DTPA MRI vs ECCM-MRI and multi-detector computed tomography in patients with suspected hepatocellular carcinoma in Thailand and South Korea. Journal of Medical Economics. 2016;19(8):759-68. doi: 10.3111/13696998.2016.1171230.
141. Zhang P, Wen F, Li Q. FOLFOX4 or sorafenib as the first-line treatments for advanced hepatocellular carcinoma: A cost-effectiveness analysis. Digestive and liver disease : official journal of the Italian Society of Gastroenterology and the Italian Association for the Study of the Liver. 2016;48(12):1492-7. doi: https://dx.doi.org/10.1016/j.dld.2016.07.007.
142. Zhou J, Zhao R, Wen F, Zhang P, Tang R, Du Z, et al. Cost-effectiveness analysis of gemcitabine, S-1 and gemcitabine plus S-1 for treatment of advanced pancreatic cancer based on GEST study. Medical Oncology. 2015;32(4):121.
143. Zhou J, Zhao R, Wen F, Zhang P, Wu Y, Tang R, et al. Cost-effectiveness analysis of treatments for metastatic pancreatic cancer based on PRODIGE and MPACT trials. Tumori. 2016;102(3):294-300. doi: 10.5301/tj.5000499.
144. Dabanovic V, Kostic M, Jankovic S. Cost effectiveness comparison of dutasteride and finasteride in patients with benign prostatic hyperplasia--The Markov model based on data from Montenegro. Vojnosanitetski pregled. 2016;73(1):26-33.
145. Lv M, Kou B, Zhou Z-L, He W-B. A mathematical optimization model for efficient assignment of inpatients in an oncology center in China. Journal of BUON : official journal of the Balkan Union of Oncology. 2017;22(3):790-800.
146. Mandrik O, Ramos IC, Knies S, A M, Severens JL. Cost-effectiveness of adding rituximab to fludarabine and cyclophosphamide for treatment of chronic lymphocytic leukemia in Ukraine. Cancer Management and Research. 2015;7:279-89. doi: 10.2147/CMAR.S79258.
147. Udeh EI, Ofoha CG, Adewole DA, Nnabugwu II. A cost effective analysis of fixed-dose combination of dutasteride and tamsulosin compared with dutasteride monotherapy for benign prostatic hyperplasia in Nigeria: a middle income perspective; using an interactive Markov model. BMC cancer. 2016;16:405. doi: https://dx.doi.org/10.1186/s12885-016-2431-x.

148. Wu B, Yao Y, Zhang K, Ma X. RAS testing and cetuximab treatment for metastatic colorectal cancer: a cost-effectiveness analysis in a setting with limited health resources. Oncotarget. 2017;8(41):71164-72. doi: https://dx.doi.org/10.18632/oncotarget.17029.
149. Pichon-Riviere A, Glujovsky D, Garay OU, Augustovski F, Ciapponi A, Serpa M, et al. Oxytocin in Uniject Disposable Auto-Disable Injection System versus Standard Use for the Prevention of Postpartum Hemorrhage in Latin America and the Caribbean: A Cost-Effectiveness Analysis. PloS one. 2015;10(6):e0129044. doi: https://dx.doi.org/10.1371/journal.pone.0129044.
